# The Honey Bee Epigenomes: Differential Methylation of Brain DNA in Queens and Workers

**DOI:** 10.1371/journal.pbio.1000506

**Published:** 2010-11-02

**Authors:** Frank Lyko, Sylvain Foret, Robert Kucharski, Stephan Wolf, Cassandra Falckenhayn, Ryszard Maleszka

**Affiliations:** 1 Division of Epigenetics, DKFZ-ZMBH Alliance, German Cancer Research Center, Heidelberg, Germany; 2 ARC Centre of Excellence for Coral Reef Studies, James Cook University, Townsville, Australia; 3 Research School of Biology, the Australian National University, Canberra, Australia; 4 Genomics and Proteomics Core Facility, German Cancer Research Center, Heidelberg, Germany; University of Lausanne, Switzerland

## Abstract

In honey bees (*Apis mellifera*) the behaviorally and reproductively distinct queen and worker female castes derive from the same genome as a result of differential intake of royal jelly and are implemented in concert with DNA methylation. To determine if these very different diet-controlled phenotypes correlate with unique brain methylomes, we conducted a study to determine the methyl cytosine (mC) distribution in the brains of queens and workers at single-base-pair resolution using shotgun bisulfite sequencing technology. The whole-genome sequencing was validated by deep 454 sequencing of selected amplicons representing eight methylated genes. We found that nearly all mCs are located in CpG dinucleotides in the exons of 5,854 genes showing greater sequence conservation than non-methylated genes. Over 550 genes show significant methylation differences between queens and workers, revealing the intricate dynamics of methylation patterns. The distinctiveness of the differentially methylated genes is underscored by their intermediate CpG densities relative to drastically CpG-depleted methylated genes and to CpG-richer non-methylated genes. We find a strong correlation between methylation patterns and splicing sites including those that have the potential to generate alternative exons. We validate our genome-wide analyses by a detailed examination of two transcript variants encoded by one of the differentially methylated genes. The link between methylation and splicing is further supported by the differential methylation of genes belonging to the histone gene family. We propose that modulation of alternative splicing is one mechanism by which DNA methylation could be linked to gene regulation in the honey bee. Our study describes a level of molecular diversity previously unknown in honey bees that might be important for generating phenotypic flexibility not only during development but also in the adult post-mitotic brain.

## Abstract 2

Using genome-wide methylation profiles in honey bee queen and worker brains to understand how contrasting organismal outputs are generated from the same genotype.

## Author Summary

The queen honey bee and her worker sisters do not seem to have much in common. Workers are active and intelligent, skillfully navigating the outside world in search of food for the colony. They never reproduce; that task is left entirely to the much larger and longer-lived queen, who is permanently ensconced within the colony and uses a powerful chemical influence to exert control. Remarkably, these two female castes are generated from identical genomes. The key to each female's developmental destiny is her diet as a larva: future queens are raised on royal jelly. This specialized diet is thought to affect a particular chemical modification, methylation, of the bee's DNA, causing the same genome to be deployed differently. To document differences in this epigenomic setting and hypothesize about its effects on behavior, we performed high-resolution bisulphite sequencing of whole genomes from the brains of queen and worker honey bees. In contrast to the heavily methylated human genome, we found that only a small and specific fraction of the honey bee genome is methylated. Most methylation occurred within conserved genes that provide critical cellular functions. Over 550 genes showed significant methylation differences between the queen and the worker, which may contribute to the profound divergence in behavior. How DNA methylation works on these genes remains unclear, but it may change their accessibility to the cellular machinery that controls their expression. We found a tantalizing clue to a mechanism in the clustering of methylation within parts of genes where splicing occurs, suggesting that methylation could control which of several versions of a gene is expressed. Our study provides the first documentation of extensive molecular differences that may allow honey bees to generate different phenotypes from the same genome.

## Introduction

Many animal species have evolved the capacity to generate organisms with contrasting morphological, reproductive, and behavioral phenotypes from the same genome. However, the question of how such strikingly different organismal outputs occur with no standard genetic changes remains one of the key unresolved issues in biology.

The nutritionally controlled queen/worker developmental divide in the social honey bee *Apis mellifera* is one of the best known examples of developmental flexibility in any phylum. Despite their identical nature at the DNA level, the queen bee and her workers are strongly differentiated by their anatomical and physiological characteristics and the longevity of the queen [1]. Furthermore, the behaviors of queens and workers are remarkably divergent, varying from the navigational proficiency of foragers to the colony-bound omnipresent chemical influences of the queen which control many aspects of the colony's existence. A diet of royal jelly during larval development clearly influences the epigenetic status of the queen's cells without altering any of the hardwired characteristics of her genome. As a result, two contrasting organismal outputs, fertile queens and non-reproductive workers, are generated from the same genome.

Recently, we have shown that diet is not the only modulator of developmental trajectories in honey bees. By silencing the activity of DNA methyltransferase 3 (DNMT3), a key component of epigenetic machinery controlling global gene reprogramming, we were able to generate adult bees with queen characteristics [2]. This relatively simple perturbation of the DNA methylation system not only mimicked the dietary effect of royal jelly on phenotype but also changed the cytosine methylation pattern of an illustrator gene. Furthermore, analysis of gene expression in both queens and workers suggested that their alternative developmental pathways are associated with subtle transcriptional changes in a particular group of genes encoding conserved physio-metabolic proteins [2],[3]. These findings prompted us to examine the hypothesis that significant behavioral differences between queens and workers are partly underpinned by differences between their brain epigenomes that have arisen from basically identical genomes during development. The choice of brain tissue is critical because it is a non-dividing, largely diploid tissue and is thus free of any complications that arise from differential genomic replication that may characterize polytene and endopolyploid tissues (nearly all adult tissues of insects are non-diploid). In the context of methylomes, the use of whole bodies, or abdomens, creates an unacceptable mixture of methylomic signatures that simply cannot be deconvoluted in regards to function in any biologically meaningful manner.

We used bisulfite converted brain DNA of both castes together with Solexa (Illumina GA) sequencing technology [4] to generate a DNA methylation map at single-nucleotide resolution across the *Apis* genome. This powerful approach has recently been used to compare DNA methylation profiles across a group of selected species, including DNA from a worker honey bee whole body [5]. The results confirm the antiquity of DNA methylation in eukaryotes [6],[7] and provide more experimental evidence that this epigenomic modification is utilized in a lineage-specific manner [8]–[10].

Here we confirm that in contrast to heavily methylated mammalian genomes [11], only a small and specific fraction of the honey bee genome is methylated [5],[10],[12],[13]. Furthermore, the methylated cytosines occur in a group of genes showing a higher level of conservation than non-methylated genes. Nearly 600 of those genes show significant methylation differences in the brains of queens and workers, suggesting that their transcription might be epigenetically modulated in a context-dependent manner. Additional deep sequencing of selected genes in all three castes—queens, workers, and drones (haploid males)—suggests that brain methylation patterns are unique to each behavioral system. We discuss our findings in the context of epigenetic influences on global regulatory networks and their ability to generate contrasting phenotypic and behavioral outcomes from the same genome.

## Results

### Characterization of Brain Methylomes in Queens and Workers

The sequencing of bisulfite converted *Apis* DNA yielded a dataset of 131 million reads after filtration and quality checks, 68.5% of which were mapped to unique genomic regions. The total sequence output was 18.8 giga bases (10.2 Gb for the queen and 8.6 Gb for the worker) yielding a combined 20× coverage of the 260 Mb genome. Our reads also contained multiple coverage of thousands of unmethylated repeated elements (ALUs and mariners) giving false-positive rates of only 0.1% for the queen DNA and 0.2% for the worker DNA. Figure S1A shows the distribution of the coverage depth for all cytosines on both strands, whereas distribution of the CpG nucleotides is shown in Figure S1B. More than 90% of the 10,030,209 CpGs in the *Apis* genome were covered by at least two sequencing reads, allowing for the methylation status of individual sites to be determined with confidence.

The characteristics of the brain methylomes of queens and workers are shown in Tables 1 and 2. Three firm conclusions can be drawn. First, of the over 60 million cytosines that exist in the *Apis* genome, only approximately 70,000 are methylated. Second, nearly all the methylated cytosines occur in CpG dinucleotides. Third, the overriding majority of these methylated sites are in exons. Finally, the number of methylated cytosines in *Apis* is nearly three orders of magnitude lower than in the human genome [11]. This relatively small number of mCs overcomes the large technical hurdles that exist in both mammalian and plant genomes where the number of methylated sites that need to be examined in terms of their importance to biological phenomena is in the hundreds of millions.

**Table 1:** Cytosine DNA methylation in queens and workers in CG, CHG, and CHH genomic contexts (H = A, T, or C).

	Total	Methylated in Queens	Methylated in Workers	Methylated in Both Castes
CG	10,030,209	69,064	68,222	54,312
CHG	8,673,113	14	130	0
CHH	45,072,611	561	3,019^a^	0

The thresholds used for methylation calls are detailed in the Methylation Assessment section.

a Nearly all of the 3,019 CHH that were inferred to be methylated in worker brains on the basis of Solexa reads were found to be not methylated by an additional sequencing of selected amplicons using the 454 technology.

**Table 2:** Cytosine DNA methylation in CG dinucleotides (mCG) in the exonic, intronic, and “intergenic” regions of queens and workers.

Genomic Location	Queens	% mCGs	% of All mCGs	Workers	% mCGs	% of all mCGs
Exons	54,378	8.6	78.74	51,658	8.16	75.72
Introns	5,992	0.2	8.68	6,720	0.22	9.85
Introns + exons	60,370	1.64	87.41	58,378	1.57	85.57
Intergenic regions^a^	8,694	0.16	12.59	9,844	0.17	14.43

a The annotation of the *Apis* transcriptome is largely limited to the coding regions, and it is likely that some of the intergenic regions may correspond to untranslated segments of mRNAs.

**Figure 1:**
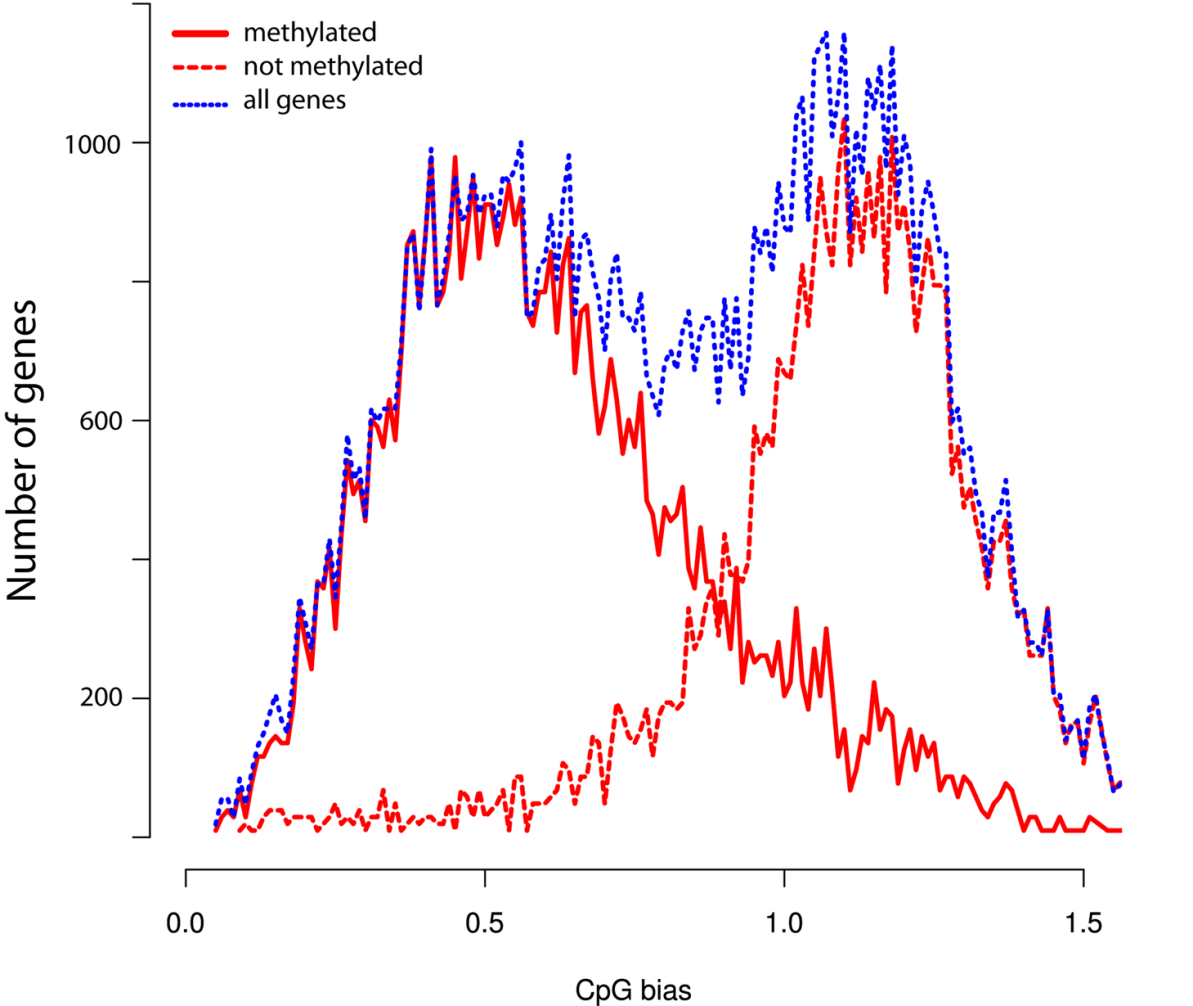
CpG (o/e) bias of protein-coding regions in the honey bee genome. Since the profiles for both queens and workers are virtually identical, only the queen profile is shown.

As shown in Table 1 the quantities of methylated CpGs (mCpGs) in queen and worker brain DNA are very similar, 69,064 and 68,222, respectively, with 54,312 mCpGs in common. Similarly, the methylation levels of mCpG are almost identical in both castes (Figure S2). Methylation in honey bees appears to be restricted to cytosines associated with CpG dinucleotides, with no significant non-CpG or asymmetric methylation detected in either genomic or mitochondrial DNA (Table 1). Therefore, we conclude that methylation at non-CpG sites is either extremely rare or non-existent in the honey bee genome. In accord with previous analyses [2],[5],[12],[13], methylated sites in *Apis* appear to be exclusively located in exons with only infrequent mCpGs detected in intronic regions (Table 2). Most importantly, the methylated exons reside in genomic regions with low CpG observed/expected (o/e) ratios (Figure 1), whereas non-methylated exons fall into the category with high CpG o/e ratios. This bimodal profile is consistent with previous predictions based on bioinformatics analyses [10],[12],[13] and reflects the propensity of methylated Cs to be converted over time to thymines, resulting in a lower than expected density of the CpGs in methylated genes. However, the total number of methylated genes in *Apis* revealed by genome-wide bisulfite sequencing is 5,854 instead of the 4,000 predicted to be methylated on the basis of local CpG bias. One reason for this difference might be that some genes do not display significant CpG depletion as a result of evolutionary pressure to maintain a particular protein coding sequence.

The genome-wide profiling of mCpGs confirms that methylated genes in *Apis* encode proteins showing a higher degree of conservation than proteins encoded by non-methylated genes [10]. Figures S3, S4, S5 and Table S1 show the results of our cross-species comparisons for methylated and non-methylated genes (Figure S3), for high-CpG and low-CpG genes (Figure S4), and high-CpG methylated and non-methylated genes (Figure S5). Most of the highly conserved genes are expected to be utilized by most tissues. In contrast, less conserved genes expressed in specialized tissues, such as those encoding odorant-binding proteins or odorant receptors, are not methylated (not shown). The repeated elements, ALUs, and mariners that harbor most of the DNA methylation content in humans and plants are *not* methylated in the bee genome, certainly not in the brain (Figures S6). Similarly, the multi-gene families encoding rRNAs and tRNAs, mitochondrial DNA, and CpG islands show no evidence of methylation in the brain (Figure S6). Lastly, while methylation of sub-telomeric regions has been shown to be important for the control of telomere length and recombination [14], the honey bee telomeres are also not methylated (not shown). The lack of methylation in ALUs and transposons has also been reported in a recent study performed on DNA extracted from a worker's whole body [5]. Given the proposed role of cytosine methylation in defense against genomic parasites in plants and vertebrates [7], the lack of methylation in ALU repeats and mariner transposons suggests that these mobile elements do not significantly impact on genome stability in honey bees. Indeed the bee genome contains an unusually small percentage of common types of transposons and retrotransposons found in other insects, possibly as a result of a strong selective pressure against mobile elements in male bees (drones) that develop from unfertilized eggs and carry a haploid set of chromosomes [15].

As in the human and *Arabidopsis* genomes [4],[11], methylation in *Apis* shows evidence of periodicity, although due to a much lower density of modified CpGs in this species the periodicity of 10 nucleotides (one helical DNA turn) is not obvious. However, a 3-base periodic pattern is clearly detectable, reflecting a preferential methylation of CpGs occupying the first and second position of the arginine codons (autocorrelation data in Figure S7).

### Detailed Analysis of Methylation Patterns in Selected Amplicons by Deep Bisulfite Sequencing

**Figure 2:**
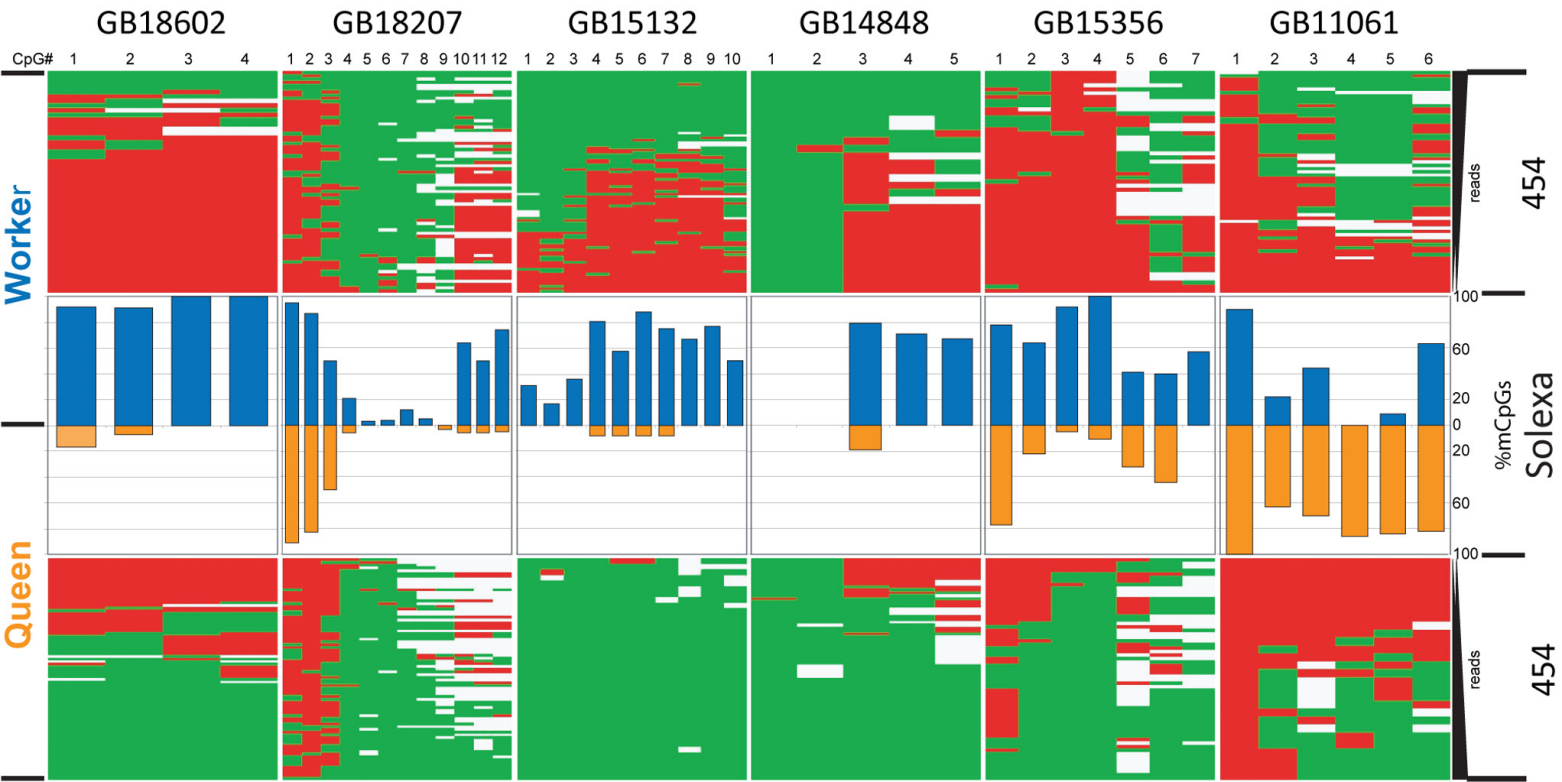
Comparison of CpG methylation profiles in differentially methylated genes generated by two technologies, Solexa genome-wide shotgun sequencing and 454 sequencing of PCR produced amplicons. The “heat maps” represent the 454 sequencing of PCR amplified segments, whereas the bars illustrate the Solexa reads. The eight nuclear genes for this experiment were chosen from the list of DMGs shown in Tables 3 and S2, taking into account the availability of convenient CpG-containing regions for primer design. Six genes are shown in this figure and the others in Figure 3. Gene annotations: GB18602 - membrane protein; GB18207 - cadherin; GB15132 - TAP42 (TOR signaling); GB14848 - clathrin assembly protein; GB15356 - syd, chromosome segregation; GB11061 - seryl-tRNA synthetase.

To validate our Solexa-based methylation results, we designed primers for selected regions of eight nuclear and four mitochondrial genes and re-sequenced the PCR-generated amplicons using 454 technology. As illustrated in Figure 2, the 454 sequencing profiles are essentially identical with the Solexa-based results. All nuclear genes show differential methylation in the brains of queens and workers, including those cases where the methylation is almost absent, such as GB18602 in queen brains (Figure 2). No methylation was detected by this approach in the four selected mitochondrial amplicons (not shown).

**Figure 3:**
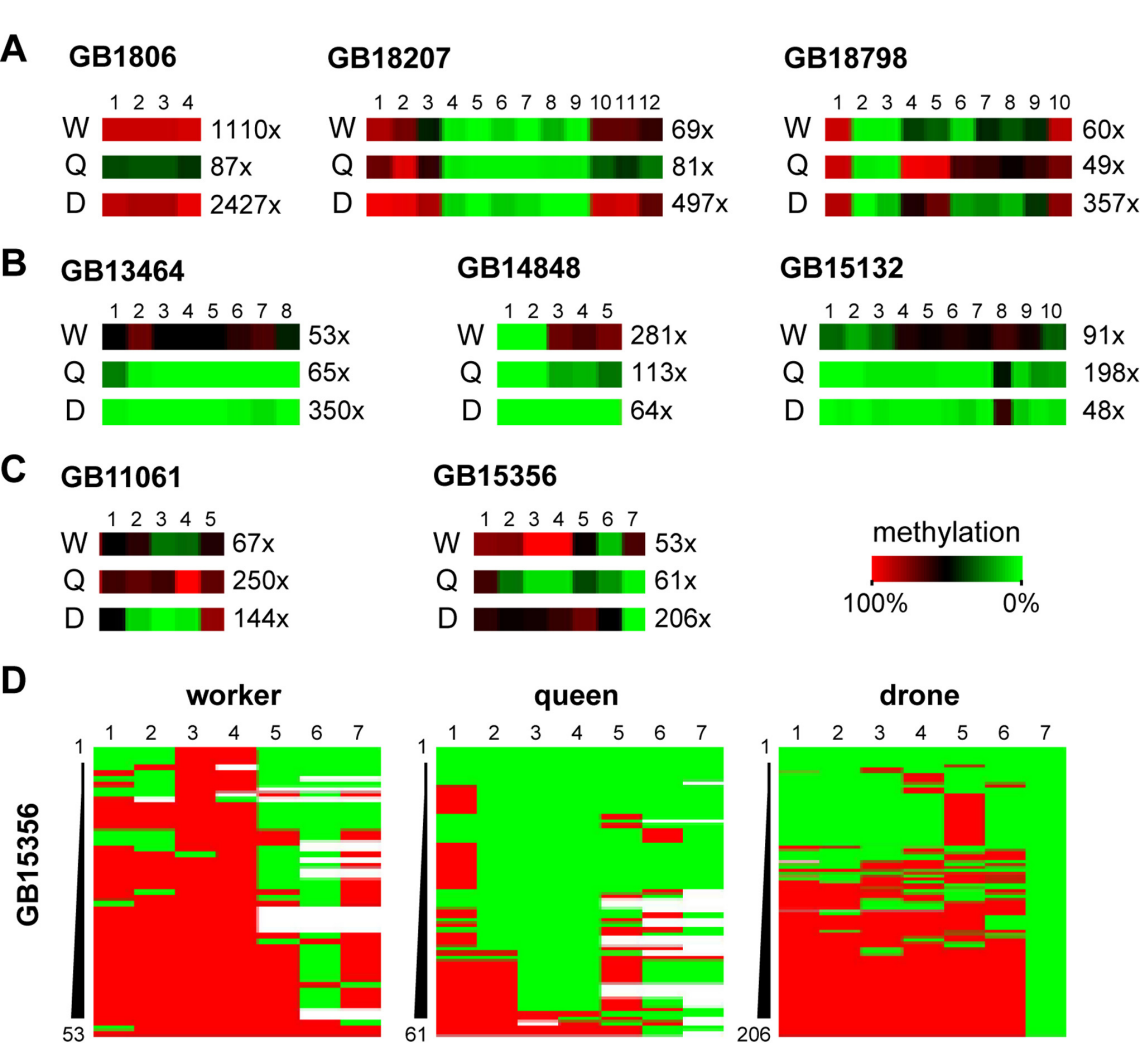
Detailed analysis of deep sequencing of selected genes. The bisulfite converted amplicons of selected genes were sequenced using 454 technology. The selection was based on differential methylation in brains of queens and workers, but DNA from male brains (drones) was also used in this experiment. The panels illustrate the uniqueness of brain methylation patterns in bees. 3A: Genes showing similar methylation patterns in workers and drones, but a distinct methylation pattern in queens. 3B: Genes with similar methylation patterns in queens and drones, but a distinct pattern in workers. 3C: Gene with distinct methylation patterns in all three castes. Panel 3D shows the full methylation heatmaps of GB15356. This result is discussed in the chapter “Detailed Analysis of Methylation Patterns in Selected Amplicons by Deep Bisulfite Sequencing.” Gene annotations: GB18798 - ubiquitin conjugation factor; GB13464 - RhoGAP93B. For other genes, see Figure 2.

To further expand our analysis, we increased the 454 bisulfite sequencing coverage of the eight nuclear genes selected for validation and also included DNA from drone brains. We obtained several thousand high-quality reads for 24 amplicons (eight genes in three castes), with the total coverage ranging from 48 to 2,427×. The results shown in Figure 3 reveal both the dynamics and uniqueness of the methylation patterns in each cast. Out of the eight genes with differential worker/queen methylation, three show similar methylation patterns in workers and drones, but a distinct methylation pattern in queens (Figure 3A). Three additional genes show similar methylation patterns in queens and drones, but a distinct pattern in workers (Figure 3B). Two out of eight analyzed genes (GB11061 - seryl-tRNA synthetase and GB15356 - syd, chromosome segregation; Figure 3C) show distinct methylation patterns in all three castes. The latter finding was also confirmed by the analysis of full methylation heatmaps of GB15356 (Figure 3D). GB15356 is strongly methylated in workers, with many reads showing complete methylation in the 5′-half of the amplicon (Figure 3D). In queens, GB15356 methylation is strongly reduced and many reads show no methylation at all. Intriguingly, drones show a bimodal methylation pattern with approximately half of the reads methylated and the other half unmethylated (Figure 3D). These results further illustrate caste-specific differences in methylation patterns and suggest a complex role of DNA methylation in the regulation of caste-specific epigenomic differences in the brain.

### Identification of Differentially Methylated Genes

To determine if there is a link between DNA methylation patterns and the striking morphological and behavioral polymorphisms of queen bees and workers, we examined the levels of CpG methylation in all annotated transcription units in both brains using high stringency criteria (Supporting Information). This approach generated a list of 561 differentially methylated genes (DMGs, Tables 3 and S2) showing significant methylation differences between the two castes. With the exception of highly expressed genes encoding ribosomal proteins, DMGs in *Apis* are expressed at low or moderate levels across all analyzed tissues (Tables 3 and S2). In several cases their transcriptional activities were found to be significantly up-regulated in some tissues relative to others. For example, the expression of 3-hydroxyl-CoA dehydrogenase (GB13368) is much higher in the larva than in the adult brain, and RNA-binding protein (GB12560) is significantly up-regulated in the ovaries relative to other tissues (Table 3). Almost all DMGs encode highly conserved, well-characterized proteins that have been implicated in core processes such as metabolism, RNA synthesis, nucleic acids binding, and signal transduction (Table S2). While a number of genes could not be clearly assigned to functional categories, their high level of conservation across phyla indicates that they are nevertheless likely to be involved in essential cellular processes (e.g. GB18943, GB13480, and GB18037). Several differently methylated genes encode proteins previously shown to be involved in either brain development or activity-dependent neural functions in both vertebrates and invertebrates. These include the Ephrin receptor GB1258516 [16], a nicotinic acetylcholine receptor GB19703, “no extended memory” GB16408 that is encoded by cytochrome B561 in *Drosophila*, two NMDA receptors GB19334 and GB15722, and a membrane channel GB12287 that mediates cell adhesion. When defective, GB12287 results in the “big brain” phenotype (Table S2). We note that Dynactin, used in our previous study [2] to illustrate the methylation differences between the two castes during larval growth in both royal jelly-fed and RNAi-treated individuals, does not show differential methylation in the brain. However, two genes, GB11197 and GB13866, encoding proteins associated with the large Dynein complex to which Dynactin also belongs are differentially methylated in the brain. Thus, the multi-protein Dynein complex appears to be epigenetically modulated during larval growth and in adult brains.

**Table 3:** Differentially methylated genes in brains of queens and workers.

				Relative	Expression^b^			
Gene ID^a^	No. of CpGs	Antenna	Brain	HPG	Larva	Ovary	Thorax	Gene Annotation
GB18602	30	1	1	1	1	1	1	Transmembrane protein YhhN
GB18303	13	1	1	1	1	1	1	Activator protein of Rab-like small GTPases
GB13368	9	2	1	2	10	1	3	3-hydroxyacyl-CoA dehydrogenase, NAD-binding
GB13215	34	1	1	1	1	1	1	Glycine cleavage system P-protein,
GB15588	9	1	1	1	1	1	1	Low-density lipoprotein receptor domain class A
GB15132	24	1	1	1	1	1	1	TAP42 (regulates the TOR signaling pathway)
GB12560	12	1	1	1	1	9	1	RNA-binding protein
GB11648	13	1	1	1	1	2	1	Catalase
GB19645	12	1	1	1	1	1	1	Phosphodiesterase 6
GB12929	39	1	1	1	1	1	1	Paralytic - Na channel
GB11421	31	1	1	1	1	1	1	Tight junction associated protein
GB19503	33	1	1	1	1	1	1	Heat shock protein 8
GB13740	24	1	1	1	1	1	1	Dysfusion, TF with PAS domain
GB10394	8	1	1	1	1	1	1	TNF-receptor-associated factor 1
GB16628	9	10	6	8	10	10	10	Ribosomal protein L6

Only the top 15 genes are shown; see Table S2 for list of 561 genes that fall into this category. Based on microarray data from Foret et al. [10]. The genome assembly v.02 was used throughout this study.

a GB numbers refer to the proteins at BeeBase: genomes.arc.georgetown.edu/drupal.

b Genes were ranked into 10 bins based on their expression levels from low (1) to high (10).

### CpG Bias and Epigenetic Modulation

Recently, Elango et al. [13] on the basis of bioinformatic analyses of a dataset of differentially expressed genes in brains of queens and workers proposed that “high-CpG genes in *A. mellifera* generally are more prone to epigenetic modulation than low-CpG genes.” We have tested this hypothesis using our new caste-specific brain methylome data. The results summarized in Table S3 suggest that (a) the methylation of a gene is a decreasing function of its CpG richness (Figure S8), (b) the “caste-specific genes” [13] that are methylated have a lower CpG content than the non-methylated genes (Table S3), and (c) DMGs are over-represented in the low CpG genes (Table S3). Therefore, our results do not support the hypothesis of Elango et al. [13]. However, it is noteworthy that although the DMGs are generally CpG-depleted, they tend to be less CpG-depleted than those genes that are not differentially methylated (Table S3). This intermediate CpG density observed in DMGs underscores the uniqueness of this class of genes and suggests that they might be methylated in a distinct manner from the rest of methylated genes. This class of genes showing differential patterns of methylation associated with phenotypic polymorphism is thus of special importance in the study of complex context-dependent phenotypes.

### Unraveling the Link between CpG Methylation and Splicing

**Figure 4:**
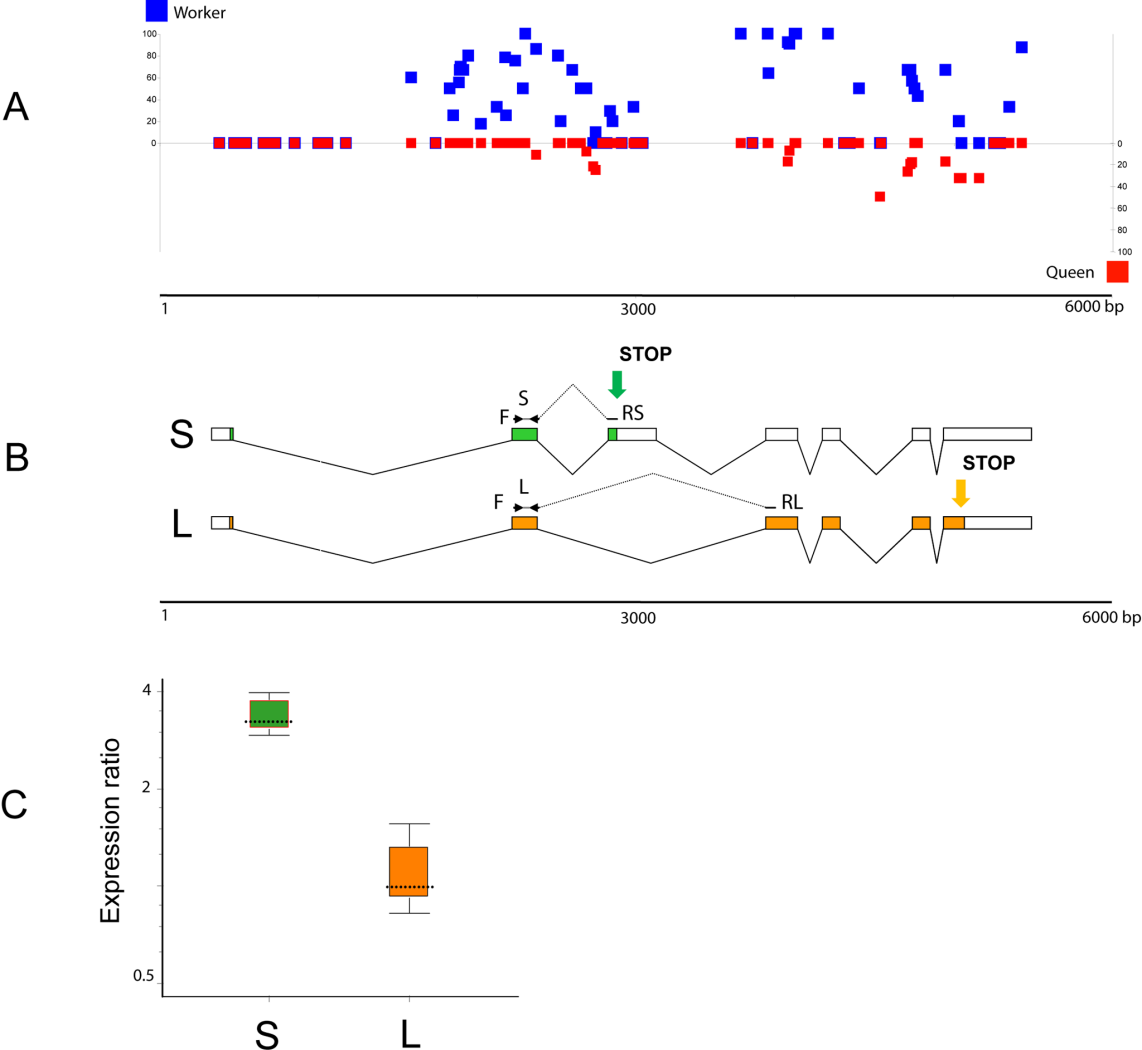
Expression profile of an alternatively spliced and differentially methylated gene GB18602 in queen and worker brains. (A) The CpG methylation pattern indicating the level of methylation for individual CpGs (blue squares, workers; red squares, queens). (B) Gene model of GB18602 showing the two spliced variants S (short protein) and L (long protein) and the positions of PCR primers used for variant-specific amplifications. The green and orange arrows indicate the positions of two alternative Stop codons. (C) Relative expression of the two spliced variants in brains of queens and workers examined by real-time PCR. The level of transcript S (green) encoding the truncated protein is significantly up-regulated in the queen brain, whereas the L variant (orange) is expressed at the same level in both castes. The queen expression represents a combined set of data from three independent queen samples: 4 mo old (1 brain), 12 mo old (2 brains), and swarm queens of unknown age (2 brains). Workers were 8 d old (6 brains in 3 replicates). The reference gene was calmodulin [2]. Whisker-box plot of expression ratio values: dotted line, median value; box, inter-quartile range of values; whiskers, outer 50% of observations. For more details, see Table S4.

To explore the relationship between differential methylation and expression patterns in queens and workers, we examined in more detail the first gene on the DMG list (GB18602) encoding a putative transmembrane protein with the YhhN domain conserved from bacteria to mammals. Figure 4 shows the distribution of mCpGs against the GB18602 gene model (Figure 4A and 4B) and the relative expression of two spliced variants in both castes (Figure 4C). The L variant (L) encoding a long protein shows identical expression levels in both queens and workers, whereas the S variant (S) encoding a short protein is significantly up-regulated in queen relative to worker brain (Figure 4C). The majority of the differentially methylated sites in the GB18602 locus map to the region spanning the additional cassette-exon that contains a Stop codon for the short protein encoded by the S transcript, suggesting a correlation between methylation and the outcome of alternative splicing of this gene in *Apis*. The increased level of methylation spanning the conditional splicing event (insertion or skipping of the cassette-exon) in the worker brain may impede the inclusion frequency of this exon into the mature transcript. Since the L variant is expressed at the same levels in both castes, the increased methylation in workers appears to be specifically affecting splicing, but not transcription. The observed differential pattern of expression of both transcripts in the brains of queens and workers (Figure 4C) supports this idea. Although the function of this gene is not known, the expression profiles of the *Drosophila melanogaster* ortholog CG7582 suggest that it encodes a protein involved in fat and sugar metabolism [17]. In the fly, which has no CpG methylation, this gene is not alternatively spliced and shows the highest levels of expression in the nervous system (FlyAtlas.org). In contrast, the human ortholog of GB18602, designated TMEM86A, produces alternatively spliced variants, including one encoding a truncated protein similar to the honey bee variant S. In addition to GB18602 we found numerous other examples of methylated genes in *Apis* in which most or even all clusters of mCpGs show a non-random, highly significant tendency to be near differentially spliced exons (Figure S9). Another salient finding relevant to methylation of intron-containing genes is the differential methylation of the multi-gene histone family in *Apis*. As illustrated in Table 4 and Figure S10, all intron-containing histone genes are methylated, whereas intronless histone genes show no evidence of methylation. It is noteworthy that the methylated histone genes in *Apis* belong to a distinct class of histone variants. Unlike the canonical histones these variants are expressed constitutively and independently of replication and act as multifunctional regulators in a range of processes including DNA repair, transcription initiation and termination, meiotic recombination, etc. [18]. It is believed that they represent lineage specific innovation that is important for each organism's evolutionary specialization [18].

**Table 4:** Annotation of the histone gene family in *Apis mellifera*.

Class	Proposed Subclass	Type	Apis Histone Genes	NCBI RefSeq mRNA	Proposed Gene ID	Proposed Protein ID	Splicing Status	Methyl CpG
**H1**	H1	canonical	GB12700^a^	XM_001121111	H1.1	H1.A		
			GB12218	XM_001122184	H1.2	H1.B		
**H2A**	H2A	canonical	GB18806	XM_001120186	H2A.1	H2A.A		
			GB12818	XM_001120346	H2A.2	H2A.B		
			GB13800	XM_001119899	H2A.3	H2A.C		
			N/A^c^	XM_001120934	H2A.4	H2A.D		
	H2A.X	**variant**	GB18954	XM_624697	H2A.X	H2A.X	**Spliced**	**Yes**
	H2A.Z/H2AV	**variant**	GB12991	XM_624164	H2A.Z	H2A.Z	**Spliced**	**Yes**
	Pseudogene		Pseudogene		ψH2A			
**H2B**	H2B	canonical	GB12700^a^	XM_001120238	H2B.1	H2B.A		
			GB13012	XM_001120889	H2B.2	H2B.B		
			N/A^c^	XM_001122218	H2B.3	H2B.C		
			GB12922^b^	XM_001119846	H2B.4	H2B.D		
			GB11889	XM_001120014	H2B.5	H2B.D		
**H3**	H3	canonical	GB11223	XM_001120304	H3.1	H3		
			GB14620	XM_001121026	H3.2	H3		
			N/A^c^	XM_001120132	H3.3	H3		
	H3.3	**variant**	GB12948	XM_001120696	H3.3.1	H3.3	**Spliced**	**Yes**
			GB11228	XM_624496	H3.3.2	H3.3	**Spliced**	**Yes**
	CENPA	**variant**	GB18566	N/A	CENPA	CENPA	**Spliced**	**Yes**
**H4**	H4	canonical	GB20104	XM_001120066	H4.1	H4		
			GB12644^a^	XM_01119948	H4.2	H4		
			GB14107	XM_001120988	H4.3	H4		
			GB17789	NM_001011609	H4.4	H4		

See Figure S10 for additional details. These Bee Base protein entries are either incorrect or missing:

a chimeras,

b truncated,

c not available.

## Discussion

The discovery of a functional DNA methylation system in honey bees and other invertebrates [1],[7]–[10],[19] has brought a fresh perspective to the study of epigenetic regulation of development and behavior. It reinforced the view that this covalent modification of DNA is an ancient and widely utilized evolutionary mechanism that was present in the basal Metazoa and has been recruited to serve diverse functions in modern organisms, including regulation of gene expression, cell differentiation, and silencing of transposons [20]–[22]. However, the trajectories from methylation changes to complex phenotypes are indirect, multi-level, and virtually unknown. For example, the hundreds of millions of methylated cytosines in the human genome and their large variation in different cell types in vivo pose a major challenge to uncovering those changes causative to phenotype. By contrast, the honey bee *Apis mellifera* shares its basic methylation enzymology with humans, yet as shown in this and other studies [5],[10],[12],[13] only a small and specific fraction of its genome is methylated. The present results show that honey bees utilize methyl tags to mark a core of mostly conserved and ubiquitously expressed critical genes whose activities cannot be switched off in most tissues. Recent data suggest that in spite of their permanent expression these genes might not be required at the same level throughout development, or under changing environmental conditions [23]–[25].

In honey bees, feeding of newly hatched larvae destined to become queens with royal jelly leads to metabolic acceleration and increased growth driven by global but relatively subtle changes in the expressional levels of a large number of ubiquitous genes [2],[3],[10]. These initial stages of larval development are later followed by the activation of more specific pathways to lay down caste-specific structures [3],[10]. Interestingly, adult queen bees continue to be fed royal jelly, suggesting that this highly specialized diet is important for maintaining their reproductive as well as behavioral status. One possibility is that adult queens adjust their brain methylomes according to external instructions from their diet. One of the ingredients of royal jelly, phenyl butyrate [26], is a known histone deacetylase inhibitor and growth regulator that has been implicated in improving cognitive deficits in mice [27] and in life extension of *Drosophila* [28]. Although the significance of phenyl butyrate in royal jelly is not yet understood, it is conceivable that this complex diet evolved to provide two important functions for honey bees. It primarily serves as the source of nutrients for queen development but also as the regulator of epigenetic networks controlling gene expression in the brain. In addition to having different morphologies, reproductive capacities, and distinct behaviors, the genetically identical queen and worker honey bees also have different synaptic densities in their brains. In a recent study, Groh and Rossler [29] proposed that such developmental, diet-induced heterochrony results in fewer synapses in olfactory centers in queens, which may result in poorer performance on olfactory learning tasks compared to workers.

Recent studies using rodent models provided strong support for an idea that the nervous system has co-opted epigenetic mechanisms utilized during development for activity-dependent brain functions, including the generation and maintenance of long-term behavioral memories in adulthood [30],[31]. Not surprisingly, DNA methylation has also been found to be involved in memory processing in honey bees [32], highlighting the significance of this epigenomic setting in conserved brain functions. These findings also provided evidence that DNA methylation, once believed to be an inert process after cellular differentiation, is dynamically regulated in the adult brain. Although both DNA methylation and chromatin remodeling have been implicated in these processes, the specific biological mechanisms underlying such adaptations remain largely unknown.

Our study provides experimental evidence that at least 560 differentially methylated ubiquitously expressed genes are involved in generating molecular brain diversity in female honey bees. Although it is still unclear how methylation might be linked to the gene regulatory networks, it has been proposed that DNA methylation together with changes in the histone profiles has the capacity to adjust DNA accessibility to cellular machinery by changing chromatin density [33]–[35]. Our findings support this notion and suggest that this mechanism provides an additional level of transcriptional control to fine tune the levels of messenger RNAs, including differentially spliced variants, encoded by the conserved genes. The association of mCpG clusters with alternatively spliced exons and genes containing introns in *Apis* is reminiscent of the distribution of mCpGs around the exon/intron junctions in human genes [36]. Epigenetic control of both splicing and mRNA levels might be utilized in different lineages, suggesting that a direct relationship between gene methylation and transcription is a widely spread phenomenon in both the animal and plant kingdoms [8],[37].

Cytosine methylation may interact with other epigenetic features, such as distinctive histone modification signatures that have been shown to correlate with the splicing outcome in a set of human genes [33]–[35]. The correlation between methylation and splicing is further highlighted by the differential methylation of two classes of histone genes in *Apis*. We find that only intron-containing histone variants are methylated, whereas intronless canonical histone genes are not methylated. Interestingly, histone variants have been implicated in multiple conserved roles in eukaryotes [18] and therefore are part of the cellular maintenance systems together with other ubiquitously expressed genes. In a broader context, methylated cytosines may specify information to set up, proliferate, and regulate splicing patterns during cellular processes such as development and differentiation.

Thus, rather than switching the genes on and off by promoter methylation, the intragenic methylation in *Apis* operates as a modulator of gene activities. As a result the entire topology of a complex brain network can be reprogrammed by subtle adjustments of many genes that act additively to produce a given phenotype [38]. Such adjustable DNA methylation levels generating variability in the transcriptional output of methylated genes could underlie genetically inherited propensity to phenotypic variability in accord with the recently proposed model of stochastic epigenetic variations as a heritable force of evolutionary change [39].

The technical advantages of the low number of methylated cytosines in the genome, together with diet-controlled phenotypes arising from the same genome, make the honey bee an extremely tractable, simplified in vivo system in which to examine fundamental principles underpinning transitions from methylomes to organismal plasticity. In particular, the absence of promoter methylation in honey bees brings into focus gene body methylation as an important mechanism controlling various aspects of transcription. The utility of honey bees for understanding the intricacies of this process in the behavioral context can now be experimentally tested.

## Materials and Methods

### Source of DNA

Total DNA was extracted from dissected gland-free brains of 50 age-matched egg-laying queens (2.5 wk old) and from fifty 8-d-old workers. These individuals represent early stages of the reproductive life of queen bees and mature young workers capable of performing foraging tasks [19].

### Sequencing of Bisulfite Converted DNA Libraries Using the Solexa GAIIx Platform (Illumina)

5 µg of high molecular weight DNA were used for fragmentation using the Covaris S2 AFA System in a total volume of 100 µl. Fragmentation-run parameters: Duty cycle 10%; Intensity: 5; Cycles/burst: 200; Time: 3 min; number of cycles: 3, resulting in a total fragmentation-time of 180 s. Fragmentation was confirmed with a 2100 Bioanalyzer (Agilent Technologies) using a DNA1000 chip. Fragment sizes were 140 bp on average for queen and worker DNAs, respectively. The fragmented DNAs were concentrated to a final volume of 75 µl using a DNA Speed Vac. End repair of fragmented DNA was carried out in a total volume of 100 µl using the Paired End DNA Sample Prep Kit (Illumina) as recommended by the manufacturer. For the ligation of the adaptors, the Illumina Early Access Methylation Adaptor Oligo Kit and the Paired End DNA Sample Prep Kit (Illumina) were used, as recommended by the manufacturer. For the size selection of the adaptor-ligated fragments, we used the E-Gel Electrophoresis System (Invitrogen) and a Size Select 2% precast agarose gel (Invitrogen). Each fragmented DNA was loaded on two lanes of the E-gel. Electrophoresis was carried out using the “Size Select” program for 16 min. According to the standard loaded (50 bp DNA Ladder, Invitrogen), 240 bp fragments were extracted from the gel, pooled, and directly transferred to bisulfite treatment without further purification. For the bisulfite treatment we used the EZ-DNA Methylation Kit (Zymo) as recommended by the manufacturer with the exception of a modified thermal profile for the bisulfite conversion reaction. The conversion was carried out in a thermal cycler using the following thermal profile: 95°C for 15 s, 50°C for 1 h, repeat from step 1, 15×, 4°C for at least 10 min. The libraries were subsequently amplified, using the Fast Start High Fidelity PCR System (Roche) with buffer 2, and Illuminas PE1.1 and PE2.1 amplification primers. PCR thermal profile: 95°C for 2 min, 95°C for 30 s, 65°C for 20 s, 72°C for 30 s, then repeat from step 2, 11×, 72°C for 7min, hold at 4°C. PCR reactions were purified on PCR purification columns (MinElute, Qiagen) and eluted in 20 µl elution buffer (Qiagen).

### Validation of the Libraries

1 µl of the libraries were analyzed on a 2100 Bioanalyzer (Agilent Technologies) using a DNA1000 chip. The fragment sizes were 240 bp and 243 bp for the queen and worker libraries, respectively. The estimated concentrations of the libraries were 0.8 ng/µl for the queen library and 5.8 ng/µl for the worker library.

### Sequencing and Analysis

We used 8 pM of single stranded DNA per lane for Solexa sequencing. In total we sequenced 6 lanes. Worker: 1. single end - 36 bp - 10,187,567 reads (×2); 2. paired end - 76 bp - 7,960,842 reads (×2); 3. paired end - 76 bp - 7,444,938 reads (×2); 4. paired end - 76 bp - 11,642,135 reads (×2). Queen: 1. paired end - 76 bp - 16,752,247 reads (×2); 2. paired end - 76 bp - 16,778,784 reads (×2). For sequencing we used a Solexa Genoma Analyzer GAIIx with a v2 Paired End Cluster Generation Kit - GA II (Illumina) and v3 36 bp Cycle Sequencing Kits (Illumina). Extraction of sequences was done using Illumina Pipeline v1.4 software. Image analysis and basecalling was done using Illumina SCS v2.5 software.

### Mapping

Reads were mapped using BSMAP-1.0240 with minor modifications [40]. A number of trimming and mapping options were assessed, and the conditions yielding the highest genome coverage depth was used for further processing (-s 12 -v 5 -k 6, for word size, number of mismatches, and number of words). Only the reads mapping uniquely were used. Mapping was carried out on a Linux cluster running Debian 5.0 (lenny).

### Methylation Assessment

To increase the accuracy of methylation calls, only those cytosines fulfilling neighborhood quality standards NQS41 were counted [41]; namely, we only took into account bases of quality 20 or more, flanked by at least three perfectly matching bases of quality 15 or more. Deamination efficiency was assessed using the observation that the genomic repeats are not methylated in the honeybee (Figure S3). The deep coverage of these repeated sequences allowed us to estimate that the deamination rate is 99.76% for the queens and 99.71% for workers. The methylation status of each cytosine was then assessed by comparing the number of methylated and non-methylated reads to a binomial distribution with a probability of success equal to the deamination rate and a number of trials equal to the number of reads mapping to that cytosine and adjusting the resulting *p* values for multiple testing with the method of Benjamini and Hochberg [42]. An adjusted *p* value of 0.05 was used as a threshold for methylation calls. All statistical computations were carried out using the R language (www.r-project.org).

Honeybee ESTs and predicted genes were loaded into a Mysql database and visualized with Gbrowse (www.gmod.org), where CpG methylation levels in queens and workers were added as separate tracks.

### Differential Methylation

Base-wise differences between queen and workers were estimated using Fisher exact tests. Gene-wise differences were assessed by generalized linear models of the binomial family, where methylation levels were modeled as functions of two categorical variables: caste and CpG position. *p* values were adjusted for multiple testing with the method of Benjamini and Hochberg [42].

### Amplicon Sequences Selection

Illumina sequencing and BSMAP mapping results were confirmed by 454 sequencing of a set of bisulfite amplicons. Amplicon sequences were selected using raw methylome data and the following criteria: minimum coverage - 5 mapped reads for each queen and worker sample; minimum 2 mCpGs within a maximum of ∼600 bp of sequence showing at least 50% difference in methylation levels between the two samples. In addition, four regions of mtDNA were selected. All primers and other details are listed in Table S4.

### Other Protocols

All molecular protocols are described elsewhere [2],[9],[10],[43].

## Supporting Information

Figure S1 **Coverage of all cytosines.** (A) Cumulative distribution of the coverage of all cytosines, on either strand of the genome, in workers and queens. On the *x*-axis, coverage refers to the coverage depth that is the number of reads uniquely mapped to a given cytosine. The *y*-axis is the cumulative distribution; for instance, approximately 50% of all cytosines are covered by less than 5 reads, and about 80% are covered by less than 10 reads. (B) Cumulative distribution of the coverage of all CpGs in the genome, in workers and queens. On the *x*-axis, coverage refers to the coverage depth that is the number of reads uniquely mapped to a given CpG dinucleotide. The *y*-axis is the cumulative distribution (for instance, approximately 50% of the CpGs are covered by less than 15 reads, and about 80% are covered by less than 25 reads).
(0.41 MB PDF)

Figure S2 **Methylation levels of methylated CpGs.** Distribution of the methylation level of methylated CpGs. The methylation level is the proportion of methylated reads mapping to a given CpG. Over 30% of the CpGs are fully methylated.
(0.16 MB PDF)

Figure S3 **Number of methylated and non-methylated Apis genes with BLAST hits to different species at various E-value thresholds.** The amino acid sequences of the genes were compared. Fisher exact tests were conducted to assess whether significantly more methylated genes have a BLAST hit than non-methylated genes. Statistically significant tests at the 5% level are denoted with a star, and non-significant tests are shown with a dot. The details of this analysis can be found in Table S3.
(0.13 MB PDF)

Figure S4 **Number of high and low CpG honey bee genes with BLAST hits to different model species at various E-value thresholds.** The amino acid sequences of the genes were compared. Fisher exact tests were conducted to assess whether significantly more low CpG genes have a BLAST hit than high CpG genes. Statistically significant tests at the 5% level are denoted with a star, and nonsignificant tests are shown with a dot. The details of this analysis can be found in Table S3.
(0.13 MB PDF)

Figure S5 **Number of high CpG methylated and non-methylated honey bee genes with BLAST hits to different model species at various E-value thresholds.** The amino acid sequences of the genes were compared. Fisher exact tests were conducted to assess whether significantly more high CpG methylated genes have a BLAST hit than high CpG non-methylated genes. Statistically significant tests at the 5% level are denoted with a star, and non-significant tests are shown with a dot. The details of the analysis can be found in Table S3.
(0.13 MB PDF)

Figure S6 **Coverage (red) and methylation ratio (green) along various kinds of repetitive elements.** The methylation ratio is the proportion of the reads where a cytosine is either methylated or unconverted. The *y*-axes are logarithmic in base 10 (the *x*-axis is truncated to the nearest multiple of 50, just like the *y*-axis is truncated to the nearest integer).
(0.63 MB PDF)

Figure S7 **Periodicity of methylation patterns.** (A) Autocorrelation of CpG methylation status over 1 kb. (B) Autocorrelation over 100 bp. Figures A and B show that the correlation of methylation status of neighboring CpGs increases sharply between 1 bp and 20 bp, then drops rapidly between 40 bp and 100 bp, and then slowly fades away. CpGs within a neighborhood of 2 bp to 100 bp are thus more likely to share the same methylation status than more distant CpGs. (C) Fourrier transform of autocorrelation showing a clear periodicity peak at 33 cycles per 100 bp (every 3 bp). (D) Distribution of codon position of mCs, and distribution of methylation level depending on the position. These two panels indicate that the distance between methylated CpGs is often a multiple of three and that the methylated cytosine corresponds most frequently to the first nucleotide of an arginine codon.
(0.33 MB PDF)

Figure S8 **Correlation between CpG o/e and proportion of methylated CpGs.** Genes with a lower CpG content tend to have a higher proportion of methylated CpGs. The red line is a polynomial regression through the points. The Akaike Information Criterion for model selection and a (monotonously decreasing) polynome of degree three was identified as the best model.
(0.71 MB PDF)

Figure S9 **Distribution of methylated CpGs relative to splicing sites.** For 169 genes, each containing a single well-defined alternative splicing event, the distance of all mCpGs to the centre of the alternatively spliced intron was computed, and the median of all these distances was calculated. A null distribution of this median distance was constructed using a randomization procedure (Manly, 2007): the methylation status of mCpGs of these genes were randomly shuffled 1,000 times, and the corresponding median distances computed. The observed value (1,224) is smaller than the smallest of the null distribution (1,259); the probability of the methylated CpGs to be as close or closer to the alternatively spliced intron as in this dataset is thus less than 0.001.
(0.77 MB PDF)

Figure S10 **Annotation of the histone gene family in Apis mellifera showing the methylation profiles.** See Table 4 for details.
(0.34 MB PDF)

Table S1 **Sequence conservation of methylated and non-methylated genes.** (A) Number of high and low CpG Apis genes with blast hits to different species at various E-value thresholds. The amino acid sequences of the genes were compared. Fisher exact tests were conducted to assess whether significantly more low CpG genes have a blast hit than high CpG genes. (B) Number of methylated and non-methylated honey bee genes with blast hits to different model species at various E-value thresholds. The amino acid sequences of the genes were compared. Fisher exact tests were conducted to assess whether significantly more methylated genes have a blast hit than non-methylated genes. (C) Number of high CpG methylated and non-methylated honey bee genes with blast hits to different model species at various E-value thresholds. The amino acid sequences of the genes were compared. Fisher exact tests were conducted to assess whether significantly more high CpG methylated genes have a blast hit than high CpG non-methylated genes.
(0.19 MB DOC)

Table S2 **Differentially methylated genes in queens and worker brains.** A generalized linear model of the binomial family was used to identify genes that are differentially methylated between castes. The methylation level of each gene was modeled as a function of the caste and of each of its CpG dinucleotides. In the table, “Caste” indicates whether the caste is a statistically significant factor explaining differences in methylation levels, “CpG” represents the different dinucleotides of that gene, and “Caste * CpG,” the interaction factor, indicates whether the CpG dinucleotides behave differently between castes. GB numbers refer to the proteins at BeeBase: genomes.arc.georgetown.edu/drupal. Genes were ranked into 10 bins based on their expression levels from low (1) to high (10). No value in the relative expression column indicates those genes that are not represented on the microarray. Based on microarray data from Foret et al. [10].
(1.13 MB DOC)

Table S3 **Evaluation of the Elango et al. hypothesis.** (A) CpG o/e in methylated genes. (B) Differential methylation and differential gene expression.
(0.04 MB DOC)

Table S4 **Details on genes used for deep 454 sequencing.**
(0.03 MB XLS)

## Abbreviations

DMG: differentially methylated gene
DNMT3: DNA methyltransferase 3
mCpG: methylated CpG
o/e: observed/expected
