# Correction: The Honey Bee Epigenomes: Differential Methylation of Brain DNA in Queens and Workers

**DOI:** 10.1371/annotation/2db9ee19-faa4-43f2-af7a-c8aeacca8037

**Published:** 2011-01-11

**Authors:** Frank Lyko, Sylvain Foret, Robert Kucharski, Stephan Wolf, Cassandra Falckenhayn, Ryszard Maleszka

The data have been deposited in the NCBI Sequence Read Archive under the accession number SRA012457.

