# Supplementary figures and images for: The Honey Bee Epigenomes: Differential Methylation of Brain DNA in Queens and Workers
Source: PLoS Biol. 2010 Nov 2;8(11):e1000506. doi: 10.1371/journal.pbio.1000506 (PMC2970541; doi:10.1371/journal.pbio.1000506)

A

## Coverage of all cytosines

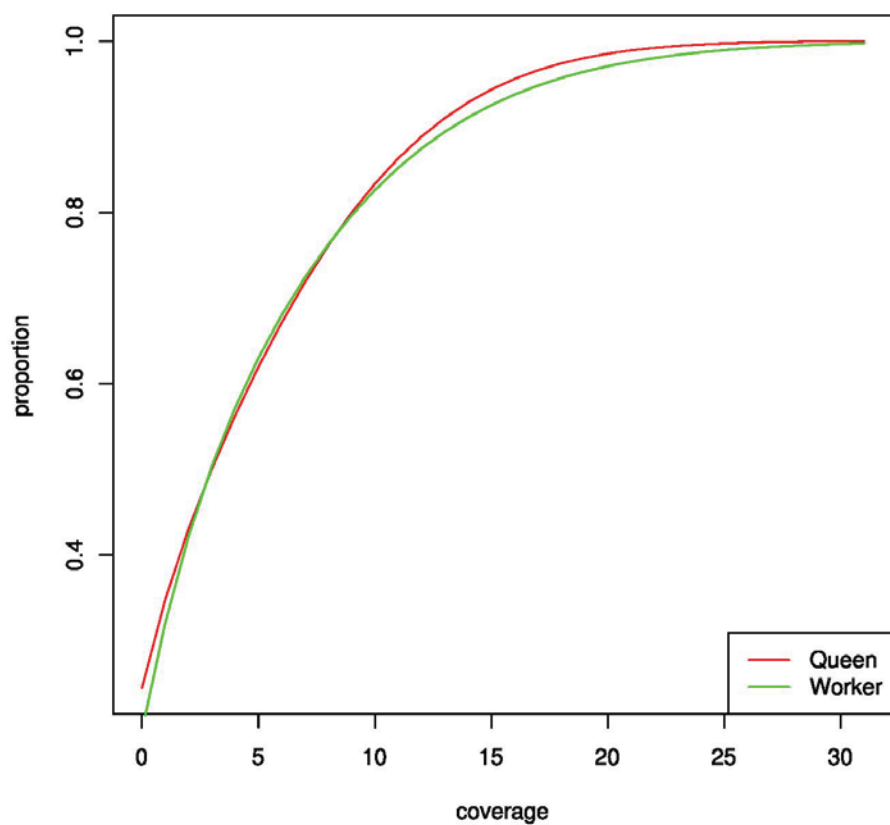

B

## Coverage of CpG dinucleotides

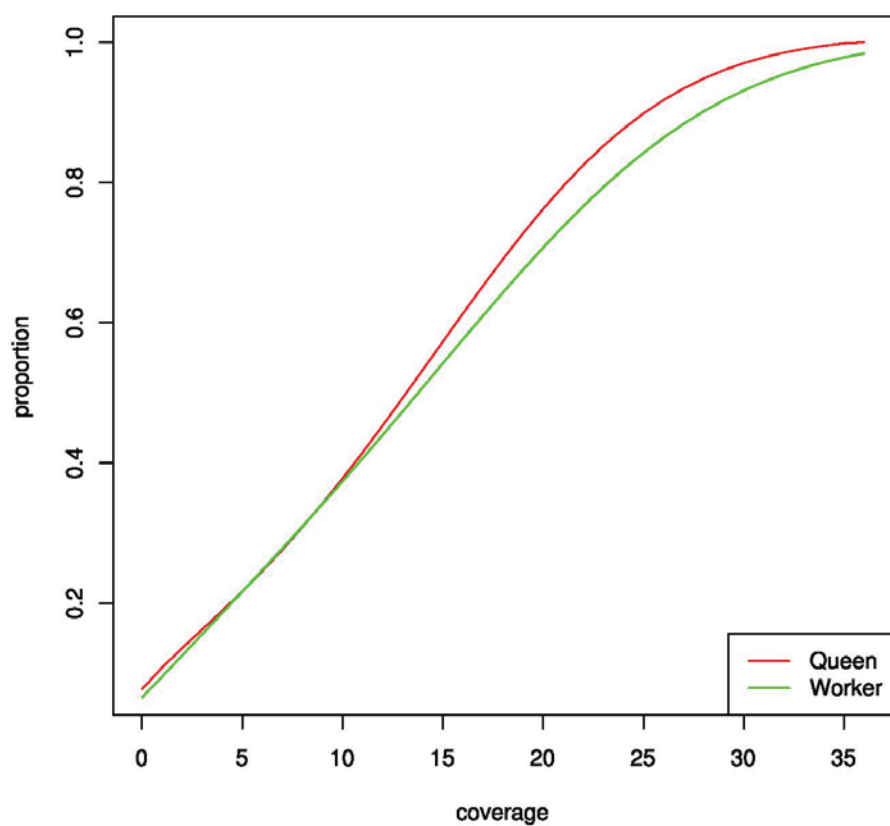

Supplement: Figure S1 — Coverage of all cytosines. (A) Cumulative distribution of the coverage of all cytosines, on either strand of the genome, in workers and queens. On the x-axis, coverage refers to the coverage depth that is the number of reads uniquely mapped to a given cytosine. The y-axis is the cumulative distribution; for instance, approximately 50% of all cytosines are covered by less than 5 reads, and about 80% are covered by less than 10 reads. (B) Cumulative distribution of the coverage of all CpGs in the genome, in workers and queens. On the x-axis, coverage refers to the coverage depth that is the number of reads uniquely mapped to a given CpG dinucleotide. The y-axis is the cumulative distribution (for instance, approximately 50% of the CpGs are covered by less than 15 reads, and about 80% are covered by less than 25 reads). (0.41 MB PDF) [file pbio.1000506.s001.pdf]

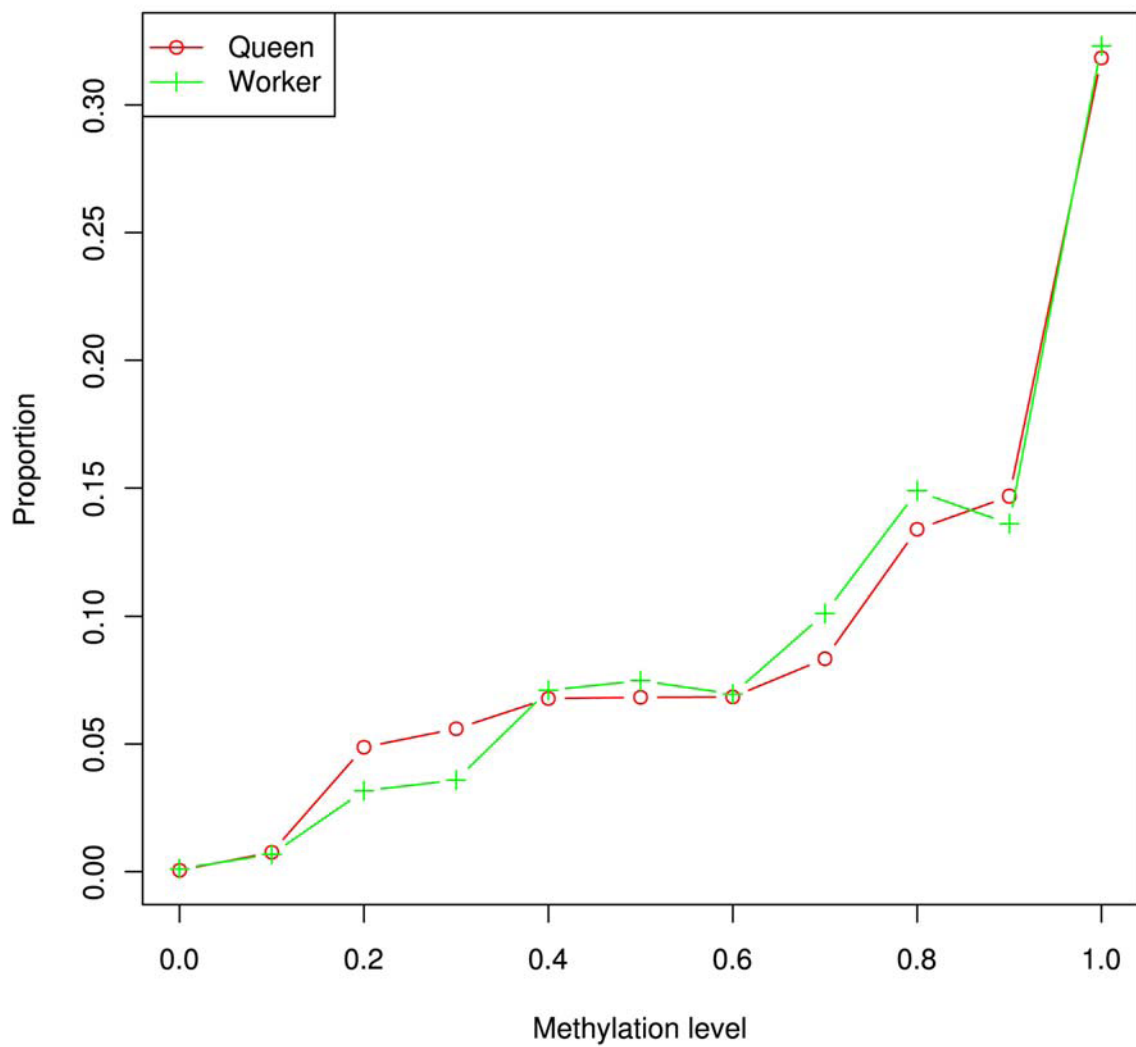

Supplement: Figure S2 — Methylation levels of methylated CpGs. Distribution of the methylation level of methylated CpGs. The methylation level is the proportion of methylated reads mapping to a given CpG. Over 30% of the CpGs are fully methylated. (0.16 MB PDF) [file pbio.1000506.s002.pdf]

A. thaliana

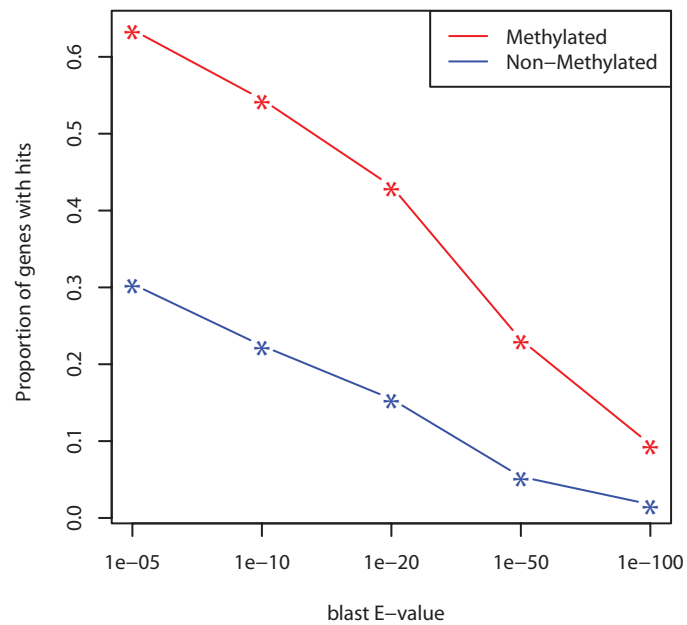

C. elegans

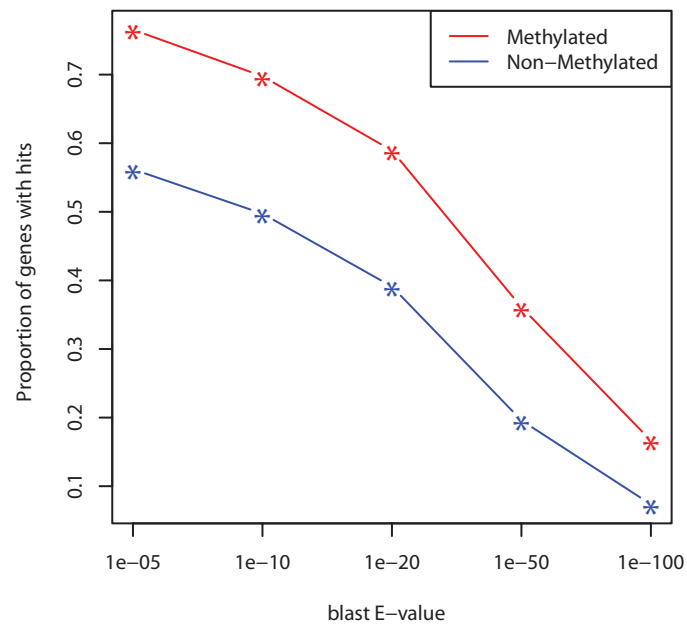

D. melanogaster

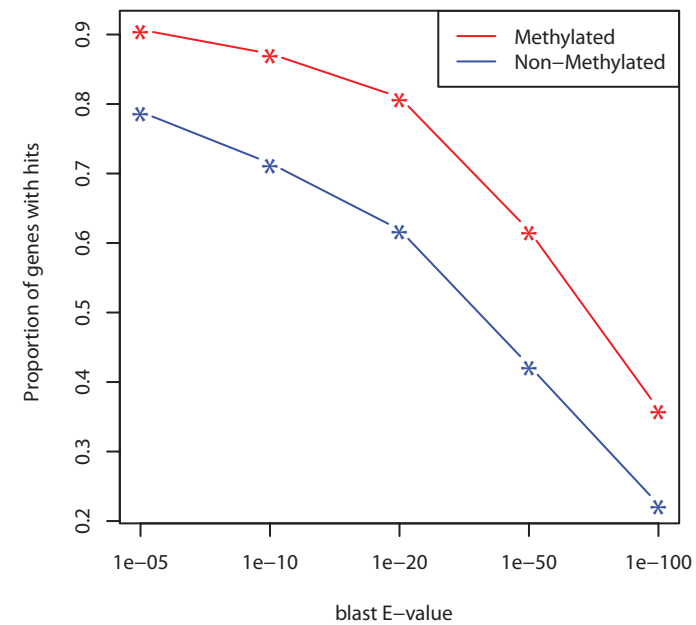

E. Coli

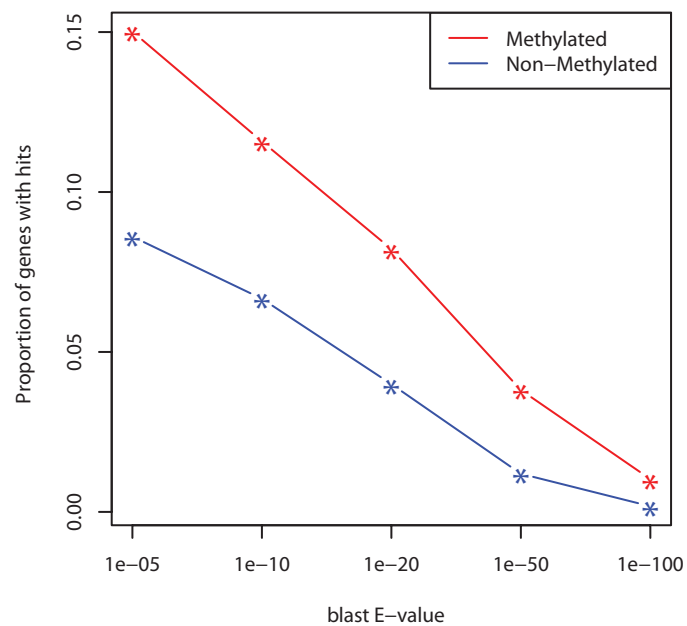

H. sapiens

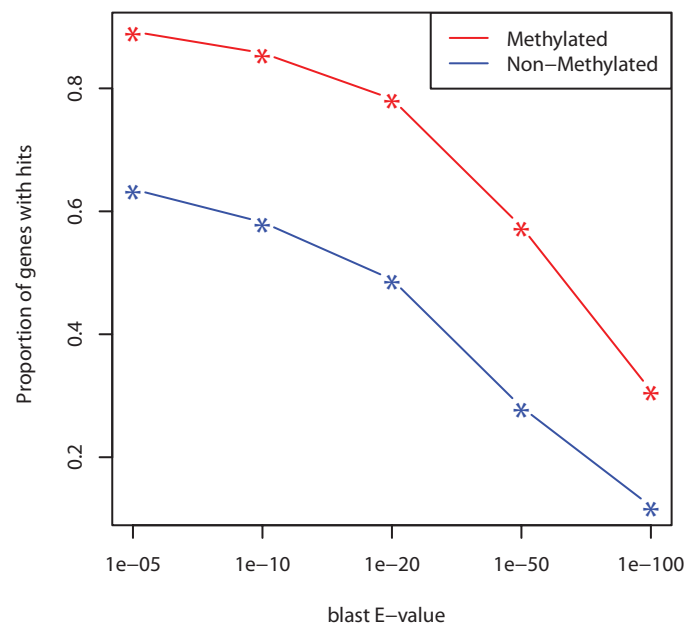

Supplement: Figure S3 — Number of methylated and non-methylated Apis genes with BLAST hits to different species at various E-value thresholds. The amino acid sequences of the genes were compared. Fisher exact tests were conducted to assess whether significantly more methylated genes have a BLAST hit than non-methylated genes. Statistically significant tests at the 5% level are denoted with a star, and non-significant tests are shown with a dot. The details of this analysis can be found in Table S3. (0.13 MB PDF) [file pbio.1000506.s003.pdf]

A. thaliana

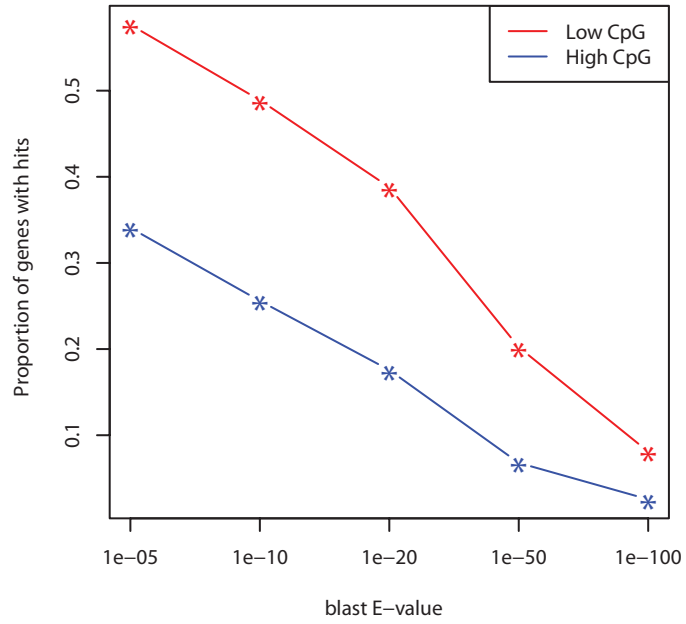

C. elegans

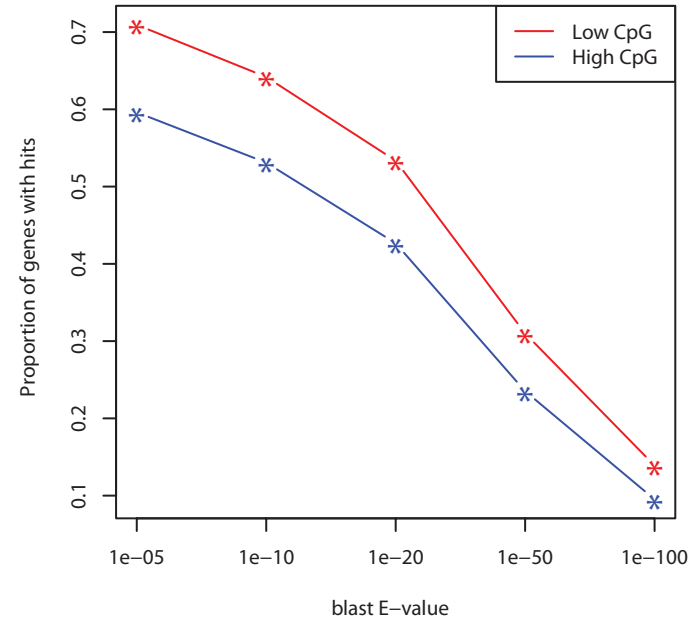

D. melanogaster

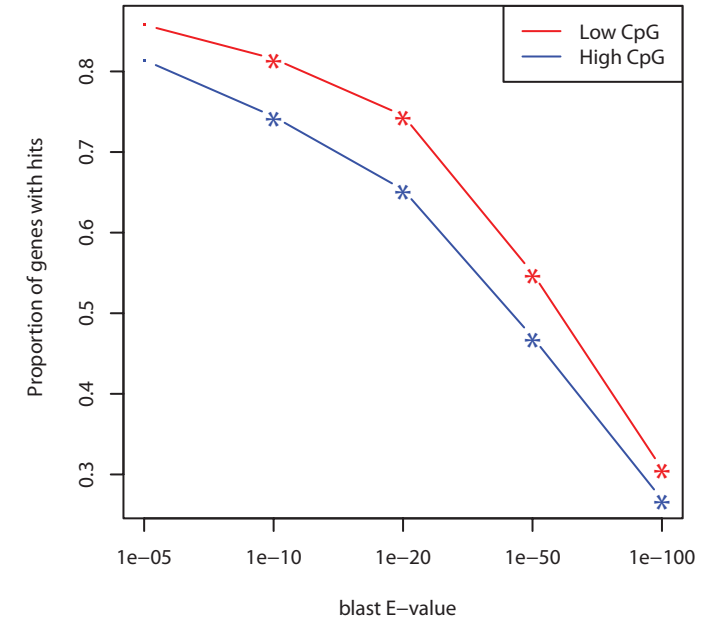

E. Coli

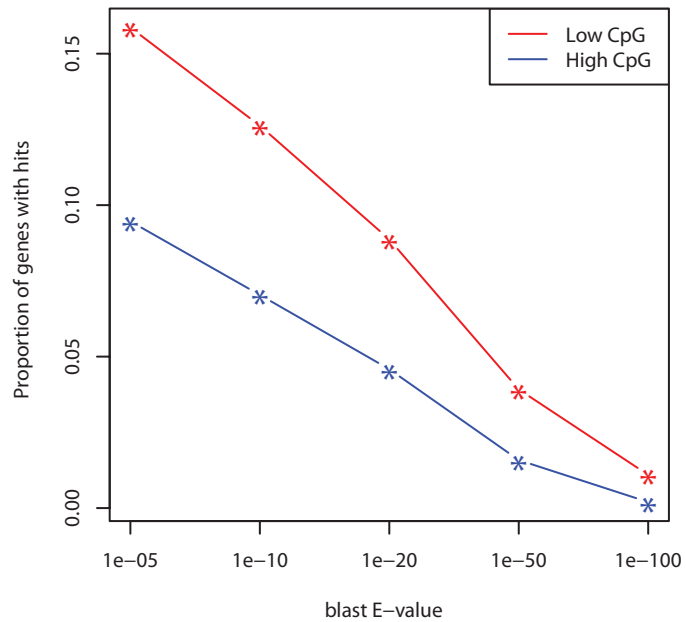

H. sapiens

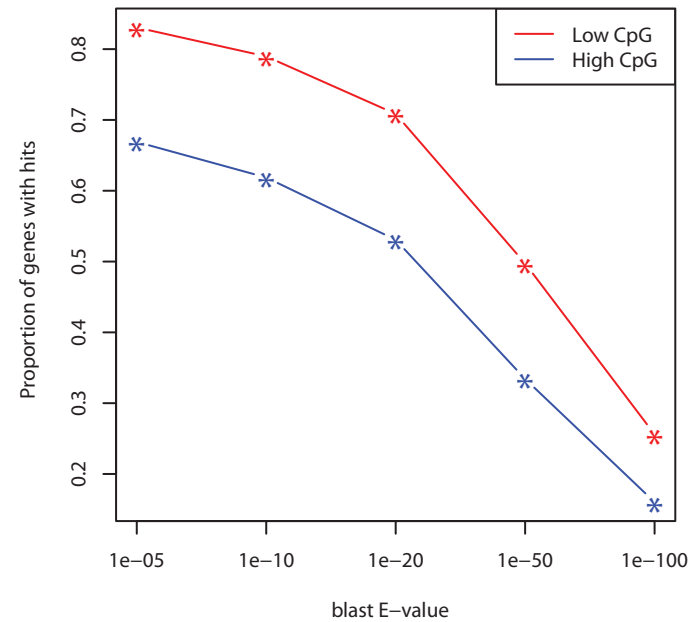

Supplement: Figure S4 — Number of high and low CpG honey bee genes with BLAST hits to different model species at various E-value thresholds. The amino acid sequences of the genes were compared. Fisher exact tests were conducted to assess whether significantly more low CpG genes have a BLAST hit than high CpG genes. Statistically significant tests at the 5% level are denoted with a star, and nonsignificant tests are shown with a dot. The details of this analysis can be found in Table S3. (0.13 MB PDF) [file pbio.1000506.s004.pdf]

A. thaliana

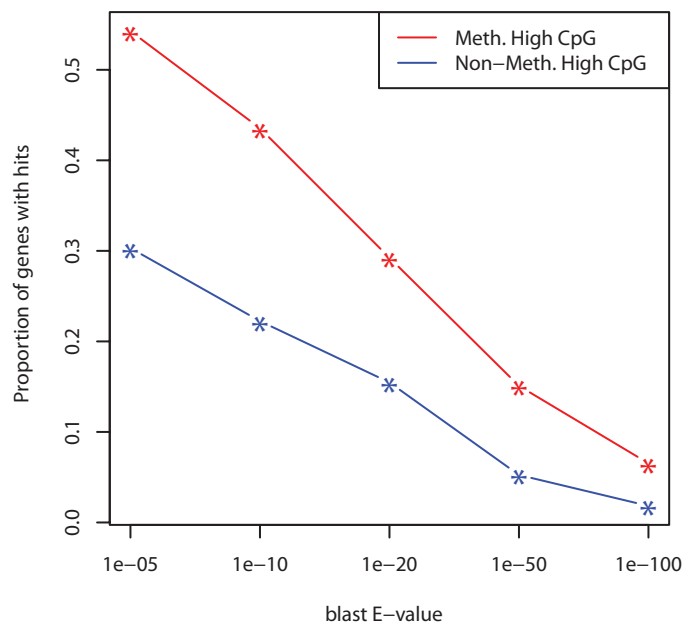

C. elegans

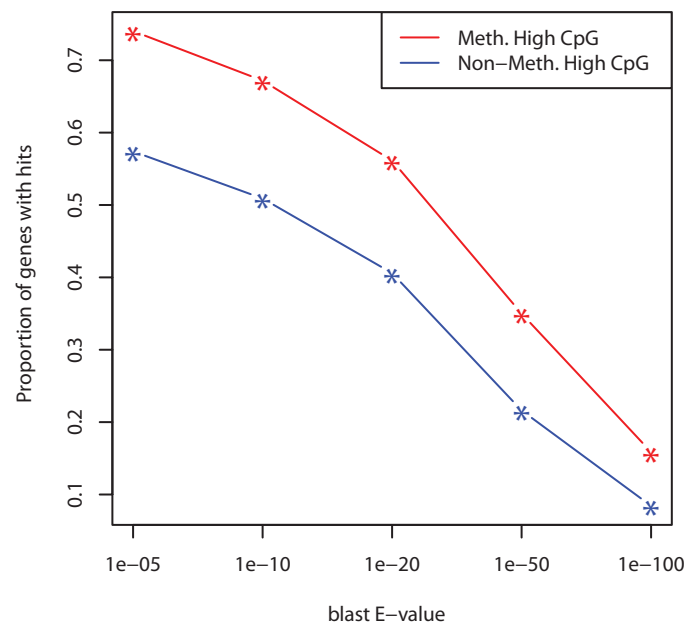

D. melanogaster

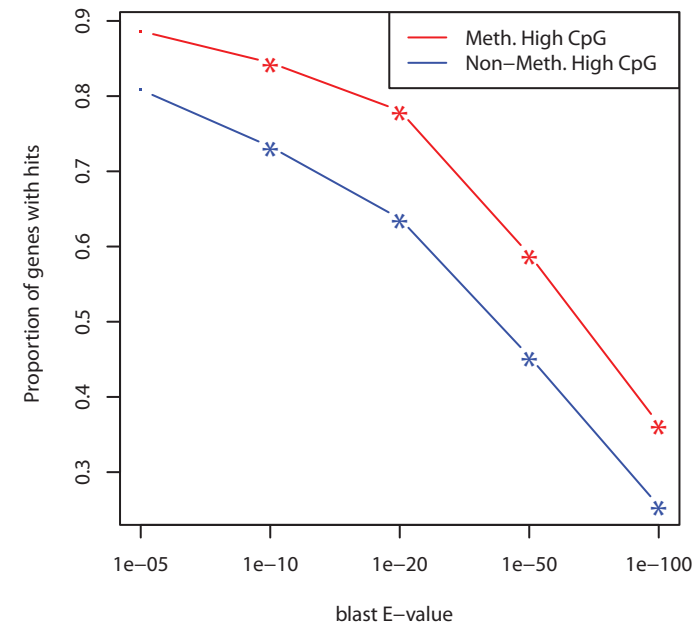

E. Coli

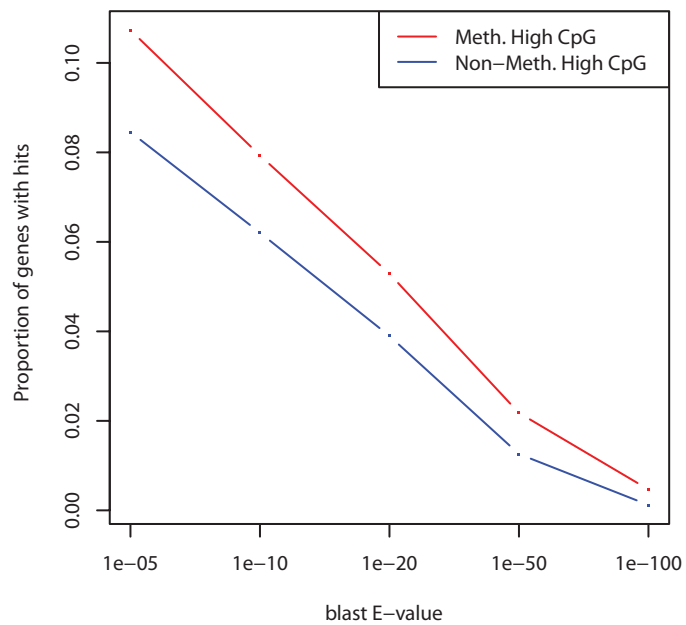

H. sapiens

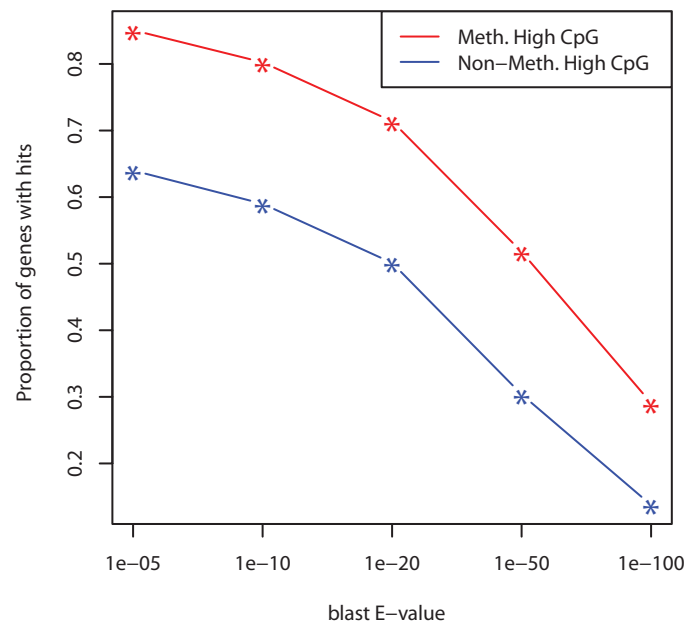

Supplement: Figure S5 — Number of high CpG methylated and non-methylated honey bee genes with BLAST hits to different model species at various E-value thresholds. The amino acid sequences of the genes were compared. Fisher exact tests were conducted to assess whether significantly more high CpG methylated genes have a BLAST hit than high CpG non-methylated genes. Statistically significant tests at the 5% level are denoted with a star, and non-significant tests are shown with a dot. The details of the analysis can be found in Table S3. (0.13 MB PDF) [file pbio.1000506.s005.pdf]

**ALU**

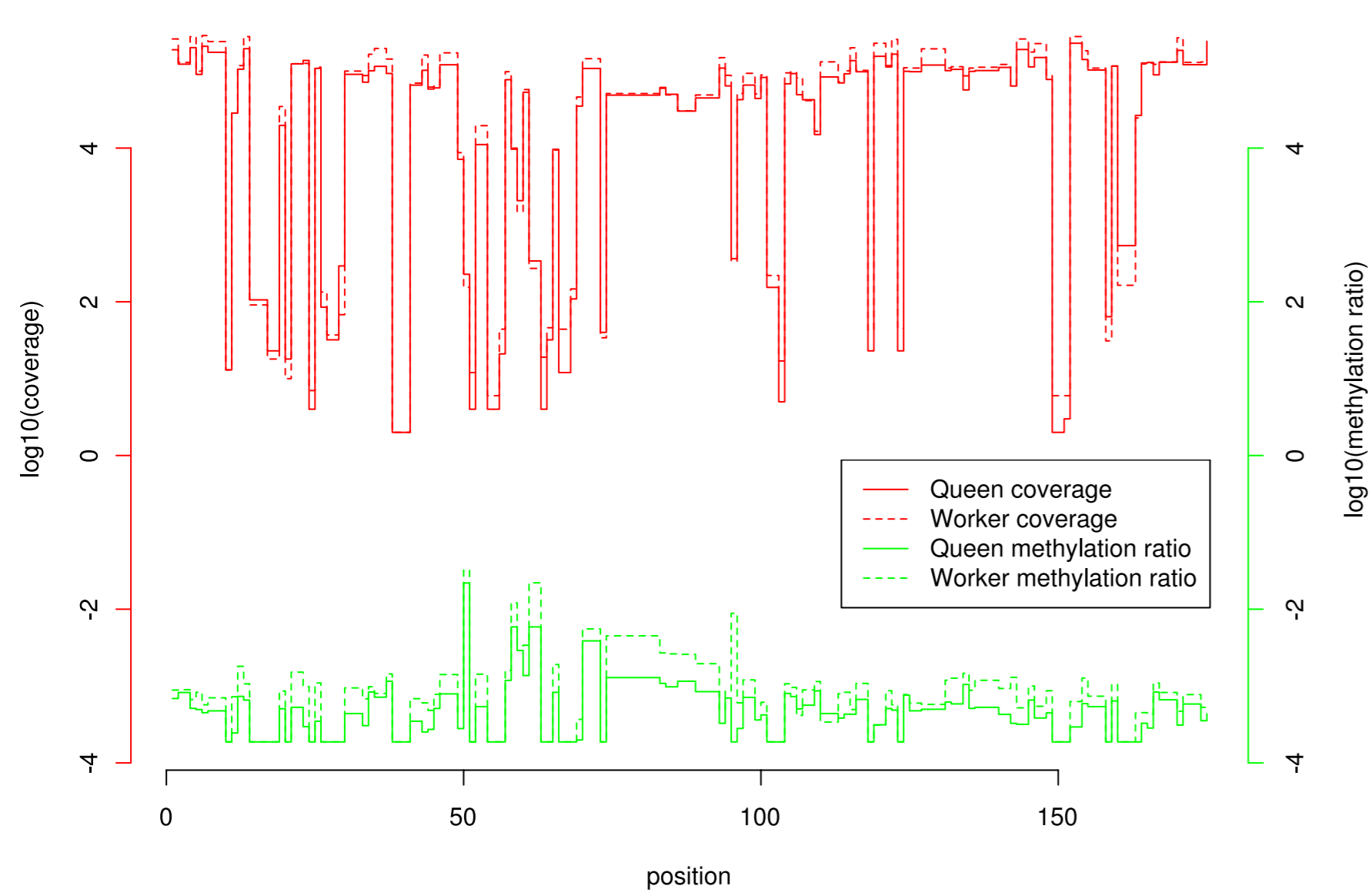

**MARINER**

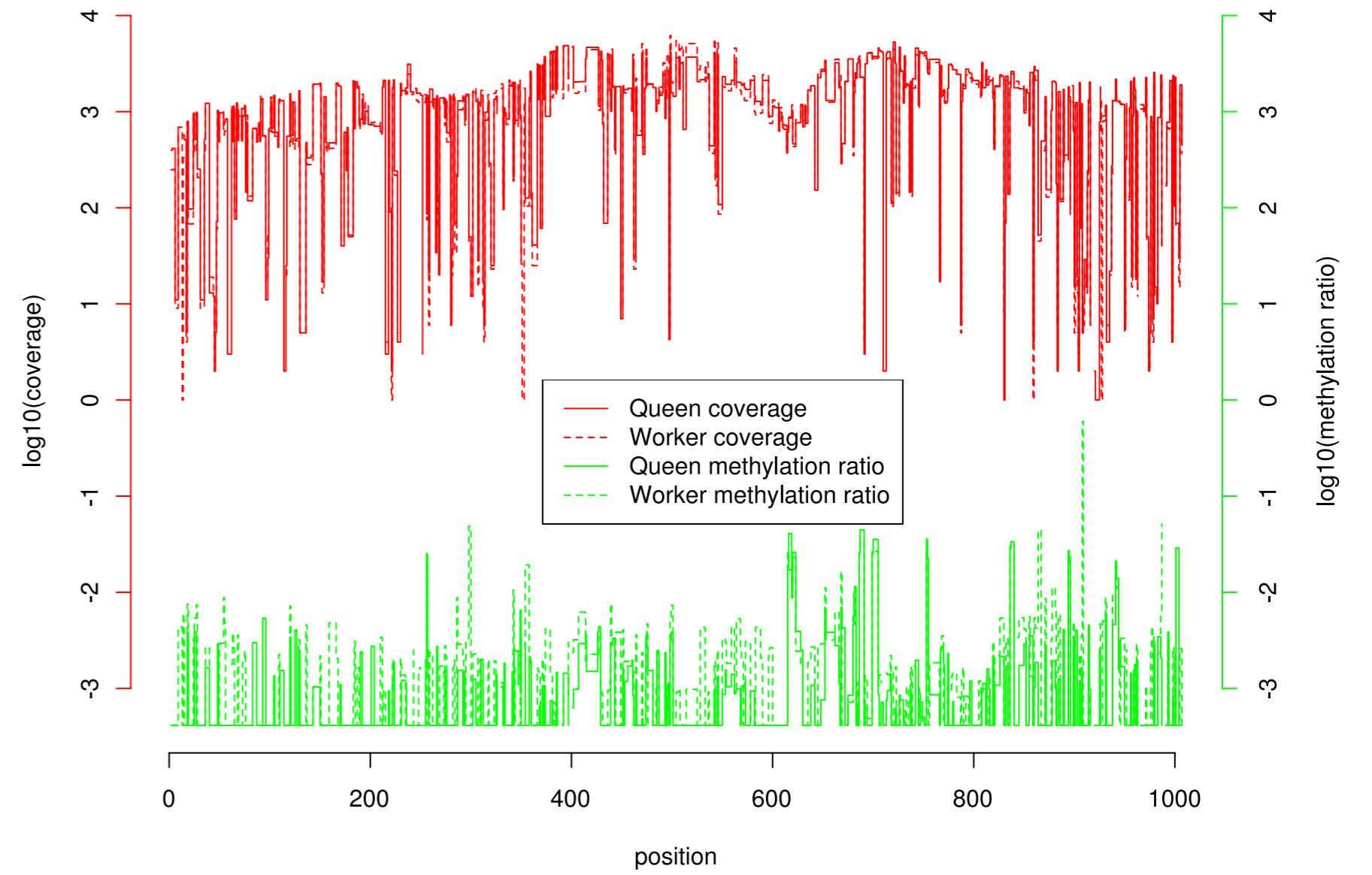

**28S**

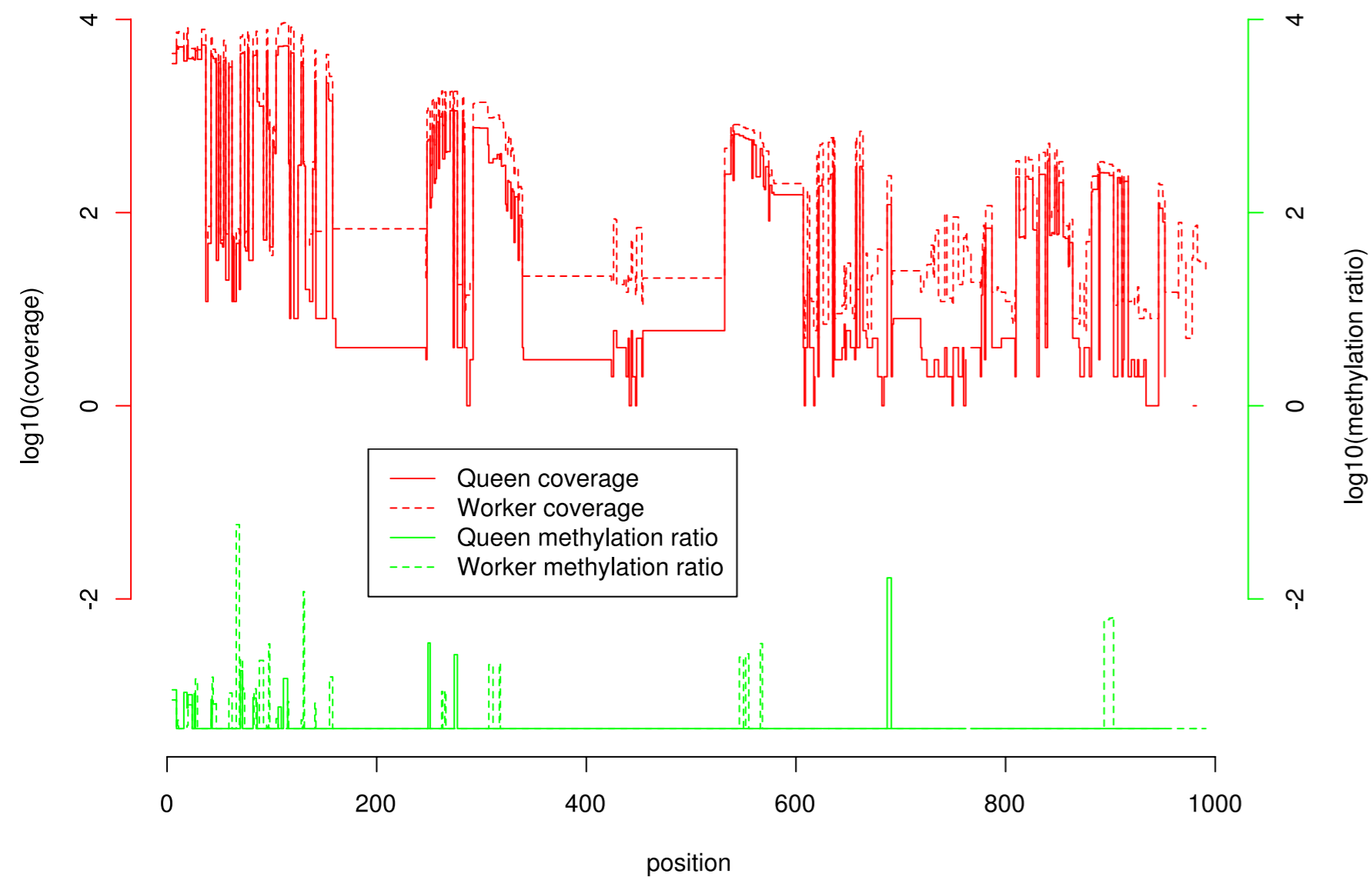

Supplement: Figure S6 — Coverage (red) and methylation ratio (green) along various kinds of repetitive elements. The methylation ratio is the proportion of the reads where a cytosine is either methylated or unconverted. The y-axes are logarithmic in base 10 (the x-axis is truncated to the nearest multiple of 50, just like the y-axis is truncated to the nearest integer). (0.63 MB PDF) [file pbio.1000506.s006.pdf]

**A****CpG autocorrelation**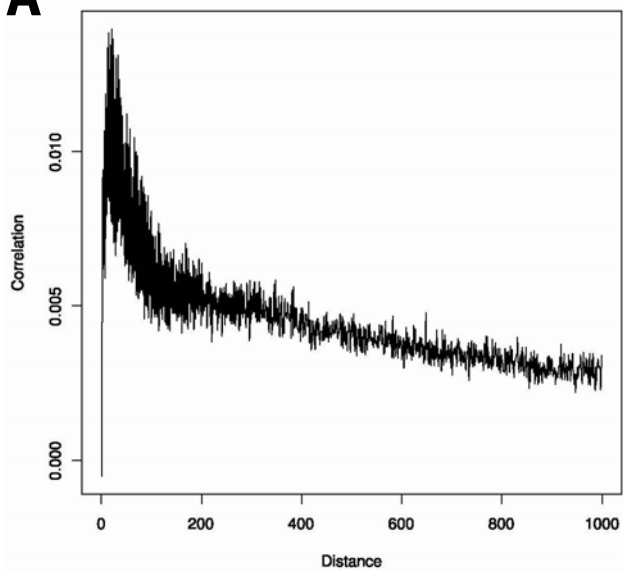**B****CpG autocorrelation**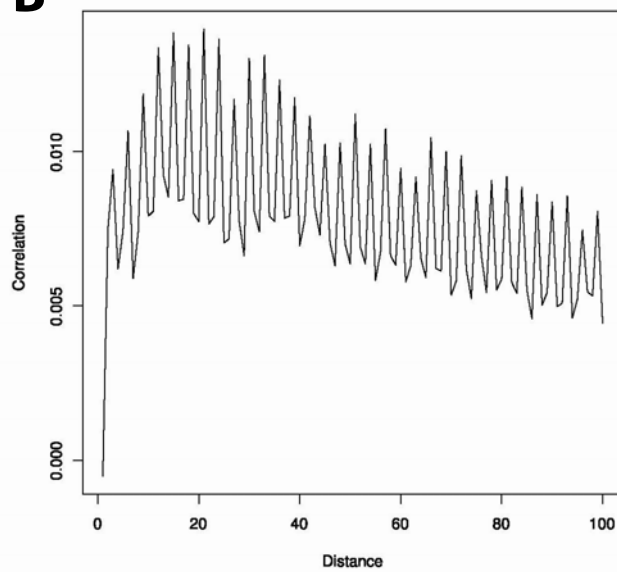**C****Fourier Transform of Autocorrelation**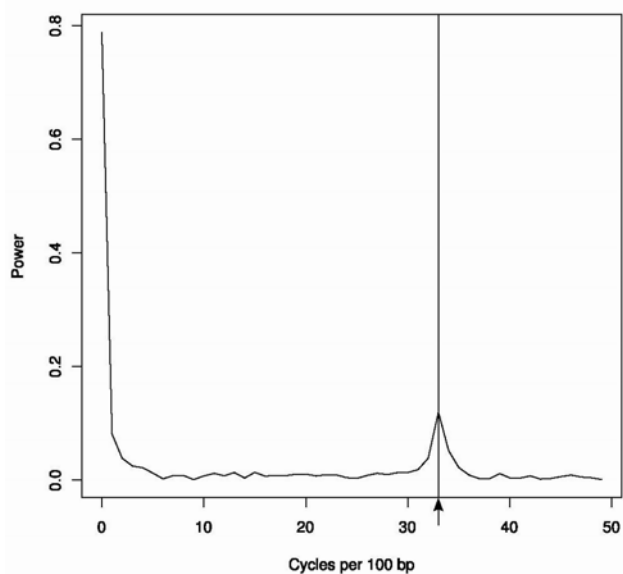**D**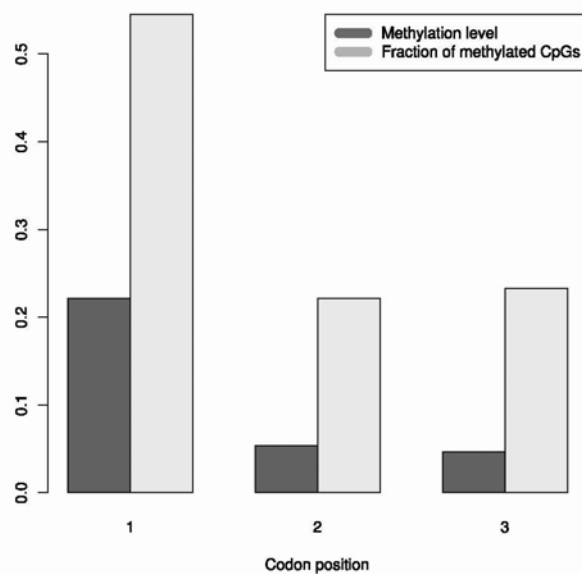

Supplement: Figure S7 — Periodicity of methylation patterns. (A) Autocorrelation of CpG methylation status over 1 kb. (B) Autocorrelation over 100 bp. Figures A and B show that the correlation of methylation status of neighboring CpGs increases sharply between 1 bp and 20 bp, then drops rapidly between 40 bp and 100 bp, and then slowly fades away. CpGs within a neighborhood of 2 bp to 100 bp are thus more likely to share the same methylation status than more distant CpGs. (C) Fourrier transform of autocorrelation showing a clear periodicity peak at 33 cycles per 100 bp (every 3 bp). (D) Distribution of codon position of mCs, and distribution of methylation level depending on the position. These two panels indicate that the distance between methylated CpGs is often a multiple of three and that the methylated cytosine corresponds most frequently to the first nucleotide of an arginine codon. (0.33 MB PDF) [file pbio.1000506.s007.pdf]

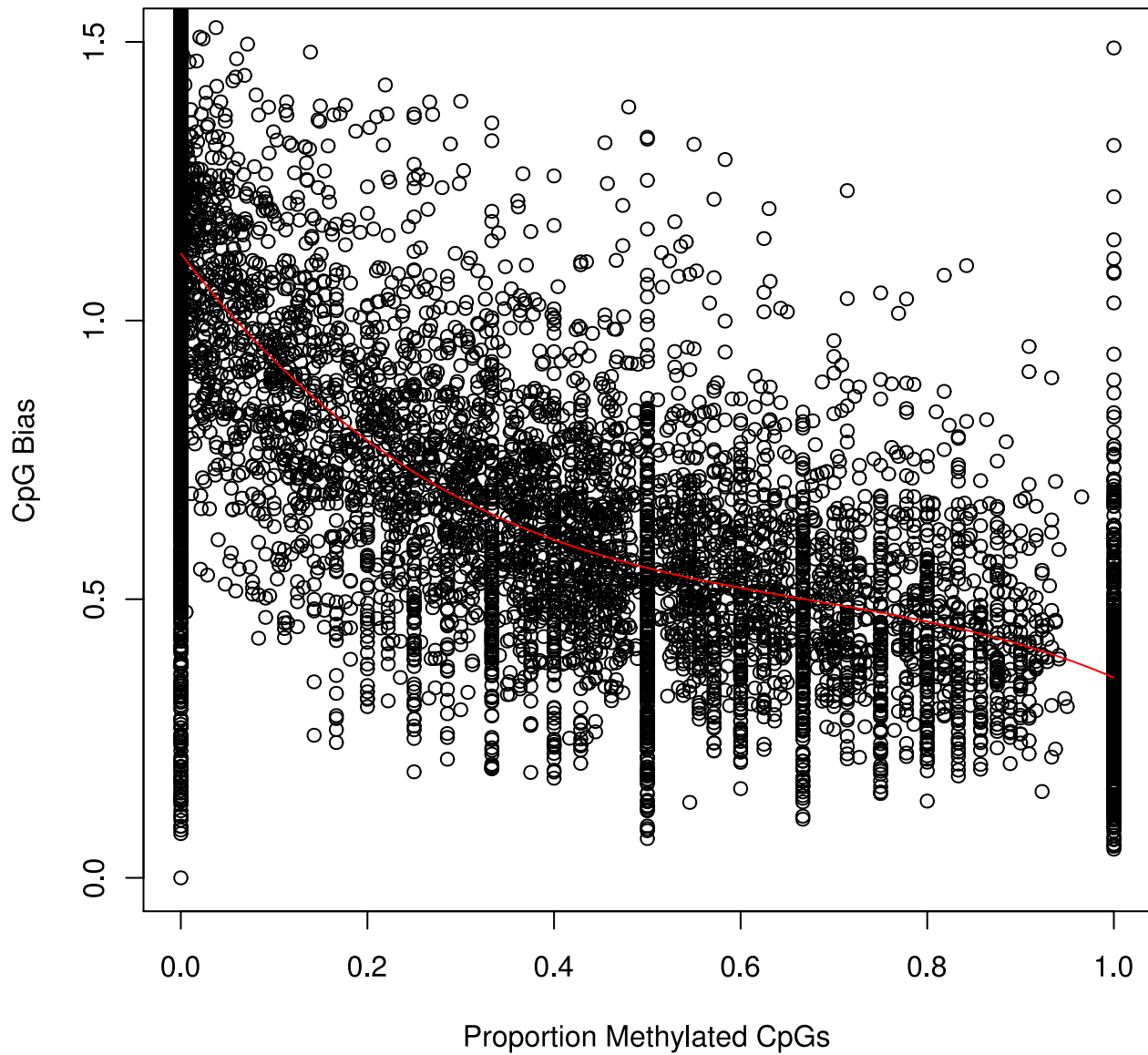

Supplement: Figure S8 — Correlation between CpG o/e and proportion of methylated CpGs. Genes with a lower CpG content tend to have a higher proportion of methylated CpGs. The red line is a polynomial regression through the points. The Akaike Information Criterion for model selection and a (monotonously decreasing) polynome of degree three was identified as the best model. (0.71 MB PDF) [file pbio.1000506.s008.pdf]

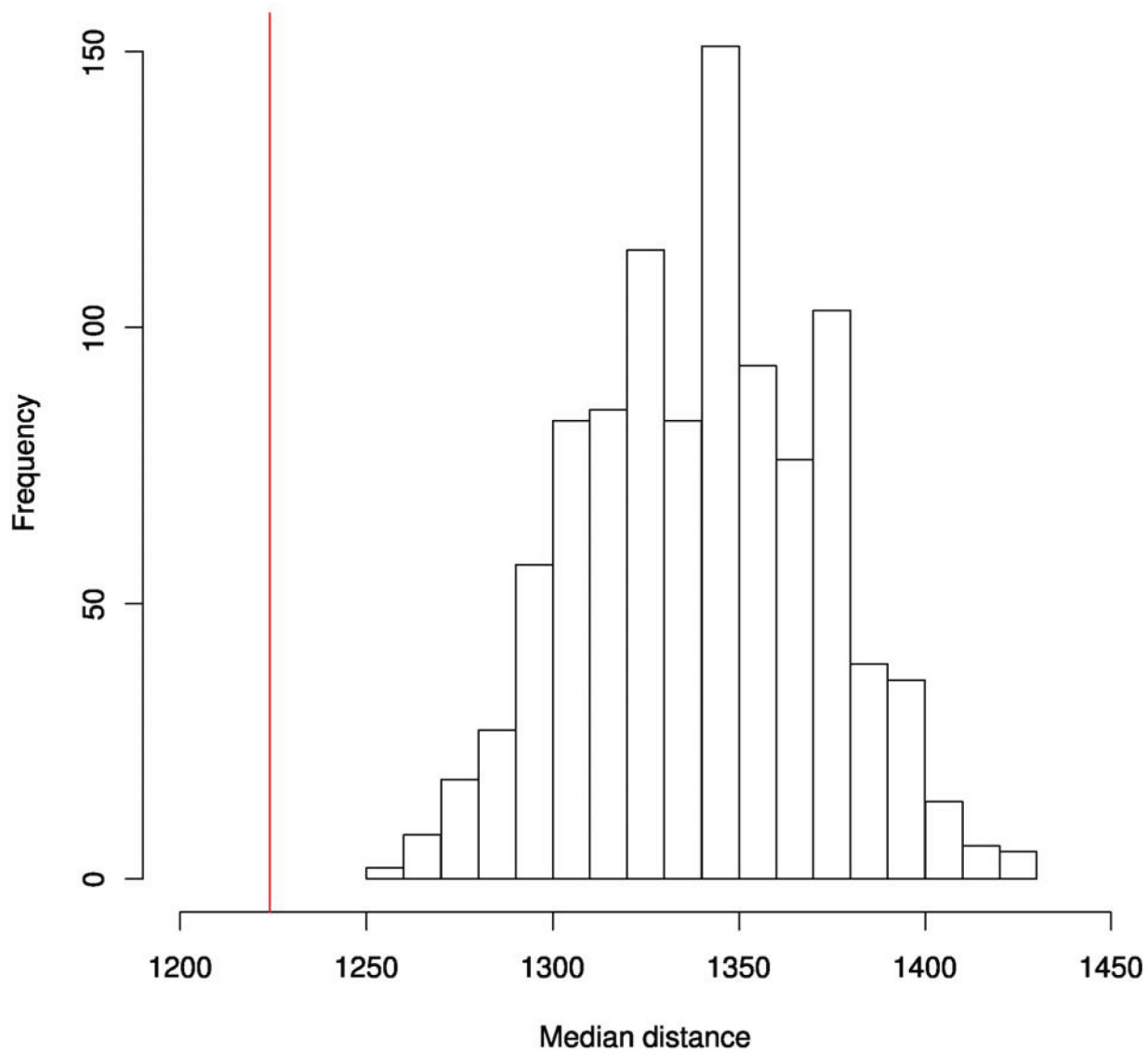

Supplement: Figure S9 — Distribution of methylated CpGs relative to splicing sites. For 169 genes, each containing a single well-defined alternative splicing event, the distance of all mCpGs to the centre of the alternatively spliced intron was computed, and the median of all these distances was calculated. A null distribution of this median distance was constructed using a randomization procedure (Manly, 2007): the methylation status of mCpGs of these genes were randomly shuffled 1,000 times, and the corresponding median distances computed. The observed value (1,224) is smaller than the smallest of the null distribution (1,259); the probability of the methylated CpGs to be as close or closer to the alternatively spliced intron as in this dataset is thus less than 0.001. (0.77 MB PDF) [file pbio.1000506.s009.pdf]

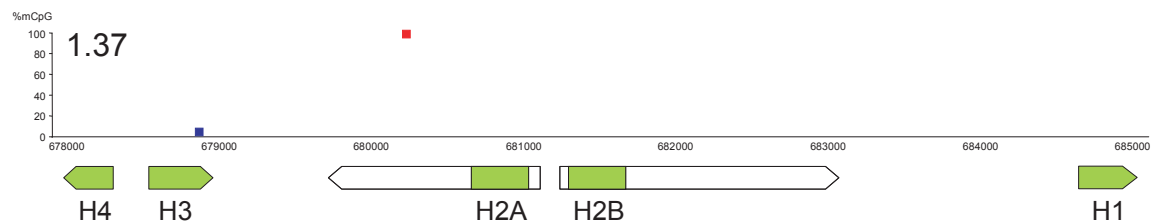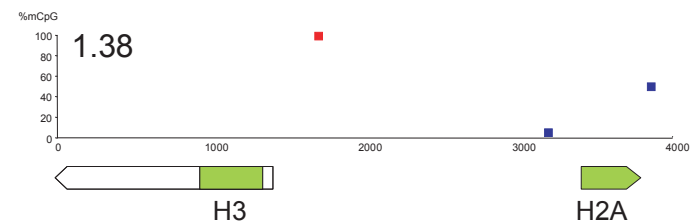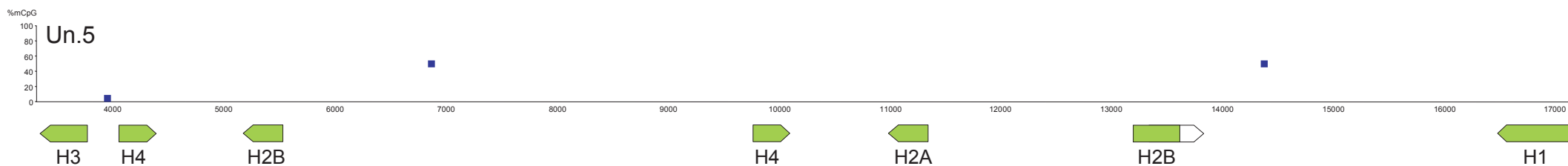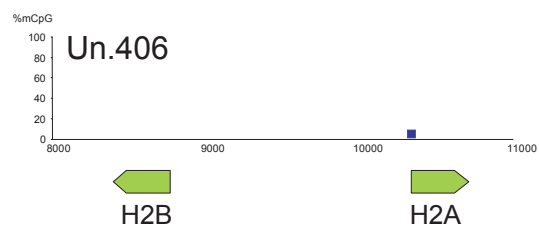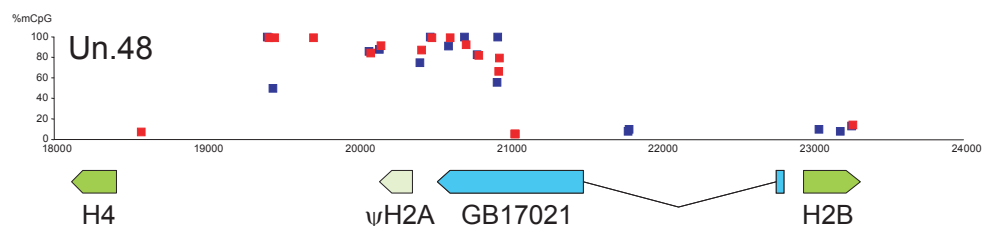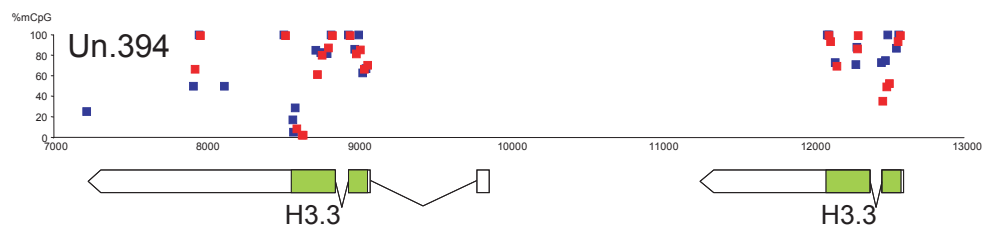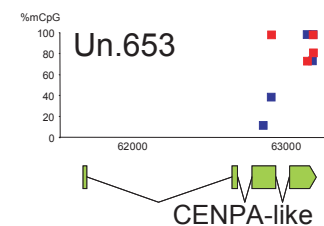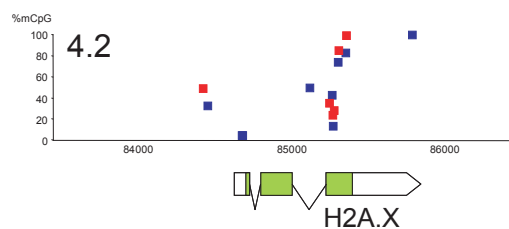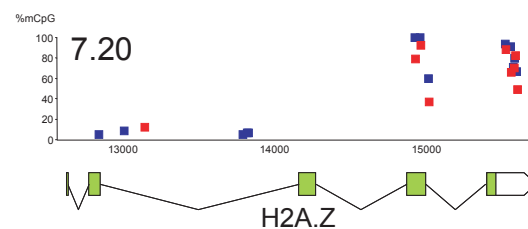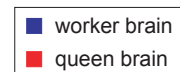

Supplement: Figure S10 — Annotation of the histone gene family in Apis mellifera showing the methylation profiles. See Table 4 for details. (0.34 MB PDF) [file pbio.1000506.s010.pdf]
