# Supplementary material for: The Honey Bee Epigenomes: Differential Methylation of Brain DNA in Queens and Workers
Source: PLoS Biol. 2010 Nov 2;8(11):e1000506. doi: 10.1371/journal.pbio.1000506 (PMC2970541; doi:10.1371/journal.pbio.1000506)
Supplement: Table S1 — Sequence conservation of methylated and non-methylated genes. (A) Number of high and low CpG Apis genes with blast hits to different species at various E-value thresholds. The amino acid sequences of the genes were compared. Fisher exact tests were conducted to assess whether significantly more low CpG genes have a blast hit than high CpG genes. (B) Number of methylated and non-methylated honey bee genes with blast hits to different model species at various E-value thresholds. The amino acid sequences of the genes were compared. Fisher exact tests were conducted to assess whether significantly more methylated genes have a blast hit than non-methylated genes. (C) Number of high CpG methylated and non-methylated honey bee genes with blast hits to different model species at various E-value thresholds. The amino acid sequences of the genes were compared. Fisher exact tests were conducted to assess whether significantly more high CpG methylated genes have a blast hit than high CpG non-methylated genes. (0.19 MB DOC) [file pbio.1000506.s011.doc]

Table S1:

A. Number of high and low CpG Apis genes with blast hits to different species at various E-value thresholds. The amino acid sequences of the genes were compared. Fisher exact tests were conducted to assess whether significantly more low CpG genes have a blast hit than high CpG genes.

| **Species** | **Blast E-value** | **Gene class** | **nb hits / nb genes** | **P-value (Fisher exact test)** |
| --- | --- | --- | --- | --- |
| **A. thaliana** | 1e-05 | Low CpG | 3841 / 6663 (57.6467%) | 8.984862e-47 |
|  |  | High CpG | 1375 / 4036 (34.06838%) |  |
|  | 1e-10 | Low CpG | 3258 / 6663 (48.89689%) | 2.164827e-59 |
|  |  | High CpG | 1033 / 4036 (25.59465%) |  |
|  | 1e-20 | Low CpG | 2585 / 6663 (38.79634%) | 1.333846e-69 |
|  |  | High CpG | 709 / 4036 (17.56690%) |  |
|  | 1e-50 | Low CpG | 1347 / 6663 (20.21612%) | 1.980717e-66 |
|  |  | High CpG | 274 / 4036 (6.7889%) |  |
|  | 1e-100 | Low CpG | 539 / 6663 (8.08945%) | 9.520864e-32 |
|  |  | High CpG | 103 / 4036 (2.552032%) |  |
| **C. elegans** | 1e-05 | Low CpG | 4727 / 6663 (70.94402%) | 5.26352e-08 |
|  |  | High CpG | 2406 / 4036 (59.61348%) |  |
|  | 1e-10 | Low CpG | 4277 / 6663 (64.1903%) | 9.64094e-09 |
|  |  | High CpG | 2143 / 4036 (53.09713%) |  |
|  | 1e-20 | Low CpG | 3555 / 6663 (53.35434%) | 2.809291e-10 |
|  |  | High CpG | 1722 / 4036 (42.66601%) |  |
|  | 1e-50 | Low CpG | 2065 / 6663 (30.99205%) | 2.491315e-10 |
|  |  | High CpG | 949 / 4036 (23.51338%) |  |
|  | 1e-100 | Low CpG | 925 / 6663 (13.88264%) | 2.073432e-09 |
|  |  | High CpG | 384 / 4036 (9.51437%) |  |
| **D. melanogaster** | 1e-05 | Low CpG | 5716 / 6663 (85.78718%) | 0.07825689 |
|  |  | High CpG | 3286 / 4036 (81.41724%) |  |
|  | 1e-10 | Low CpG | 5436 / 6663 (81.58487%) | 0.002409561 |
|  |  | High CpG | 3003 / 4036 (74.40535%) |  |
|  | 1e-20 | Low CpG | 4967 / 6663 (74.546%) | 2.582532e-05 |
|  |  | High CpG | 2637 / 4036 (65.33697%) |  |
|  | 1e-50 | Low CpG | 3664 / 6663 (54.99024%) | 6.32381e-06 |
|  |  | High CpG | 1899 / 4036 (47.05154%) |  |
|  | 1e-100 | Low CpG | 2045 / 6663 (30.69188%) | 0.001981818 |
|  |  | High CpG | 1086 / 4036 (26.90783%) |  |
| **E. coli** | 1e-05 | Low CpG | 1057 / 6663 (15.86373%) | 3.423131e-17 |
|  |  | High CpG | 382 / 4036 (9.464817%) |  |
|  | 1e-10 | Low CpG | 841 / 6663 (12.62194%) | 2.555652e-17 |
|  |  | High CpG | 284 / 4036 (7.03667%) |  |
|  | 1e-20 | Low CpG | 591 / 6663 (8.869878%) | 1.846071e-15 |
|  |  | High CpG | 185 / 4036 (4.583746%) |  |
|  | 1e-50 | Low CpG | 261 / 6663 (3.917154%) | 5.393934e-12 |
|  |  | High CpG | 64 / 4036 (1.585728%) |  |
|  | 1e-100 | Low CpG | 73 / 6663 (1.095603%) | 2.410202e-08 |
|  |  | High CpG | 8 / 4036 (0.1982161%) |  |
| **H. sapiens** | 1e-05 | Low CpG | 5534 / 6663 (83.05568%) | 2.071062e-12 |
|  |  | High CpG | 2700 / 4036 (66.89792%) |  |
|  | 1e-10 | Low CpG | 5262 / 6663 (78.97344%) | 7.093161e-15 |
|  |  | High CpG | 2497 / 4036 (61.86819%) |  |
|  | 1e-20 | Low CpG | 4721 / 6663 (70.85397%) | 9.079957e-19 |
|  |  | High CpG | 2142 / 4036 (53.07235%) |  |
|  | 1e-50 | Low CpG | 3314 / 6663 (49.73736%) | 3.435392e-26 |
|  |  | High CpG | 1349 / 4036 (33.42418%) |  |
|  | 1e-100 | Low CpG | 1705 / 6663 (25.58907%) | 2.231577e-21 |
|  |  | High CpG | 647 / 4036 (16.03072%) |  |

B. Number of methylated and non-methylated honee bee genes with blast hits to different model species at various E-value thresholds. The amino acid sequences of the genes were compared. Fisher exact tests were conducted to assess whether significantly more methylated genes have a blast hit than non-methylated genes.

| **Species** | **Blast E-value** | **Gene class** | **nb hits / nb genes** | **P-value (Fisher exact test)** |
| --- | --- | --- | --- | --- |
| **A. thaliana** | 1e-05 | Methylated | 3719 / 5854 (63.52921%) | 7.618136e-91 |
|  |  | Non-Methylated | 1380 / 4532 (30.45013%) |  |
|  | 1e-10 | Methylated | 3189 / 5854 (54.47557%) | 3.201040e-111 |
|  |  | Non-Methylated | 1016 / 4532 (22.41836%) |  |
|  | 1e-20 | Methylated | 2528 / 5854 (43.18415%) | 3.613855e-117 |
|  |  | Non-Methylated | 702 / 4532 (15.48985%) |  |
|  | 1e-50 | Methylated | 1357 / 5854 (23.18073%) | 4.846004e-116 |
|  |  | Non-Methylated | 243 / 4532 (5.361871%) |  |
|  | 1e-100 | Methylated | 560 / 5854 (9.566109%) | 4.129038e-62 |
|  |  | Non-Methylated | 80 / 4532 (1.765225%) |  |
| **C. elegans** | 1e-05 | Methylated | 4481 / 5854 (76.54595%) | 1.534413e-22 |
|  |  | Non-Methylated | 2547 / 4532 (56.20035%) |  |
|  | 1e-10 | Methylated | 4084 / 5854 (69.76426%) | 4.404675e-25 |
|  |  | Non-Methylated | 2254 / 4532 (49.73522%) |  |
|  | 1e-20 | Methylated | 3450 / 5854 (58.93406%) | 1.057533e-31 |
|  |  | Non-Methylated | 1772 / 4532 (39.09974%) |  |
|  | 1e-50 | Methylated | 2113 / 5854 (36.09498%) | 1.981263e-44 |
|  |  | Non-Methylated | 888 / 4532 (19.594%) |  |
|  | 1e-100 | Methylated | 974 / 5854 (16.63820%) | 8.161988e-38 |
|  |  | Non-Methylated | 333 / 4532 (7.34775%) |  |
| **D. melanogaster** | 1e-05 | Methylated | 5309 / 5854 (90.69013%) | 1.964013e-06 |
|  |  | Non-Methylated | 3575 / 4532 (78.8835%) |  |
|  | 1e-10 | Methylated | 5107 / 5854 (87.2395%) | 2.844341e-11 |
|  |  | Non-Methylated | 3240 / 4532 (71.49162%) |  |
|  | 1e-20 | Methylated | 4741 / 5854 (80.98736%) | 4.0287e-18 |
|  |  | Non-Methylated | 2807 / 4532 (61.93733%) |  |
|  | 1e-50 | Methylated | 3619 / 5854 (61.82098%) | 3.508273e-28 |
|  |  | Non-Methylated | 1921 / 4532 (42.38747%) |  |
|  | 1e-100 | Methylated | 2113 / 5854 (36.09498%) | 2.606176e-29 |
|  |  | Non-Methylated | 1015 / 4532 (22.39629%) |  |
| **E. coli** | 1e-05 | Methylated | 879 / 5854 (15.01537%) | 6.232392e-19 |
|  |  | Non-Methylated | 390 / 4532 (8.605472%) |  |
|  | 1e-10 | Methylated | 678 / 5854 (11.58182%) | 3.493878e-15 |
|  |  | Non-Methylated | 302 / 4532 (6.663725%) |  |
|  | 1e-20 | Methylated | 481 / 5854 (8.216604%) | 5.579879e-17 |
|  |  | Non-Methylated | 181 / 4532 (3.993822%) |  |
|  | 1e-50 | Methylated | 223 / 5854 (3.809361%) | 7.376938e-17 |
|  |  | Non-Methylated | 54 / 4532 (1.191527%) |  |
|  | 1e-100 | Methylated | 60 / 5854 (1.024940%) | 2.089921e-08 |
|  |  | Non-Methylated | 8 / 4532 (0.1765225%) |  |
| **H. sapiens** | 1e-05 | Methylated | 5224 / 5854 (89.23813%) | 8.367712e-29 |
|  |  | Non-Methylated | 2882 / 4532 (63.59223%) |  |
|  | 1e-10 | Methylated | 5020 / 5854 (85.75333%) | 1.00378e-35 |
|  |  | Non-Methylated | 2639 / 4532 (58.23036%) |  |
|  | 1e-20 | Methylated | 4583 / 5854 (78.28835%) | 6.894907e-48 |
|  |  | Non-Methylated | 2217 / 4532 (48.9188%) |  |
|  | 1e-50 | Methylated | 3366 / 5854 (57.49915%) | 8.497335e-81 |
|  |  | Non-Methylated | 1276 / 4532 (28.15534%) |  |
|  | 1e-100 | Methylated | 1804 / 5854 (30.81654%) | 9.441115e-79 |
|  |  | Non-Methylated | 545 / 4532 (12.02560%) |  |

C. Number of high CpG methylated and non-methylated honee bee genes with blast hits to different model species at various E-value thresholds. The amino acid sequences of the genes were compared. Fisher exact tests were conducted to assess whether significantly more high CpG methylated genes have a blast hit than high CpG non-methylated genes.

| **Species** | **Blast E-value** | **Gene class** | **nb hits / nb genes** | **P-value (Fisher exact test)** |
| --- | --- | --- | --- | --- |
| **A. thaliana** | 1e-05 | Meth. High CpG | 349 / 643 (54.27683%) | 4.447402e-14 |
|  |  | Non-Meth. High CpG | 1011 / 3336 (30.30576%) |  |
|  | 1e-10 | Meth. High CpG | 280 / 643 (43.54588%) | 1.706111e-15 |
|  |  | Non-Meth. High CpG | 742 / 3336 (22.24221%) |  |
|  | 1e-20 | Meth. High CpG | 188 / 643 (29.23795%) | 1.028555e-10 |
|  |  | Non-Meth. High CpG | 516 / 3336 (15.46763%) |  |
|  | 1e-50 | Meth. High CpG | 97 / 643 (15.08554%) | 7.386694e-14 |
|  |  | Non-Meth. High CpG | 175 / 3336 (5.245803%) |  |
|  | 1e-100 | Meth. High CpG | 42 / 643 (6.531882%) | 4.224944e-09 |
|  |  | Non-Meth. High CpG | 61 / 3336 (1.828537%) |  |
| **C. elegans** | 1e-05 | Meth. High CpG | 476 / 643 (74.028%) | 0.0001594714 |
|  |  | Non-Meth. High CpG | 1915 / 3336 (57.40408%) |  |
|  | 1e-10 | Meth. High CpG | 432 / 643 (67.18507%) | 6.700704e-05 |
|  |  | Non-Meth. High CpG | 1698 / 3336 (50.89928%) |  |
|  | 1e-20 | Meth. High CpG | 361 / 643 (56.14308%) | 1.275946e-05 |
|  |  | Non-Meth. High CpG | 1354 / 3336 (40.58753%) |  |
|  | 1e-50 | Meth. High CpG | 225 / 643 (34.99222%) | 7.512408e-08 |
|  |  | Non-Meth. High CpG | 721 / 3336 (21.61271%) |  |
|  | 1e-100 | Meth. High CpG | 102 / 643 (15.86314%) | 7.872634e-07 |
|  |  | Non-Meth. High CpG | 281 / 3336 (8.423261%) |  |
| **D. melanogaster** | 1e-05 | Meth. High CpG | 570 / 643 (88.64697%) | 0.1459081 |
|  |  | Non-Meth. High CpG | 2697 / 3336 (80.84532%) |  |
|  | 1e-10 | Meth. High CpG | 543 / 643 (84.4479%) | 0.02851028 |
|  |  | Non-Meth. High CpG | 2445 / 3336 (73.29137%) |  |
|  | 1e-20 | Meth. High CpG | 502 / 643 (78.07154%) | 0.002241826 |
|  |  | Non-Meth. High CpG | 2126 / 3336 (63.72902%) |  |
|  | 1e-50 | Meth. High CpG | 379 / 643 (58.94246%) | 0.0003059133 |
|  |  | Non-Meth. High CpG | 1514 / 3336 (45.38369%) |  |
|  | 1e-100 | Meth. High CpG | 234 / 643 (36.39191%) | 4.513479e-05 |
|  |  | Non-Meth. High CpG | 851 / 3336 (25.50959%) |  |
| **E. coli** | 1e-05 | Meth. High CpG | 69 / 643 (10.73095%) | 0.09814891 |
|  |  | Non-Meth. High CpG | 282 / 3336 (8.453237%) |  |
|  | 1e-10 | Meth. High CpG | 51 / 643 (7.93157%) | 0.1396222 |
|  |  | Non-Meth. High CpG | 207 / 3336 (6.205036%) |  |
|  | 1e-20 | Meth. High CpG | 34 / 643 (5.287714%) | 0.1308037 |
|  |  | Non-Meth. High CpG | 130 / 3336 (3.896882%) |  |
|  | 1e-50 | Meth. High CpG | 14 / 643 (2.177294%) | 0.09778345 |
|  |  | Non-Meth. High CpG | 42 / 3336 (1.258993%) |  |
|  | 1e-100 | Meth. High CpG | 3 / 643 (0.466563%) | 0.08923827 |
|  |  | Non-Meth. High CpG | 4 / 3336 (0.1199041%) |  |
| **H. sapiens** | 1e-05 | Meth. High CpG | 547 / 643 (85.06998%) | 1.061170e-05 |
|  |  | Non-Meth. High CpG | 2135 / 3336 (63.9988%) |  |
|  | 1e-10 | Meth. High CpG | 516 / 643 (80.24883%) | 3.020943e-06 |
|  |  | Non-Meth. High CpG | 1968 / 3336 (58.9928%) |  |
|  | 1e-20 | Meth. High CpG | 459 / 643 (71.38414%) | 2.758004e-07 |
|  |  | Non-Meth. High CpG | 1672 / 3336 (50.1199%) |  |
|  | 1e-50 | Meth. High CpG | 333 / 643 (51.78849%) | 8.314482e-12 |
|  |  | Non-Meth. High CpG | 1013 / 3336 (30.36571%) |  |
|  | 1e-100 | Meth. High CpG | 187 / 643 (29.08243%) | 1.097424e-13 |
|  |  | Non-Meth. High CpG | 459 / 3336 (13.75899%) |  |
