# Supplementary material for: The Honey Bee Epigenomes: Differential Methylation of Brain DNA in Queens and Workers
Source: PLoS Biol. 2010 Nov 2;8(11):e1000506. doi: 10.1371/journal.pbio.1000506 (PMC2970541; doi:10.1371/journal.pbio.1000506)
Supplement: Table S2 — Differentially methylated genes in queens and worker brains. A generalized linear model of the binomial family was used to identify genes that are differentially methylated between castes. The methylation level of each gene was modeled as a function of the caste and of each of its CpG dinucleotides. In the table, “Caste” indicates whether the caste is a statistically significant factor explaining differences in methylation levels, “CpG” represents the different dinucleotides of that gene, and “Caste * CpG,” the interaction factor, indicates whether the CpG dinucleotides behave differently between castes. GB numbers refer to the proteins at BeeBase: genomes.arc.georgetown.edu/drupal. Genes were ranked into 10 bins based on their expression levels from low (1) to high (10). No value in the relative expression column indicates those genes that are not represented on the microarray. Based on microarray data from Foret et al. [10]. (1.13 MB DOC) [file pbio.1000506.s012.doc]

**TABLE S2**

Differentially methylated genes in queens and worker brains

A generalized linear model of the binomial family was used to identify genes that are differentially methylated between castes. The methylation level of each gene was modelled as a function of the caste and of each of its CpG dinucleotides. In the table, “Caste” indicates whether the caste is a statistically significant factor explaining differences in methylation levels, “CpG” represents the different dinucleotides of that gene, and “Caste * CpG”, the interaction factor, indicates whether the CpG dinucleotides behave differently between castes. *GB numbers refer to the proteins at BeeBase: genomes.arc.georgetown.edu/drupal

** Genes were ranked into 10 bins based on their expression levels from low (1) to high (10). No value in the relative expression column indicates those genes that are not represented on the microarray. Based on microarray data from Foret et al^10^ .

|  |  |  |  |  | **Relative expression** | | | | | |  |
| --- | --- | --- | --- | --- | --- | --- | --- | --- | --- | --- | --- |
| **GLEAN** | **# CpGs coverage >= 2** | **Caste** | **CpG** | **Caste * CpG** | **Antenna** | **Brain** | **HPG** | **Larva** | **Ovary** | **Thorax** | **Annotations** |
| GB18602 | 30 | 0.00% | 0.00% | 0.00% | 1 | 1 | 1 | 1 | 1 | 1 | YhhN The members of this family are similar to a transmembrane protein yhhN expressed by E. coli. |
| GB18303 | 13 | 0.00% | 0.90% | 0.05% | 1 | 1 | 1 | 1 | 1 | 1 | Contains TBC domain - GTPase activator proteins of Rab-like small GTPases. |
| GB13368 | 9 | 0.00% | 0.42% | 0.77% | 2 | 1 | 2 | 10 | 1 | 3 | NAD-binding lambda crystallin-like protein, 3-hydroxyacyl-CoA dehydrogenase, NADB Rossman |
| GB13215 | 34 | 0.00% | 0.00% | 0.01% | 1 | 1 | 1 | 1 | 1 | 1 | Glycine cleavage system P-protein, |
| GB15588 | 9 | 0.00% | 0.00% | 0.02% | 1 | 1 | 1 | 1 | 1 | 1 | Low-density lipoprotein receptor domain class A - Cysteine-rich repeat in the low-density lipoprotein (LDL) receptor |
| GB15132 | 24 | 0.00% | 0.00% | 0.00% | 1 | 1 | 1 | 1 | 1 | 1 | Tap42 domain that negatively regulates the TOR signaling pathway. |
| GB12560 | 12 | 0.00% | 0.16% | 0.06% | 1 | 1 | 1 | 1 | 9 | 1 | oo18 RNA-binding protein |
| GB12560 | 12 | 0.00% | 0.16% | 0.06% | 1 | 1 | 1 | 1 | 9 | 1 | oo18 RNA-binding protein isoform A |
| GB11648 | 13 | 0.00% | 0.00% | 0.00% |  |  |  |  |  |  | catalase |
| GB30227 | 13 | 0.00% | 0.00% | 0.00% | 1 | 1 | 1 | 1 | 2 | 1 | catalase (Cat), isoform-B |
| GB19645 | 12 | 0.00% | 0.98% | 0.86% | 1 | 1 | 1 | 1 | 1 | 1 | Phosphodiesterase 6 |
| GB12929 | 39 | 0.00% | 0.00% | 0.00% | 1 | 1 | 1 | 1 | 1 | 1 | paralytic |
| GB11421 | 31 | 0.00% | 0.00% | 0.00% | 1 | 1 | 1 | 1 | 1 | 1 | similarity at 5' end to tight junction associated protein |
| GB19503 | 33 | 0.00% | 0.00% | 0.01% | 1 | 1 | 1 | 1 | 1 | 1 | heat shock protein 8 |
| GB13740 | 24 | 0.00% | 0.00% | 0.09% | 1 | 1 | 1 | 1 | 1 | 1 | dysfusion |
| GB10394 | 8 | 0.00% | 0.06% | 0.42% | 1 | 1 | 1 | 1 | 1 | 1 | TNF-receptor-associated factor 1 |
| GB19152 | 10 | 0.00% | 0.00% | 0.05% | 1 | 1 | 1 | 1 | 1 | 1 | CG32227-PA (LOC551433), partial mRNA |
| GB11485 | 14 | 0.00% | 0.00% | 0.00% | 1 | 1 | 1 | 2 | 3 | 1 | CG5651-PA, isoform A, transcript variant 1 (LOC551235), mRNA |
| GB16628 | 9 | 0.00% | 0.00% | 0.07% | 10 | 6 | 8 | 10 | 10 | 10 | Ribosomal protein L6 CG11522-PB, isoform B (LOC409479), mRNA |
| GB18610 | 53 | 0.00% | 0.00% | 0.00% | 2 | 3 | 1 | 2 | 7 | 1 | RNA binding motif protein 25 (LOC408705), mRNA |
| GB10198 | 11 | 0.00% | 0.03% | 0.50% | 3 | 2 | 1 | 1 | 3 | 1 | CG5850-PA (LOC552432), mRNA (transmembrane protein in Mus) |
| GB12783 | 10 | 0.00% | 0.22% | 0.04% | 1 | 1 | 1 | 1 | 1 | 1 | zf-MYND at 3' end |
| GB16555 | 23 | 0.00% | 0.00% | 0.11% | 1 | 2 | 1 | 1 | 1 | 1 | Phospholipase C at 21C CG4574-PC, isoform C (LOC408791), mRNA |
| GB17864 | 23 | 0.00% | 0.00% | 0.00% |  |  |  |  |  |  | AcetylCoA_hydro, |
| GB20011 | 6 | 0.00% | 0.02% | 0.44% | 1 | 2 | 2 | 1 | 3 | 1 | CG10103-PA (LOC727141), mRNA ??? |
| GB14825 | 46 | 0.00% | 0.00% | 0.00% | 1 | 1 | 1 | 1 | 1 | 1 | CG8516-PA (LOC410987), mRNA (envelope glycoprotein ?) |
| GB17442 | 18 | 0.00% | 0.00% | 0.01% | 1 | 1 | 1 | 1 | 1 | 1 | Van Gogh CG8075-PA (LOC551508), mRNA |
| GB13192 | 42 | 0.00% | 0.00% | 0.01% |  |  |  |  |  |  | kuzbanian Zinc-dependent metalloprotease |
| GB16408 | 15 | 0.00% | 0.98% | 1.00% | 2 | 3 | 1 | 1 | 1 | 1 | no extended memory CG8772-PA, isoform A, transcript variant 1 |
| GB13866 | 7 | 0.00% | 0.56% | 0.05% | 1 | 1 | 1 | 1 | 1 | 1 | axonemal dynein light chain (LOC551085), mRNA |
| GB13464 | 42 | 0.00% | 0.00% | 0.00% | 1 | 1 | 1 | 1 | 1 | 1 | RhoGAP93B CG3421-PA (LOC409268), mRNA |
| GB19722 | 15 | 0.00% | 0.00% | 0.06% | 1 | 1 | 1 | 1 | 1 | 1 | G protein beta subunit-like (LOC409725), mRNA |
| GB15860 | 9 | 0.00% | 0.00% | 0.00% | 1 | 1 | 1 | 1 | 1 | 1 | Peroxidasin CG12002-PA, isoform A (LOC413025), mRNA |
| GB15458 | 9 | 0.00% | 0.00% | 0.86% | 1 | 1 | 1 | 1 | 1 | 1 | CG6405-PA (LOC411721), mRNA |
| GB18549 | 21 | 0.00% | 0.00% | 0.03% | 1 | 1 | 1 | 1 | 1 | 1 | CG8773-PA, transcript variant 1 (LOC551518), partial mRNA |
| GB13626 | 8 | 0.00% | 0.99% | 0.67% | 1 | 1 | 1 | 4 | 1 | 1 | CG6206-PB, isoform B (LOC552249), mRNA |
| GB19241 | 20 | 0.00% | 0.00% | 0.03% | 1 | 1 | 1 | 1 | 2 | 1 | CG3880-PA (LOC413345), mRNA |
| GB19430 | 4 | 0.00% | 0.01% | 0.23% | 1 | 1 | 1 | 1 | 1 | 1 | hypothetical LOC551564 (LOC551564), mRNA ???? |
| GB19860 | 19 | 0.00% | 0.00% | 0.17% | 1 | 1 | 1 | 1 | 1 | 1 | Heat shock protein cognate 5 CG8542-PA (LOC408605), mRNA |
| GB16513 | 11 | 0.00% | 0.03% | 0.17% | 1 | 1 | 1 | 1 | 1 | 1 | DEAD BOX helicase RecQ protein |
| GB19598 | 20 | 0.00% | 0.00% | 0.00% | 1 | 1 | 1 | 1 | 1 | 1 | centrosomal protein 4 (LOC725707), mRNA |
| GB11875 | 6 | 0.00% | 1.00% | 1.00% | 1 | 1 | 1 | 1 | 1 | 1 | a relative of Apis protein present only in louse, aphid and thick |
| GB18943 | 8 | 0.00% | 0.00% | 0.09% | 1 | 1 | 1 | 1 | 1 | 1 | CG14464-PA.3 (LOC725871), mRNA |
| GB19370 | 24 | 0.00% | 0.00% | 0.02% | 1 | 1 | 1 | 1 | 1 | 1 | Splicing factor 1 CG5836-PA (LOC411122), mRNA |
| GB11638 | 4 | 0.00% | 0.87% | 0.64% | 1 | 2 | 1 | 1 | 1 | 1 | Protein anon-35F/36A (LOC551415), mRNA |
| GB15919 | 18 | 0.00% | 0.00% | 0.00% | 1 | 1 | 1 | 1 | 1 | 1 | CG9977-PA (LOC551762), mRNA |
| GB18804 | 30 | 0.00% | 0.00% | 0.00% | 1 | 1 | 1 | 1 | 1 | 1 | DNA polymerase type-B zeta subfamily catalytic domain |
| GB10706 | 40 | 0.00% | 0.00% | 0.00% | 1 | 1 | 1 | 1 | 3 | 1 | 5-3 exoribonuclease 1, transcript variant 1 |
| GB19078 | 5 | 0.00% | 0.82% | 1.00% | 1 | 1 | 1 | 5 | 2 | 1 | phosphoserine aminotransferase 1 isoform |
| GB16058 | 29 | 0.00% | 0.95% | 0.00% | 1 | 1 | 1 | 1 | 1 | 1 | Disk large |
| GB14251 | 2 | 0.00% | 0.84% | 1.00% | 1 | 1 | 1 | 1 | 1 | 1 | Short 'novel' peptide unknown function |
| GB13273 | 4 | 0.00% | 0.73% | 0.29% |  |  |  |  |  |  | similarity to gliotactin |
| GB16952 | 16 | 0.00% | 0.00% | 0.01% | 5 | 2 | 10 | 9 | 4 | 5 | 60S ribosomal protein L15 (LOC413137), mRNA |
| GB18437 | 8 | 0.00% | 0.24% | 0.04% | 1 | 1 | 1 | 1 | 1 | 1 | CG30429-PA (higher similarity to mammals than to insects, MORN -repeat containig) |
| GB19121 | 41 | 0.00% | 0.00% | 0.00% | 1 | 1 | 1 | 1 | 1 | 1 | Ubiquitin carboxyl-terminal hydrolase 4 (Ubiquitin thioesterase 4) (Ubiquitin-specific-processing protease 4) |
| GB17536 | 21 | 0.00% | 0.00% | 0.10% | 1 | 1 | 1 | 1 | 1 | 1 | Alternative splicing regulator |
| GB19909 | 12 | 0.00% | 0.00% | 0.00% | 1 | 1 | 1 | 1 | 1 | 1 | sec10 CG6159-PA |
| GB19062 | 18 | 0.00% | 0.00% | 0.04% | 1 | 1 | 1 | 1 | 1 | 1 | bonus CG5206-PA |
| GB18207 | 31 | 0.00% | 0.00% | 0.00% | 1 | 1 | 1 | 1 | 1 | 1 | Protocadherin-like wing polarity protein stan precursor (Protein starry night) (flamingo) |
| GB13911 | 22 | 0.00% | 0.00% | 0.79% |  |  |  |  |  |  | Possible chimera (2 genes in NCBI) |
| GB30309 | 22 | 0.00% | 0.00% | 0.79% | 1 | 1 | 1 | 1 | 3 | 1 | zinc finger protein 658 (LOC726368), mRNA |
| GB19334 | 41 | 0.00% | 0.00% | 0.01% | 1 | 1 | 1 | 1 | 1 | 1 | glutamate receptor, ionotropic, N-methyl-D-aspartate 3A ( |
| GB13391 | 11 | 0.00% | 0.30% | 0.78% | 1 | 1 | 1 | 1 | 1 | 1 | beat-IIIa highly conserved in insects unknown function |
| GB18959 | 9 | 0.00% | 0.00% | 0.01% | 1 | 1 | 1 | 1 | 3 | 1 | Mediator complex subunit 17 CG7957-PA ( |
| GB17165 | 14 | 0.00% | 0.00% | 0.16% | 1 | 3 | 1 | 2 | 3 | 5 | Protein stoned-B (StonedB) (Stn-B) |
| GB18324 | 37 | 0.00% | 0.00% | 0.00% | 1 | 1 | 1 | 1 | 1 | 1 | galectin2 |
| GB13821 | 6 | 0.00% | 0.99% | 0.84% | 1 | 1 | 1 | 2 | 1 | 1 | CG3609-PA |
| GB13821 | 6 | 0.00% | 0.99% | 0.84% | 1 | 1 | 1 | 2 | 1 | 1 | CG3609-PA |
| GB13821 | 6 | 0.00% | 0.99% | 0.84% | 1 | 1 | 1 | 2 | 1 | 1 | CG3609-PA |
| GB18941 | 53 | 0.00% | 0.00% | 0.00% | 1 | 1 | 1 | 1 | 1 | 1 | CG10188-PB, isoform B ( |
| GB15382 | 15 | 0.00% | 0.02% | 0.06% | 1 | 1 | 1 | 1 | 1 | 1 | CG15216-PA (LOC408731), mRNA |
| GB19935 | 11 | 0.00% | 0.00% | 0.74% | 1 | 1 | 1 | 2 | 1 | 1 | CG3609-PA (LOC552024), mRNA |
| GB30012 | 14 | 0.00% | 0.00% | 0.01% | 1 | 1 | 1 | 1 | 1 | 1 | Trypsin-like serine protease; |
| GB11424 | 7 | 0.00% | 0.92% | 0.74% | 1 | 1 | 1 | 1 | 1 | 1 | Short peptide very simuialr to fragemnt of hepatocyte nuclear factor 4, |
| GB16944 | 14 | 0.00% | 0.00% | 0.00% | 1 | 1 | 1 | 1 | 1 | 1 | CG8379-PA, isoform A, transcript variant 1 |
| GB18802 | 39 | 0.00% | 0.00% | 0.00% | 1 | 1 | 1 | 1 | 1 | 1 | Ataxin-2 CG5166-PB, isoform B |
| GB11158 | 40 | 0.00% | 0.00% | 0.00% | 1 | 1 | 1 | 1 | 1 | 1 | Enhancer of Polycomb CG7776-PA, isoform A |
| GB17851 | 27 | 0.00% | 0.00% | 0.02% | 1 | 1 | 1 | 1 | 1 | 1 | CG1504-PA (LOC726572), mRNA |
| GB16690 | 5 | 0.00% | 1.00% | 1.00% | 1 | 1 | 1 | 1 | 1 | 1 | zinc finger protein 91 (LOC724604), mRNA |
| GB12720 | 21 | 0.00% | 0.00% | 0.04% | 1 | 1 | 1 | 1 | 1 | 1 | CG6192-PA (LOC726273), mRNA |
| GB10146 | 18 | 0.00% | 0.00% | 0.00% | 1 | 1 | 1 | 1 | 1 | 1 | CG32206-PB, isoform B (LOC550984), mRNA |
| GB20062 | 11 | 0.00% | 0.00% | 0.00% | 1 | 1 | 1 | 1 | 1 | 1 | brahma CG5942-PA, isoform A (LOC551881), partial mRNA |
| GB16207 | 6 | 0.00% | 0.00% | 1.00% | 1 | 1 | 1 | 1 | 1 | 1 | CG14322-PA (LOC552581), mRNA |
| GB12459 | 34 | 0.00% | 0.02% | 0.00% | 1 | 1 | 1 | 1 | 1 | 1 | dynein axonemal intermediate chain |
| GB14461 | 5 | 0.00% | 0.00% | 0.38% | 1 | 1 | 1 | 1 | 1 | 2 | hypothetical LOC408407 (LOC408407), mRNA (short peptide) |
| GB13272 | 3 | 0.00% | 0.56% | 1.00% | 2 | 1 | 1 | 1 | 3 | 1 | cAMP-dependent protein kinase R1 CG3263-PC, isoform C, |
| GB16684 | 7 | 0.00% | 0.09% | 0.22% | 2 | 1 | 1 | 5 | 1 | 7 | CG10638-PA, isoform A (LOC412163), mRNA |
| GB12244 | 76 | 0.00% | 0.00% | 0.00% | 1 | 1 | 1 | 1 | 1 | 1 | Nuclear receptor coactivator 6 (Activating signal cointegrator 2) (ASC-2) Peroxisome proliferator-activated receptor-interacting |
| GB14250 | 13 | 0.00% | 0.00% | 0.16% | 1 | 1 | 1 | 1 | 1 | 1 | Ect4 CG7915-PB, isoform B (LOC410954), partial mRNA |
| GB15304 | 19 | 0.00% | 0.00% | 0.07% | 10 | 5 | 3 | 6 | 10 | 2 | CG13349-PA, isoform A (LOC413299), mRNA |
| GB12680 | 16 | 0.00% | 0.00% | 0.04% | 1 | 1 | 1 | 1 | 1 | 1 | pollux CG1093-PA, isoform A (LOC409346), mRNA |
| GB16921 | 17 | 0.00% | 0.00% | 0.01% | 1 | 1 | 1 | 1 | 6 | 1 | CG32133-PA (LOC411600), mRNA |
| GB12334 | 23 | 0.00% | 0.00% | 0.00% | 1 | 1 | 1 | 1 | 2 | 1 | Zinc finger FYVE domain-containing protein 1 |
| GB19824 | 4 | 0.00% | 0.00% | 0.39% | 1 | 1 | 1 | 1 | 1 | 1 | Mob1 CG11711-PD, isoform D (LOC408876), mRNA |
| GB16484 | 13 | 0.00% | 0.00% | 0.01% |  |  |  |  |  |  | serine protease homolog 37 |
| GB13078 | 12 | 0.00% | 0.00% | 0.01% | 1 | 1 | 1 | 1 | 3 | 1 | maggie CG14981-PA, isoform A (LOC409500), mRNA |
| GB13209 | 4 | 0.00% | 0.01% | 0.95% | 1 | 1 | 1 | 1 | 1 | 1 | F18A12.8a (LOC411846), mRNA |
| GB17086 | 13 | 0.00% | 0.00% | 0.13% | 1 | 1 | 1 | 1 | 1 | 1 | T-related protein (Trp) (Protein brachyenteron) (LOC412976), mRNA |
| GB11452 | 9 | 0.00% | 0.00% | 0.10% | 1 | 1 | 1 | 1 | 1 | 1 | MICAL CG33208-PB, isoform B, transcript variant 1 (LOC413310), mRNA |
| GB20083 | 3 | 0.00% | 0.00% | 0.05% | 1 | 1 | 1 | 1 | 1 | 1 | highly conserved unknown function (in rat and mouse described as apoptogenic 1) |
| GB15239 | 21 | 0.00% | 0.00% | 0.11% | 1 | 1 | 1 | 1 | 1 | 1 | liquid facets-related ( Epsin family) |
| GB19269 | 14 | 0.00% | 0.00% | 0.00% | 1 | 1 | 1 | 1 | 1 | 1 | zinc finger protein |
| GB16571 | 2 | 0.00% | 0.32% | 1.00% | 1 | 1 | 1 | 1 | 3 | 1 | yolkless CG1372-PA, isoform A (LOC725920), mRNA |
| GB19080 | 14 | 0.00% | 0.05% | 0.10% | 1 | 1 | 1 | 1 | 1 | 1 | Sas10 CG4202-PA (LOC413529), mRNA |
| GB17394 | 6 | 0.00% | 1.00% | 1.00% | 1 | 1 | 1 | 1 | 1 | 1 | ATP-binding cassette, sub-family A (ABC1), member 1 (LOC410829), mRNA |
| GB19187 | 19 | 0.00% | 0.00% | 0.09% | 1 | 1 | 1 | 1 | 1 | 1 | Mothers against decapentaplegic homolog 6 (SMAD 6) (Mothers against DPP homolog 6) (Smad6) (hSMAD6) |
| GB20128 | 8 | 0.00% | 0.00% | 0.00% | 1 | 1 | 1 | 1 | 1 | 1 | CG4040-PA (LOC409604), mRNA |
| GB20128 | 8 | 0.00% | 0.00% | 0.00% | 1 | 1 | 1 | 1 | 1 | 1 | CG4040-PA (LOC409604), mRNA |
| GB12664 | 20 | 0.00% | 0.00% | 0.00% | 1 | 1 | 1 | 1 | 1 | 1 | zinc finger protein |
| GB17972 | 6 | 0.00% | 0.81% | 1.00% | 1 | 1 | 1 | 1 | 1 | 1 | protein kinase C, transcript variant 1 (Pkc), mRNA |
| GB10427 | 55 | 0.00% | 0.00% | 0.00% | 1 | 1 | 1 | 1 | 1 | 1 | ROW (relative of woc) |
| GB14804 | 21 | 0.00% | 0.00% | 0.00% | 1 | 1 | 1 | 1 | 10 | 1 | vasa protein (Vasa), mRNA |
| GB19309 | 8 | 0.00% | 0.00% | 0.54% | 1 | 1 | 1 | 1 | 1 | 1 | RAB11 family interacting protein 4 (class II) (LOC409274), mRNA |
| GB13153 | 30 | 0.00% | 0.00% | 0.00% | 1 | 1 | 1 | 1 | 1 | 1 | CG8789-PA, isoform A (LOC410119), mRNA |
| GB13729 | 19 | 0.00% | 0.00% | 0.02% | 1 | 1 | 1 | 1 | 1 | 1 | sec15 CG7034-PA (LOC410084), mRNA |
| GB14209 | 52 | 0.00% | 0.00% | 0.00% | 1 | 1 | 1 | 1 | 1 | 1 | tuberous sclerosis 2 isoform 3 (LOC412278), mRNA |
| GB12127 | 31 | 0.00% | 0.00% | 0.17% | 1 | 1 | 1 | 1 | 2 | 1 | APG9 autophagy 9-like 1 (LOC412115), mRNA |
| GB14788 | 25 | 0.00% | 0.00% | 0.00% | 2 | 1 | 1 | 1 | 3 | 1 | hypothetical LOC551146 (LOC551146), mRNA |
| GB11426 | 19 | 0.00% | 0.00% | 0.11% | 1 | 1 | 1 | 1 | 1 | 1 | activated Cdc42-associated kinase |
| GB15126 | 8 | 0.00% | 0.00% | 0.05% | 1 | 1 | 1 | 1 | 1 | 1 | transposase?? (shaky annotation, small peptide) |
| GB19411 | 27 | 0.00% | 0.00% | 0.00% | 1 | 1 | 1 | 1 | 1 | 1 | Protein tyrosine phosphatase 69D CG10975-PB, isoform B (LOC725118), mRNA |
| GB19411 | 27 | 0.00% | 0.00% | 0.00% | 1 | 1 | 1 | 1 | 1 | 1 | Protein tyrosine phosphatase 69D CG10975-PB, isoform B (LOC725118), mRNA |
| GB12867 | 18 | 0.00% | 0.00% | 0.00% |  |  |  |  |  |  | similar to PRIP-interacting protein PIPMT |
| GB30193 | 72 | 0.00% | 0.00% | 0.00% | 1 | 1 | 1 | 1 | 2 | 1 | nejire CG15319-PB (LOC726280), mRNA |
| GB18171 | 6 | 0.00% | 0.45% | 1.00% | 1 | 1 | 1 | 1 | 1 | 1 | ultraviolet-sensitive opsin (Uvop), mRNA |
| GB10698 | 16 | 0.00% | 0.00% | 0.00% | 1 | 1 | 1 | 1 | 1 | 1 | lace CG4162-PA (LOC411447), partial mRNA |
| GB15763 | 10 | 0.00% | 0.00% | 0.52% | 1 | 1 | 1 | 1 | 1 | 1 | fibroblast growth factor receptor substrate 2 (LOC727079), mRNA |
| GB16783 | 29 | 0.00% | 0.00% | 0.09% | 1 | 1 | 1 | 1 | 1 | 1 | CG7940-PA (LOC551521), mRNA |
| GB19160 | 30 | 0.00% | 0.00% | 0.00% | 1 | 1 | 1 | 1 | 3 | 1 | Mediator complex subunit 25 CG12254-PA (LOC411545), mRNA |
| GB14402 | 9 | 0.00% | 0.02% | 0.07% | 1 | 1 | 1 | 1 | 1 | 1 | CG6043-PD, isoform D, transcript variant 2 (LOC551799), mRNA |
| GB16263 | 5 | 0.00% | 0.00% | 0.00% | 1 | 2 | 1 | 1 | 2 | 1 | Amphiphysin CG8604-PA (LOC409851), mRNA |
| GB10958 | 5 | 0.00% | 0.37% | 0.07% | 1 | 1 | 1 | 1 | 1 | 1 | CG10395-PA, isoform A (LOC414002), mRNA zinc finger HIT domain |
| GB16907 | 6 | 0.00% | 0.44% | 0.57% | 1 | 1 | 1 | 1 | 1 | 1 | CG3033-PA (LOC552360), mRNA |
| GB18099 | 47 | 0.00% | 0.00% | 0.00% | 1 | 1 | 1 | 1 | 1 | 1 | Nuclear pore complex protein Nup205 (Nucleoporin Nup205) (205 kDa nucleoporin) |
| GB16539 | 8 | 0.00% | 0.00% | 0.06% | 1 | 1 | 1 | 1 | 1 | 1 | CG2162-PA (LOC551984), mRNA |
| GB16539 | 8 | 0.00% | 0.00% | 0.06% | 1 | 1 | 1 | 1 | 1 | 1 | CG2162-PA (LOC551984), mRNA |
| GB11460 | 5 | 0.00% | 0.84% | 0.72% | 1 | 1 | 1 | 1 | 1 | 1 | ???? |
| GB16990 | 15 | 0.00% | 0.00% | 0.00% | 1 | 1 | 1 | 1 | 1 | 1 | cleavage and polyadenylation specific factor 1 (LOC551997), mRNA |
| GB11611 | 10 | 0.00% | 0.00% | 0.02% | 1 | 1 | 1 | 1 | 1 | 1 | CG12393-PA, isoform A (LOC412343), mRNA |
| GB14241 | 15 | 0.00% | 0.00% | 0.28% | 1 | 1 | 1 | 1 | 1 | 1 | Tango CG11098-PA, isoform A (LOC412103), mRNA transport and golgi organization 1 |
| GB17246 | 24 | 0.00% | 0.00% | 0.38% | 1 | 1 | 1 | 1 | 1 | 1 | unc-13-4A CG32381-PA (LOC408914), mRNA |
| GB11055 | 37 | 0.00% | 0.00% | 0.00% | 2 | 1 | 1 | 6 | 9 | 2 | poly A binding protein, cytoplasmic 1, transcript variant 1 (LOC412602), mRNA |
| GB11981 | 25 | 0.00% | 0.00% | 0.00% | 1 | 1 | 1 | 1 | 1 | 1 | cap-n-collar CG17894-PC, isoform C (LOC725081), mRNA |
| GB17335 | 22 | 0.00% | 0.00% | 0.00% | 1 | 2 | 1 | 1 | 1 | 1 | CG5594-PA, isoform A, transcript variant 1 (LOC411113), mRNA |
| GB14324 | 2 | 0.00% | 0.23% | 1.00% | 1 | 1 | 1 | 3 | 2 | 1 | Adenosylhomocysteinase at 13 CG11654-PA (LOC408368), mRNA |
| GB10233 | 19 | 0.00% | 0.01% | 0.00% | 1 | 1 | 1 | 1 | 1 | 1 | Pleckstrin homology domain-containing family G member 1 (LOC413248), mRNA |
| GB14294 | 9 | 0.00% | 0.82% | 0.68% | 1 | 1 | 1 | 1 | 1 | 1 | SET domain containing 3 (LOC410155), mRNA |
| GB19318 | 4 | 0.00% | 0.68% | 1.00% | 1 | 1 | 1 | 1 | 1 | 1 | ADAMTS-7 precursor (A disintegrin and metalloproteinase with thrombospondin motifs 7) (ADAM-TS 7) |
| GB14389 | 4 | 0.00% | 0.62% | 1.00% | 1 | 1 | 1 | 1 | 1 | 1 | CG6282-PA, isoform A, transcript variant 1 (LOC409642), mRNA |
| GB19982 | 5 | 0.00% | 0.98% | 1.00% | 1 | 1 | 1 | 1 | 1 | 1 | faint sausage (morphogenesis) |
| GB15190 | 10 | 0.00% | 0.00% | 0.04% | 1 | 1 | 1 | 1 | 1 | 1 | CG9809-PA, isoform A (LOC727127), mRNA |
| GB19570 | 11 | 0.00% | 0.00% | 0.39% | 1 | 1 | 1 | 2 | 4 | 1 | RuvB-like protein 1, transcript variant 1 (LOC409544), mRNA |
| GB10580 | 8 | 0.00% | 0.49% | 1.00% | 2 | 4 | 1 | 1 | 2 | 1 | hypothetical LOC551933 (LOC551933), mRNA |
| GB17511 | 18 | 0.00% | 0.00% | 0.00% | 2 | 1 | 1 | 1 | 3 | 1 | X box binding protein-1 |
| GB14018 | 4 | 0.00% | 0.90% | 1.00% | 1 | 1 | 1 | 1 | 1 | 1 | voltage-dependent anion channel 2 (LOC724641), mRNA |
| GB17429 | 44 | 0.01% | 0.00% | 0.00% | 1 | 1 | 1 | 1 | 1 | 1 | Phosphotidylinositol 3 kinase 68D CG11621-PA, isoform A |
| GB12214 | 9 | 0.01% | 0.00% | 0.27% | 1 | 1 | 1 | 1 | 2 | 1 | Mlx interactor CG18362-PC, isoform C (LOC410953), mRNA |
| GB16044 | 18 | 0.01% | 0.00% | 0.01% | 1 | 1 | 1 | 1 | 1 | 1 | CG14562-PA (LOC724591), mRNA |
| GB30207 | 5 | 0.01% | 0.98% | 1.00% | 1 | 1 | 1 | 1 | 1 | 1 | nervy CG3385-PA (LOC726335), mRNA |
| GB18960 | 7 | 0.01% | 0.56% | 1.00% | 2 | 2 | 1 | 1 | 2 | 10 | CG6439-PA (LOC552128), mRNA |
| GB17200 | 12 | 0.01% | 0.00% | 0.03% | 1 | 1 | 1 | 1 | 1 | 1 | RIKEN cDNA 0610040D20, transcript variant 1 |
| GB14826 | 16 | 0.01% | 0.21% | 0.61% | 1 | 1 | 1 | 1 | 1 | 1 | sorting nexin 8 (LOC412842), mRNA |
| GB14541 | 10 | 0.01% | 0.00% | 0.02% | 1 | 1 | 1 | 1 | 1 | 1 | dikar CG32393-PA, isoform A (LOC409504), partial mRNA |
| GB10123 | 18 | 0.01% | 0.00% | 0.00% | 1 | 1 | 1 | 1 | 3 | 1 | eIF3-S9 CG4878-PB, isoform B, transcript variant 1 |
| GB19799 | 4 | 0.01% | 0.91% | 1.00% | 1 | 1 | 1 | 3 | 2 | 1 | Vanin-like protein 1 precursor (LOC413631), partial mRNA |
| GB10953 | 13 | 0.01% | 0.00% | 0.07% | 2 | 2 | 1 | 1 | 2 | 1 | KDEL (Lys-Asp-Glu-Leu) containing 1 (LOC552603), mRNA |
| GB13730 | 10 | 0.01% | 0.00% | 0.03% | 1 | 1 | 1 | 1 | 2 | 1 | GDP dissociation inhibitor CG4422-PA (LOC411765), mRNA |
| GB16566 | 11 | 0.01% | 0.00% | 0.20% | 1 | 1 | 1 | 1 | 2 | 1 | Phosphoribosylamidotransferase CG2867-PA (LOC412619), mRNA |
| GB13225 | 35 | 0.01% | 0.00% | 0.00% | 1 | 1 | 1 | 1 | 2 | 1 | CG10492-PA (LOC725864), mRNA |
| GB19455 | 20 | 0.01% | 0.00% | 0.65% | 1 | 1 | 1 | 1 | 1 | 1 | NIMA (never in mitosis gene a)-related kinase 8 (LOC412057), mRNA |
| GB18854 | 34 | 0.01% | 0.00% | 0.00% | 1 | 1 | 1 | 1 | 1 | 1 | CG5077-PA, isoform A (LOC552573), mRNA |
| GB13810 | 6 | 0.01% | 0.87% | 0.84% | 1 | 1 | 1 | 1 | 1 | 1 | Werner helicase interacting protein 1, transcript variant 1 (LOC409392), mRNA |
| GB13480 | 15 | 0.01% | 0.00% | 0.45% | 1 | 1 | 1 | 1 | 1 | 1 | CG14299-PA, isoform A (LOC727278), mRNA |
| GB10413 | 34 | 0.01% | 0.00% | 0.01% | 1 | 1 | 1 | 2 | 4 | 1 | Dodeca-satellite-binding protein 1 CG5170-PC, isoform C (LOC412111), mRNA |
| GB17248 | 5 | 0.01% | 0.75% | 1.00% | 1 | 1 | 1 | 1 | 1 | 1 | Sep15 and SelM are eukaryotic selenoproteins with a thioredoxin-like domain and a surface accessible active site redox motif |
| GB11061 | 16 | 0.01% | 0.00% | 0.01% | 1 | 1 | 1 | 6 | 1 | 1 | CG31133-PA (LOC413647), mRNA |
| GB15694 | 29 | 0.01% | 0.00% | 0.02% | 1 | 2 | 1 | 1 | 3 | 1 | ubiquitin specific protease 8 (LOC411362), mRNA |
| GB17838 | 11 | 0.01% | 0.56% | 0.28% | 1 | 1 | 1 | 1 | 2 | 10 | hypothetical protein LOC725364 (LOC725364), mRNA |
| GB10712 | 4 | 0.01% | 0.03% | 1.00% |  |  |  |  |  |  | Zinc finger protein |
| GB30328 | 2 | 0.01% | 0.36% | 0.54% | 1 | 1 | 3 | 2 | 2 | 3 | Amino acid transporter |
| GB19405 | 6 | 0.01% | 0.00% | 0.17% | 1 | 1 | 1 | 1 | 1 | 1 | vacuolar H+ ATP synthase 16 kDa proteolipid subunit (Vha16), mRNA |
| GB16195 | 5 | 0.01% | 0.10% | 0.58% | 1 | 1 | 1 | 1 | 1 | 1 | ???? |
| GB15150 | 34 | 0.01% | 0.00% | 0.01% | 1 | 1 | 1 | 1 | 1 | 1 | CHIMERA - RhoGAP100F CG1976-PA, transcript variant 1 (LOC409836), mRNA + ATPase/transporter |
| GB16231 | 45 | 0.01% | 0.00% | 0.00% | 1 | 1 | 1 | 1 | 1 | 1 | CG5521-PA (LOC551830), partial mRNA |
| GB17892 | 20 | 0.01% | 0.00% | 0.01% | 1 | 1 | 1 | 1 | 1 | 1 | Proteasome-associated protein ECM29 homolog (Ecm29) (LOC725156), partial mRNA |
| GB18037 | 24 | 0.01% | 0.00% | 0.00% | 1 | 1 | 1 | 1 | 2 | 1 | CG2321-PA (LOC413154), mRNA |
| GB17120 | 14 | 0.01% | 0.00% | 0.00% | 1 | 1 | 1 | 1 | 1 | 1 | sodium chloride cotransporter 69 CG4357-PA, isoform A (LOC409208), mRNA |
| GB30294 | 8 | 0.01% | 0.00% | 0.03% | 1 | 1 | 1 | 1 | 1 | 1 | Transcription initiation factor IIF alpha subunit (TFIIF-alpha) (Transcription factor 5 large chain) (TF5A) |
| GB17055 | 26 | 0.01% | 0.00% | 0.00% | 1 | 1 | 1 | 1 | 1 | 1 | ??? |
| GB14380 | 46 | 0.01% | 0.00% | 0.00% | 1 | 1 | 1 | 1 | 2 | 1 | zinc finger protein 748 isoform 1 (LOC726882), mRNA |
| GB18860 | 5 | 0.01% | 1.00% | 0.60% | 1 | 1 | 1 | 1 | 1 | 1 | Osmotic avoidance abnormal protein 3 (Kinesin-like protein osm-3) |
| GB15722 | 5 | 0.01% | 0.00% | 0.03% |  |  |  |  |  |  | Glutamate NMDA receptor 2 |
| GB14612 | 2 | 0.01% | 1.00% | 1.00% | 3 | 1 | 1 | 1 | 1 | 1 | Probable cytochrome P450 6a14 (CYPVIA14) (LOC552418), mRNA |
| GB15197 | 4 | 0.01% | 0.82% | 1.00% | 1 | 1 | 1 | 1 | 1 | 1 | Tetraspanin 26A CG9093-PA (LOC551898), mRNA |
| GB18394 | 5 | 0.01% | 0.92% | 1.00% | 1 | 1 | 1 | 1 | 1 | 1 | cGMP-dependent protein kinase foraging (For), mRNA |
| GB16839 | 2 | 0.01% | 0.98% | 1.00% | 1 | 2 | 1 | 1 | 5 | 1 | CG8026-PA, isoform A (LOC410062), mRNA |
| GB11299 | 14 | 0.01% | 0.00% | 0.02% | 4 | 2 | 1 | 9 | 6 | 9 | Ribosomal protein L19 CG2746-PA, isoform A |
| GB11299 | 14 | 0.01% | 0.00% | 0.02% | 4 | 2 | 1 | 9 | 6 | 9 | Ribosomal protein L19 CG2746-PA, isoform B |
| GB11019 | 19 | 0.01% | 0.00% | 0.05% | 1 | 1 | 1 | 1 | 1 | 1 | CG32165-PA (LOC412817), mRNA |
| GB20091 | 8 | 0.01% | 0.00% | 0.42% | 1 | 1 | 1 | 1 | 1 | 1 | Proteasome-associated protein ECM29 homolog (Ecm29) (LOC725156), partial mRNA |
| GB18478 | 10 | 0.01% | 0.27% | 0.04% | 1 | 1 | 1 | 1 | 1 | 1 | Tusp CG5586-PB (LOC409680), mRNA |
| GB13607 | 3 | 0.01% | 0.99% | 1.00% | 1 | 1 | 1 | 1 | 1 | 1 | spaghetti CG13570-PA (LOC727022), mRNA |
| GB19651 | 14 | 0.01% | 0.00% | 0.15% | 1 | 1 | 1 | 1 | 1 | 1 | sorting nexin 14 (LOC551297), mRNA |
| GB14874 | 15 | 0.01% | 0.00% | 0.77% | 1 | 1 | 1 | 1 | 3 | 1 | Suppressor of profilin 2 CG8978-PA, isoform A (LOC408901), mRNA |
| GB14817 | 13 | 0.01% | 0.00% | 0.00% | 1 | 1 | 1 | 1 | 3 | 1 | schizo CG32434-PB, isoform B (LOC413410), mRNA |
| GB13704 | 43 | 0.01% | 0.00% | 0.00% | 2 | 4 | 1 | 1 | 1 | 3 | CG7766-PB, isoform B, transcript variant 1 (LOC550706), mRNA |
| GB13592 | 3 | 0.01% | 0.53% | 0.72% | 1 | 1 | 1 | 1 | 1 | 1 | kinesin light chain |
| GB15660 | 6 | 0.01% | 0.02% | 0.07% | 6 | 3 | 1 | 6 | 8 | 3 | Ribosomal protein L27 CG4759-PA, transcript variant 1 (LOC412266), mRNA |
| GB14335 | 7 | 0.01% | 0.00% | 0.36% | 1 | 1 | 1 | 1 | 1 | 1 | GLTP glycolipid transfer protein |
| GB17261 | 21 | 0.01% | 0.00% | 0.00% | 1 | 1 | 1 | 1 | 1 | 1 | General transcription factor TFIIICalfa (hoghly conserved) |
| GB19592 | 25 | 0.01% | 0.00% | 0.00% | 1 | 1 | 1 | 1 | 1 | 1 | CG6903-PA (LOC413119), mRNA |
| GB16403 | 64 | 0.01% | 0.00% | 0.00% | 1 | 1 | 1 | 1 | 1 | 1 | MYC binding protein 2 (LOC551317), mRNA |
| GB11001 | 8 | 0.01% | 0.00% | 0.32% | 3 | 1 | 1 | 1 | 1 | 1 | CG9849-PA (LOC408531), mRNA |
| GB12207 | 5 | 0.01% | 0.90% | 1.00% | 1 | 1 | 1 | 1 | 1 | 1 | epidermal growth factor receptor |
| GB12357 | 6 | 0.01% | 0.02% | 0.51% | 1 | 1 | 1 | 1 | 1 | 1 | Serine/threonine/tyrosine-interacting protein (Protein tyrosine phosphatase-like protein) |
| GB11701 | 10 | 0.01% | 0.00% | 0.22% | 1 | 1 | 1 | 1 | 1 | 1 | AP-50 CG7057-PA, isoform A, transcript variant 1 (LOC408418), mRNA |
| GB12498 | 7 | 0.01% | 0.00% | 0.05% | 1 | 1 | 1 | 1 | 1 | 1 | Myb protein (LOC409733), mRNA |
| GB20064 | 16 | 0.01% | 0.00% | 0.00% |  |  |  |  |  |  | RecQ4 protein |
| GB19066 | 2 | 0.01% | 1.00% | 1.00% |  |  |  |  |  |  | dorsal |
| GB30122 | 16 | 0.01% | 0.00% | 0.00% | 1 | 1 | 1 | 1 | 1 | 1 | RecQ4 CG7487-PA (LOC410301), mRNA |
| GB18656 | 24 | 0.01% | 0.00% | 0.00% | 1 | 1 | 1 | 1 | 1 | 1 | Highly conserved with WD-40 domain |
| GB15838 | 11 | 0.01% | 0.00% | 0.09% | 1 | 1 | 1 | 1 | 1 | 1 | CG12773-PA (LOC413689), mRNA |
| GB11286 | 16 | 0.01% | 0.00% | 0.03% | 1 | 1 | 1 | 1 | 1 | 1 | CG11594-PA, isoform A, transcript variant 1 (LOC411100), mRNA |
| GB11775 | 16 | 0.01% | 0.00% | 0.04% | 1 | 1 | 1 | 1 | 3 | 1 | vacuolar peduncle CG9209-PB, isoform B (LOC410811), mRNA |
| GB30340 | 28 | 0.01% | 0.00% | 0.07% | 1 | 1 | 1 | 1 | 1 | 1 | yurt CG9764-PA (LOC551579), mRNA |
| GB12107 | 15 | 0.01% | 0.00% | 0.15% | 1 | 1 | 1 | 1 | 1 | 1 | pasilla CG8144-PA, isoform A (LOC410165), mRNA |
| GB10422 | 30 | 0.01% | 0.00% | 0.00% | 1 | 1 | 1 | 1 | 1 | 1 | tamas CG8987-PA (LOC411763), mRNA |
| GB11387 | 7 | 0.01% | 0.13% | 0.09% | 1 | 1 | 1 | 1 | 1 | 1 | NOT CONSERVED - non coding RNA? |
| GB12228 | 58 | 0.01% | 0.00% | 0.00% |  |  |  |  |  |  | Bromodomain, cbp_like subfamily. Cbp (CREB binding protein or CREBBP) KAT domain-histone acetylation |
| GB30193 | 58 | 0.01% | 0.00% | 0.00% | 1 | 1 | 1 | 1 | 2 | 1 | nejire CG15319-PB (LOC726280), mRNA |
| GB18244 | 6 | 0.01% | 0.93% | 0.78% | 1 | 1 | 1 | 1 | 1 | 1 | possibly a G protein-coupled receptor (7tm_3 superfamily) |
| GB10446 | 7 | 0.01% | 0.00% | 0.92% | 1 | 2 | 1 | 2 | 2 | 1 | CG11750-PA (LOC551833), mRNA |
| GB13598 | 5 | 0.01% | 0.98% | 1.00% | 1 | 1 | 1 | 1 | 1 | 1 | Integrin alpha-PS2 precursor (Position-specific antigen 2 alpha chain) (Protein inflated) (LOC724548), mRNA |
| GB12975 | 35 | 0.01% | 0.00% | 0.11% | 1 | 1 | 1 | 1 | 1 | 1 | CG32843-PA (LOC412591), partial mRNA |
| GB10021 | 7 | 0.01% | 0.00% | 0.11% | 1 | 1 | 1 | 1 | 1 | 1 | Syntaxin 13 CG11278-PA (LOC551222), mRNA |
| GB10399 | 21 | 0.01% | 0.00% | 0.00% | 1 | 1 | 1 | 1 | 1 | 1 | hypothetical LOC552275 (LOC552275), mRNA |
| GB16210 | 3 | 0.01% | 0.00% | 1.00% | 1 | 1 | 1 | 1 | 1 | 1 | C-terminal Src kinase CG17309-PB, isoform B (LOC409908), mRNA |
| GB16540 | 7 | 0.01% | 0.00% | 0.00% | 1 | 1 | 1 | 1 | 1 | 1 | CG5315-PA, isoform A (LOC550659), mRNA |
| GB11229 | 3 | 0.01% | 0.16% | 1.00% | 1 | 1 | 1 | 1 | 1 | 1 | scribbler CG5580-PA, isoform A (LOC409729), mRNA |
| GB18813 | 4 | 0.01% | 1.00% | 1.00% | 1 | 1 | 1 | 1 | 1 | 1 | antennapedia protein (Antp), mRNA |
| GB11832 | 18 | 0.01% | 0.00% | 0.00% | 1 | 1 | 1 | 1 | 1 | 1 | CG5208-PA (LOC411850), mRNA |
| GB11484 | 6 | 0.01% | 0.07% | 0.60% | 2 | 1 | 1 | 1 | 2 | 1 | estrogen receptor binding protein (LOC412294), mRNA |
| GB13646 | 55 | 0.01% | 0.00% | 0.00% | 2 | 1 | 1 | 1 | 2 | 1 | CG31132-PA (LOC412406), mRNA |
| GB10342 | 2 | 0.01% | 0.87% | 1.00% | 1 | 1 | 1 | 1 | 1 | 1 | tweety CG1693-PA, isoform A (LOC413358), mRNA |
| GB13887 | 9 | 0.01% | 0.00% | 0.49% | 1 | 1 | 1 | 1 | 1 | 1 | CG12024-PB, isoform B (LOC412504), mRNA |
| GB10847 | 35 | 0.01% | 0.00% | 0.00% | 1 | 1 | 1 | 1 | 1 | 1 | similar to PR-domain zinc finger protein 5 |
| GB13961 | 5 | 0.01% | 0.04% | 0.29% | 1 | 1 | 1 | 1 | 1 | 1 | P7 protein (LOC724999), mRNA |
| GB17989 | 5 | 0.01% | 0.96% | 1.00% | 1 | 1 | 1 | 1 | 1 | 1 | DE-cadherin precursor (Protein shotgun) (LOC410585), mRNA |
| GB15153 | 2 | 0.01% | 0.59% | 1.00% | 1 | 1 | 1 | 1 | 1 | 1 | CG33635-PA, transcript variant 1 (LOC411493), mRNA |
| GB15224 | 42 | 0.01% | 0.00% | 0.00% | 1 | 1 | 1 | 1 | 1 | 1 | bromodomain containing 3 (LOC551826), mRNA |
| GB14239 | 8 | 0.01% | 0.00% | 0.10% | 1 | 1 | 1 | 1 | 1 | 1 | similar to Zinc finger protein (mammalian) |
| GB10685 | 10 | 0.01% | 0.00% | 0.28% |  |  |  |  |  |  | contains a few conserved motifs presernt in Drosophila nervy, CBFA2T1, human TAF105, human TAF130, fly TAF110. |
| GB14143 | 7 | 0.01% | 0.24% | 0.87% | 1 | 1 | 1 | 1 | 1 | 1 | F54C1.5a (LOC413930), mRNA |
| GB13720 | 3 | 0.01% | 0.82% | 1.00% | 1 | 1 | 1 | 1 | 1 | 1 | CG31814-PA (LOC411345), partial mRNA |
| GB12939 | 2 | 0.01% | 0.47% | 0.64% | 1 | 1 | 1 | 2 | 1 | 2 | Ferredoxin CG4205-PA (LOC552105), mRNA |
| GB15698 | 4 | 0.01% | 1.00% | 1.00% | 1 | 1 | 1 | 1 | 1 | 1 | eyegone CG10488-PA, isoform A (LOC410818), mRNA |
| GB15772 | 22 | 0.01% | 0.00% | 0.48% | 1 | 1 | 1 | 1 | 1 | 1 | F08G12.1 (LOC552625), mRNA |
| GB19930 | 11 | 0.01% | 0.01% | 0.01% | 1 | 1 | 1 | 1 | 1 | 1 | glutamine-dependent NAD(+) synthetase |
| GB14098 | 3 | 0.02% | 0.66% | 1.00% | 2 | 1 | 1 | 1 | 4 | 1 | CG6937-PA (LOC552509), mRNA |
| GB10633 | 9 | 0.02% | 0.00% | 0.00% | 1 | 1 | 1 | 1 | 1 | 1 | lola like CG5738-PA, isoform A (LOC725311), mRNA |
| GB10824 | 6 | 0.02% | 0.00% | 0.10% | 1 | 1 | 1 | 1 | 1 | 1 | ????? |
| GB18390 | 17 | 0.02% | 0.00% | 0.27% | 2 | 3 | 2 | 4 | 3 | 1 | Mystery 45A CG8070-PA, transcript variant 1 (LOC552280), mRNA |
| GB14132 | 10 | 0.02% | 0.04% | 0.01% | 1 | 1 | 1 | 1 | 1 | 1 | lethal (2) k07433 CG33130-PC, isoform C, transcript variant 1 (LOC408631), mRNA |
| GB30111 | 4 | 0.02% | 0.99% | 1.00% | 1 | 1 | 1 | 1 | 1 | 1 | otoferlin (LOC410318), mRNA |
| GB16333 | 6 | 0.02% | 0.04% | 0.96% | 3 | 5 | 1 | 1 | 2 | 1 | CG15270-PA, isoform A (LOC408908), mRNA |
| GB11782 | 8 | 0.02% | 0.53% | 0.40% | 1 | 1 | 1 | 1 | 1 | 1 | CG10711-PA (LOC412076), mRNA |
| GB13140 | 7 | 0.02% | 0.88% | 0.77% | 1 | 1 | 1 | 1 | 1 | 1 | CG8745-PA (LOC408817), mRNA |
| GB15650 | 4 | 0.02% | 0.94% | 1.00% | 1 | 1 | 1 | 1 | 1 | 1 | dpr6 CG14162-PA (LOC726106), mRNA |
| GB16479 | 16 | 0.02% | 0.00% | 0.00% | 1 | 1 | 1 | 1 | 1 | 1 | mitochondrial elongation factor G2 isoform 1 (LOC408831), mRNA |
| GB17141 | 7 | 0.02% | 0.00% | 0.12% | 1 | 1 | 1 | 1 | 2 | 1 | CG9253-PA, transcript variant 1 (LOC412190), mRNA |
| GB13940 | 13 | 0.02% | 0.00% | 0.02% | 1 | 1 | 1 | 1 | 1 | 1 | possibly paramyosin with DUF3659 |
| GB14467 | 4 | 0.02% | 0.53% | 0.00% | 1 | 1 | 1 | 1 | 1 | 1 | 2-oxoglutarate-dependent dioxygenases, requiring 2-oxoglutarate and dioxygen as cosubstrates and ferrous iron |
| GB15760 | 3 | 0.02% | 0.50% | 1.00% | 1 | 1 | 1 | 1 | 1 | 1 | Bardet-Biedl syndrome 4 (LOC551897), mRNA |
| GB12287 | 10 | 0.02% | 0.00% | 0.00% | 1 | 1 | 1 | 1 | 1 | 1 | big brain CG4722-PA (LOC413259), mRNA |
| GB14412 | 10 | 0.02% | 0.00% | 0.15% | 1 | 1 | 1 | 1 | 1 | 1 | CG4751-PA (LOC410446), mRNA |
| GB15714 | 28 | 0.02% | 0.00% | 0.00% | 1 | 1 | 1 | 1 | 1 | 1 | CG9311-PA (LOC551449), mRNA |
| GB13768 | 31 | 0.02% | 0.00% | 0.00% | 1 | 1 | 1 | 1 | 1 | 1 | DNA polymerase CG6768-PA (LOC409673), mRNA |
| GB18999 | 18 | 0.02% | 0.00% | 0.01% | 1 | 1 | 1 | 1 | 1 | 1 | CG7946-PA (LOC552332), mRNA |
| GB11197 | 9 | 0.02% | 0.54% | 0.01% | 1 | 1 | 1 | 1 | 1 | 1 | similar to Dynein heavy chain at 89D |
| GB15203 | 4 | 0.02% | 0.67% | 1.00% | 1 | 1 | 1 | 1 | 1 | 1 | CG15006-PA (LOC727204), mRNA |
| GB13381 | 37 | 0.02% | 0.00% | 0.00% |  |  |  |  |  |  | brm ATP-dependent helicase brahma (DEAD, bromodomian, etc) |
| GB10532 | 37 | 0.02% | 0.00% | 0.00% | 1 | 1 | 1 | 1 | 4 | 1 | CG1244-PA, isoform A (LOC409717), mRNA |
| GB14024 | 2 | 0.02% | 0.93% | 1.00% | 1 | 1 | 1 | 1 | 1 | 1 | CG30116-PB, isoform B (LOC409686), partial mRNA |
| GB16827 | 3 | 0.02% | 1.00% | 1.00% | 1 | 1 | 1 | 1 | 1 | 1 | black CG7811-PA (LOC411771), mRNA |
| GB19521 | 39 | 0.02% | 0.00% | 0.00% | 1 | 1 | 1 | 1 | 1 | 1 | zinc finger protein |
| GB10294 | 5 | 0.02% | 0.00% | 0.33% | 1 | 1 | 1 | 3 | 3 | 1 | CG5731-PA (LOC725899), mRNA |
| GB12884 | 3 | 0.02% | 0.39% | 1.00% | 1 | 1 | 1 | 1 | 1 | 1 | CG17090-PA, isoform A (LOC408664), mRNA |
| GB10095 | 3 | 0.02% | 0.27% | 1.00% | 1 | 1 | 1 | 1 | 1 | 1 | CG7042-PA (LOC725326), mRNA |
| GB15408 | 23 | 0.02% | 0.00% | 0.00% | 1 | 1 | 1 | 1 | 1 | 1 | CG8798-PA, isoform A (LOC409459), mRNA |
| GB19012 | 14 | 0.02% | 0.00% | 0.00% | 1 | 1 | 1 | 1 | 1 | 1 | ATP-binding cassette sub-family G member 4 (LOC550918), mRNA |
| GB11925 | 7 | 0.02% | 0.89% | 0.48% | 1 | 1 | 1 | 1 | 2 | 1 | sentrin/SUMO-specific protease 7 (LOC726735), partial mRNA |
| GB14650 | 6 | 0.02% | 0.96% | 0.15% | 2 | 1 | 1 | 1 | 1 | 1 | CG18317-PA, transcript variant 2 (LOC409122), mRNA |
| GB12158 | 4 | 0.02% | 0.80% | 1.00% | 1 | 1 | 1 | 6 | 1 | 1 | hypothetical protein LOC725217 (LOC725217), mRNA |
| GB10452 | 3 | 0.02% | 0.05% | 0.31% | 1 | 1 | 1 | 1 | 1 | 1 | CG2974-PA (LOC727471), mRNA |
| GB18661 | 28 | 0.02% | 0.00% | 0.00% | 1 | 1 | 1 | 1 | 1 | 1 | CG1677-PA (LOC409497), mRNA |
| GB17855 | 6 | 0.02% | 0.00% | 0.68% |  |  |  |  |  |  | DEAD-like helicases superfamily POLO-like |
| GB10400 | 29 | 0.02% | 0.00% | 0.00% | 1 | 1 | 1 | 1 | 1 | 1 | ubiquitin specific protease 48 isoform a (LOC409985), partial mRNA |
| GB10524 | 43 | 0.02% | 0.00% | 0.00% | 1 | 1 | 1 | 1 | 1 | 1 | domino CG9696-PD, isoform D (LOC413341), mRNA |
| GB10524 | 43 | 0.02% | 0.00% | 0.00% | 1 | 1 | 1 | 1 | 1 | 1 | domino CG9696-PD, isoform D (LOC413341), mRNA |
| GB13841 | 10 | 0.02% | 0.33% | 0.09% | 4 | 1 | 1 | 1 | 1 | 1 | Actin-related protein 14D CG9901-PA (LOC409313), mRNA |
| GB15421 | 6 | 0.02% | 1.00% | 1.00% | 1 | 1 | 1 | 1 | 1 | 1 | stripe CG7847-PA, isoform A (LOC726302), mRNA |
| GB18225 | 13 | 0.02% | 0.00% | 0.03% | 1 | 1 | 1 | 1 | 1 | 1 | CG7708-PA, isoform A (LOC409043), mRNA |
| GB14958 | 13 | 0.02% | 0.00% | 0.01% | 1 | 1 | 1 | 1 | 1 | 1 | selenide,water dikinase CG8553-PA, isoform A (LOC410258), mRNA |
| GB13239 | 15 | 0.02% | 0.00% | 0.08% | 1 | 1 | 1 | 1 | 1 | 1 | RNA-binding protein 2 CG4429-PA, isoform A (LOC409379), mRNA |
| GB13398 | 4 | 0.02% | 0.29% | 0.80% | 1 | 1 | 1 | 1 | 1 | 1 | ????? |
| GB13781 | 3 | 0.02% | 0.97% | 1.00% |  |  |  |  |  |  | transporter (monoamine in fly) |
| GB30021 | 3 | 0.02% | 0.97% | 1.00% | 1 | 1 | 1 | 1 | 1 | 1 | CG33528-PC, isoform C (LOC408517), mRNA |
| GB14163 | 63 | 0.02% | 0.00% | 0.00% | 1 | 1 | 1 | 1 | 1 | 1 | CG11533-PA, isoform A (LOC411575), mRNA |
| GB14163 | 63 | 0.02% | 0.00% | 0.00% | 1 | 1 | 1 | 1 | 1 | 1 | CG11533-PA, isoform A (LOC411575), mRNA |
| GB14275 | 7 | 0.02% | 0.90% | 0.11% | 1 | 1 | 1 | 1 | 1 | 1 | CG31640-PA (LOC411213), mRNA |
| GB15606 | 13 | 0.02% | 0.00% | 0.16% | 1 | 1 | 1 | 1 | 2 | 1 | p38b CG7393-PA (LOC411915), mRNA |
| GB15577 | 13 | 0.02% | 0.00% | 0.10% | 1 | 1 | 1 | 1 | 1 | 1 | Highly conserved (in fly: phagocytosis, engulfment) |
| GB14675 | 7 | 0.02% | 0.06% | 0.38% | 1 | 1 | 1 | 1 | 1 | 1 | THAP superfamily (THAP domain is a putative DNA-binding domain (DBD) and probably also binds zinc |
| GB14694 | 15 | 0.02% | 1.00% | 0.02% | 1 | 1 | 1 | 1 | 1 | 1 | similar to wing blister (EGF_Lam; Laminin-type epidermal growth factor-like domain) |
| GB13892 | 24 | 0.02% | 0.00% | 0.00% | 1 | 1 | 1 | 1 | 4 | 1 | Transcription initiation factor TFIID subunit 4 (Transcription initiation factor TFIID 110 kDa subunit |
| GB12785 | 33 | 0.02% | 0.00% | 0.01% |  |  |  |  |  |  | Syt20 synaptotagmin 20 |
| GB15598 | 8 | 0.02% | 0.00% | 0.13% | 1 | 1 | 1 | 1 | 1 | 1 | ???? |
| GB18360 | 15 | 0.02% | 0.01% | 0.26% | 1 | 1 | 1 | 1 | 1 | 1 | CG11796-PA, isoform A (LOC725400), mRNA |
| GB30043 | 9 | 0.02% | 0.48% | 0.12% |  |  |  |  |  |  | ASRGL1_like domains, a subfamily of the L-Asparaginase type 2-like enzymes (NCBI annotation) |
| GB13596 | 23 | 0.02% | 0.00% | 0.18% | 2 | 2 | 1 | 2 | 2 | 8 | ATP synthase- CG11154-PA, isoform A (LOC551766), mRNA |
| GB12095 | 3 | 0.02% | 0.91% | 1.00% |  |  |  |  |  |  | complexin (neurotransmitter transporter) |
| GB20017 | 29 | 0.02% | 0.00% | 0.00% | 1 | 1 | 1 | 1 | 1 | 1 | TER94 CG2331-PA, isoform A, transcript variant 1 (LOC409377), mRNA |
| GB17305 | 18 | 0.02% | 0.00% | 0.02% | 1 | 1 | 1 | 1 | 2 | 1 | Nuclear migration protein nudC (Nuclear distribution protein C homolog) (Silica-induced gene 92 protein) (SIG-92) |
| GB17302 | 14 | 0.02% | 0.00% | 0.12% | 1 | 2 | 1 | 2 | 6 | 2 | CG5684-PA, isoform A, transcript variant 1 (LOC551045), mRNA |
| GB16186 | 21 | 0.02% | 0.00% | 0.00% | 3 | 4 | 1 | 5 | 10 | 7 | CG6512-PB, isoform B (LOC552166), mRNA |
| GB15442 | 11 | 0.02% | 0.00% | 0.22% | 1 | 1 | 1 | 1 | 4 | 2 | CG1814-PA, isoform A (LOC410189), mRNA |
| GB11667 | 7 | 0.02% | 0.00% | 0.05% | 1 | 1 | 1 | 1 | 1 | 1 | RAD23a homolog, transcript variant 2 (LOC409340), mRNA |
| GB17411 | 7 | 0.02% | 0.00% | 1.00% | 1 | 1 | 1 | 1 | 1 | 1 | Septin-2 CG4173-PA (LOC408882), mRNA |
| GB19703 | 4 | 0.02% | 0.52% | 1.00% | 1 | 1 | 1 | 1 | 1 | 1 | nicotinic Acetylcholine Receptor 80B CG12414-PA (LOC726753), partial mRNA |
| GB17185 | 10 | 0.02% | 0.03% | 0.39% | 1 | 3 | 1 | 1 | 1 | 1 | CG7888-PB, isoform B, transcript variant 1 (LOC409640), mRNA |
| GB15597 | 21 | 0.02% | 0.00% | 0.00% | 1 | 1 | 1 | 1 | 1 | 1 | ether a go-go CG10952-PA (LOC552834), mRNA |
| GB18093 | 27 | 0.02% | 0.00% | 0.00% | 1 | 1 | 1 | 1 | 1 | 1 | Transcription factor Sp3 (SPR-2) (LOC551928), mRNA |
| GB19264 | 2 | 0.02% | 0.59% | 1.00% | 1 | 1 | 1 | 1 | 1 | 1 | period clock protein (Per), mRNA |
| GB19264 | 2 | 0.02% | 0.59% | 1.00% | 1 | 1 | 1 | 1 | 1 | 1 | period clock protein (Per), mRNA |
| GB12784 | 32 | 0.02% | 0.00% | 0.00% | 1 | 1 | 1 | 1 | 1 | 1 | CG3044-PA (LOC412245), mRNA |
| GB10982 | 4 | 0.02% | 0.81% | 1.00% | 1 | 1 | 1 | 1 | 1 | 1 | Protein cappuccino (LOC726681), mRNA |
| GB13845 | 5 | 0.02% | 0.64% | 0.61% | 1 | 1 | 1 | 1 | 1 | 1 | ????? |
| GB16972 | 10 | 0.02% | 0.01% | 0.12% | 1 | 1 | 1 | 1 | 1 | 1 | Protein NipSnap (LOC409442), mRNA |
| GB16808 | 27 | 0.02% | 0.00% | 0.00% | 1 | 1 | 1 | 1 | 1 | 1 | Zinc finger X-linked protein ZXDB (LOC724258), mRNA |
| GB30412 | 6 | 0.02% | 0.01% | 0.34% | 1 | 1 | 1 | 1 | 3 | 1 | ??????? |
| GB12956 | 7 | 0.02% | 0.00% | 0.29% | 2 | 3 | 3 | 10 | 2 | 1 | yippee interacting protein 2 CG4600-PA (LOC408291), mRNA |
| GB11760 | 30 | 0.02% | 0.00% | 0.00% | 1 | 1 | 1 | 1 | 1 | 1 | Calcineurin-binding protein Cabin 1 |
| GB14082 | 24 | 0.02% | 0.00% | 0.00% | 1 | 1 | 1 | 1 | 1 | 1 | galactosyltransferases UDP-galactose |
| GB18917 | 4 | 0.02% | 0.00% | 0.08% | 1 | 1 | 1 | 1 | 5 | 1 | Cofilin/actin-depolymerizing factor homolog (Protein D61) (Protein twinstar) (LOC725718), mRNA |
| GB15728 | 43 | 0.02% | 0.00% | 0.00% | 1 | 1 | 1 | 1 | 1 | 1 | shibire CG18102-PD, isoform D, transcript variant 1 (LOC410923), mRNA |
| GB18759 | 3 | 0.02% | 0.98% | 1.00% | 1 | 1 | 1 | 1 | 1 | 1 | similar to placental-like alkaline phosphatase preproprotein |
| GB11530 | 6 | 0.02% | 0.00% | 0.44% | 1 | 1 | 1 | 1 | 1 | 1 | ubiquitin-conjugating enzyme E2, J1 (LOC409794), mRNA |
| GB12559 | 7 | 0.02% | 0.39% | 0.27% | 1 | 1 | 1 | 1 | 1 | 1 | hypothetical LOC552171 (LOC552171), mRNA |
| GB13456 | 11 | 0.02% | 0.94% | 0.03% | 1 | 1 | 1 | 1 | 1 | 1 | Transducin-like enhancer protein 4 (Groucho-related protein 4) (Grg-4) (LOC411175), mRNA |
| GB10379 | 18 | 0.02% | 0.00% | 0.00% | 1 | 1 | 1 | 1 | 1 | 1 | smoothened CG11561-PA (LOC411904), mRNA |
| GB18978 | 16 | 0.02% | 0.00% | 0.09% | 1 | 1 | 1 | 1 | 1 | 1 | mutagen-sensitive 210 CG8153-PA, isoform A (LOC552498), mRNA |
| GB19382 | 21 | 0.02% | 0.84% | 0.00% | 1 | 1 | 1 | 1 | 1 | 1 | similar to dpr14 Ig superfamily (defective proboscis extension response ) J Neurosci. 2002 May 1;22(9):3463-72 |
| GB10109 | 3 | 0.02% | 0.00% | 0.01% | 1 | 2 | 1 | 2 | 2 | 2 | CG6404-PB, isoform B (LOC408349), mRNA |
| GB11674 | 9 | 0.02% | 0.00% | 0.47% | 1 | 1 | 1 | 1 | 1 | 1 | Grasp65 CG7809-PA (LOC409571), mRNA |
| GB16534 | 26 | 0.02% | 0.00% | 0.21% | 1 | 1 | 1 | 1 | 1 | 1 | mesoderm induction early response 1 (LOC409075), mRNA |
| GB10172 | 11 | 0.02% | 0.00% | 0.10% | 2 | 1 | 1 | 1 | 1 | 1 | HLA-B associated transcript 5 (LOC411908), mRNA |
| GB11437 | 3 | 0.02% | 0.88% | 1.00% | 2 | 1 | 1 | 1 | 1 | 1 | vacuolar protein sorting 29 isoform 2 (LOC725700), mRNA |
| GB14821 | 2 | 0.02% | 0.69% | 1.00% | 1 | 1 | 1 | 1 | 1 | 1 | Pancreatic lipase-related protein 2 precursor (Secretory glycoprotein GP-3), transcript variant 1 (LOC411609), mRNA |
| GB12594 | 9 | 0.02% | 0.00% | 0.29% | 1 | 1 | 1 | 1 | 1 | 1 | CG3817-PA (LOC724992), mRNA |
| GB14525 | 36 | 0.02% | 0.00% | 0.14% | 1 | 1 | 1 | 1 | 1 | 1 | crumbs CG6383-PA (LOC725591), mRNA |
| GB12441 | 8 | 0.02% | 0.00% | 0.25% | 10 | 4 | 8 | 10 | 10 | 10 | Ribosomal protein L35A CG2099-PA, transcript variant 1 (LOC409599), mRNA |
| GB10353 | 6 | 0.03% | 0.03% | 0.33% | 7 | 4 | 2 | 10 | 8 | 10 | Ribosomal protein S15 CG8332-PA, isoform A (LOC551644), mRNA |
| GB12305 | 5 | 0.03% | 0.00% | 0.68% | 1 | 1 | 1 | 1 | 2 | 1 | Transcription initiation factor TFIID subunit 11 (Transcription initiation factor TFIID 28 kDa subunit beta) (p28-beta) |
| GB10477 | 10 | 0.03% | 0.00% | 0.26% | 1 | 1 | 1 | 3 | 2 | 1 | B-cell receptor-associated protein 37 (LOC551944), mRNA |
| GB13021 | 26 | 0.03% | 0.00% | 0.00% | 1 | 1 | 1 | 1 | 3 | 1 | Membrane-associated protein Hem (dHem-2) (LOC551945), mRNA |
| GB13986 | 4 | 0.03% | 0.96% | 0.24% | 1 | 1 | 1 | 1 | 1 | 1 | SMN (survival motor neuron) TUDOR superfamily |
| GB17344 | 7 | 0.03% | 0.01% | 0.12% | 1 | 1 | 1 | 1 | 1 | 1 | Death-associated protein kinase 1 (DAP kinase 1) (LOC411980), mRNA |
| GB13046 | 3 | 0.03% | 1.00% | 1.00% | 1 | 1 | 1 | 1 | 1 | 1 | CG15373-PA (LOC412436), mRNA |
| GB16576 | 10 | 0.03% | 0.00% | 0.06% | 1 | 1 | 1 | 1 | 2 | 1 | thyroid hormone receptor associated protein 5 (LOC551212), mRNA |
| GB17488 | 4 | 0.03% | 0.12% | 0.57% | 4 | 1 | 1 | 6 | 1 | 2 | 40S ribosomal protein S20 (LOC726013), mRNA |
| GB11791 | 6 | 0.03% | 0.42% | 0.04% | 1 | 1 | 1 | 1 | 1 | 1 | K11B4.1 (LOC726673), mRNA |
| GB19934 | 10 | 0.03% | 0.90% | 0.03% | 1 | 1 | 1 | 1 | 1 | 1 | metallo-dependent hydrolases, renal dipeptidase (rDP), best studied in mammals and also called microsomal dipeptidase |
| GB14312 | 4 | 0.03% | 1.00% | 1.00% | 1 | 1 | 1 | 1 | 1 | 1 | vestigial CG3830-PA (LOC726247), mRNA |
| GB12104 | 8 | 0.03% | 0.00% | 0.10% | 1 | 1 | 1 | 1 | 1 | 1 | CG9784-PA, transcript variant 1 (LOC408730), mRNA |
| GB14398 | 14 | 0.03% | 0.00% | 0.26% |  |  |  |  |  |  | Lysophospholipid Acyltransferase (LPLAT) |
| GB30530 | 14 | 0.03% | 0.00% | 0.26% | 1 | 2 | 1 | 2 | 2 | 1 | Lysophospholipid Acyltransferase (LPLAT) |
| GB15775 | 20 | 0.03% | 0.00% | 0.03% | 2 | 2 | 2 | 1 | 7 | 1 | MBD-R2 CG10042-PA, isoform A (LOC725950), mRNA |
| GB10759 | 13 | 0.03% | 1.00% | 0.13% | 1 | 1 | 1 | 1 | 5 | 1 | ecdysteroid-regulated gene E74 (E74), mRNA |
| GB18904 | 6 | 0.03% | 0.37% | 0.11% | 1 | 1 | 1 | 2 | 1 | 1 | Vanin-like protein 1 precursor (LOC724312), mRNA |
| GB30187 | 2 | 0.03% | 0.01% | 1.00% | 1 | 1 | 1 | 1 | 2 | 1 | CG32778-PA, transcript variant 1 (LOC410882), mRNA |
| GB12796 | 8 | 0.03% | 0.39% | 0.25% | 1 | 1 | 1 | 1 | 1 | 1 | Midasin (MIDAS-containing protein) (LOC551016), mRNA |
| GB10289 | 2 | 0.03% | 0.42% | 1.00% | 1 | 1 | 1 | 1 | 1 | 1 | Mpv17 protein (LOC727505), mRNA |
| GB14350 | 10 | 0.03% | 0.00% | 0.02% | 1 | 1 | 1 | 1 | 1 | 1 | putative chemosensory receptor 2 (R2), mRNA |
| GB17492 | 4 | 0.03% | 0.69% | 0.16% | 1 | 1 | 1 | 1 | 1 | 1 | NuA4 superfamily |
| GB19595 | 4 | 0.03% | 0.90% | 1.00% | 1 | 1 | 1 | 1 | 1 | 1 | SMC structural maintenance of chromosomes,bind DNA and act in organizing and segregating chromosomes |
| GB17088 | 10 | 0.03% | 0.05% | 0.18% | 1 | 1 | 1 | 2 | 3 | 3 | mitochondrial ribosomal protein S30 CG8470-PA (LOC412984), mRNA |
| GB10514 | 27 | 0.03% | 0.00% | 0.07% | 3 | 2 | 1 | 3 | 4 | 1 | Tubulin alpha-1 chain (LOC408388), mRNA |
| GB14139 | 23 | 0.03% | 0.00% | 0.00% | 1 | 1 | 1 | 1 | 1 | 1 | maleless CG11680-PA, isoform A (LOC413074), mRNA |
| GB16650 | 3 | 0.03% | 0.17% | 1.00% | 2 | 1 | 1 | 1 | 1 | 1 | CG17691-PA.3 (LOC409306), mRNA |
| GB14659 | 17 | 0.03% | 0.00% | 0.01% | 1 | 1 | 1 | 1 | 4 | 1 | apoptotic protease activating factor 1 [Homo sapiens] with WD40 |
| GB15644 | 14 | 0.03% | 0.00% | 0.03% | 1 | 1 | 1 | 1 | 1 | 1 | CG10600-PA (LOC551022), mRNA |
| GB12526 | 24 | 0.03% | 0.00% | 0.00% | 1 | 1 | 1 | 1 | 1 | 1 | lethal (2) k05713 CG8256-PC, isoform C (LOC551904), mRNA |
| GB16167 | 9 | 0.03% | 0.00% | 0.12% | 5 | 2 | 1 | 4 | 2 | 6 | Rlc1 CG9378-PB, isoform B, transcript variant 1 (LOC413774), mRNA |
| GB16680 | 29 | 0.03% | 0.00% | 0.00% | 1 | 1 | 1 | 1 | 1 | 1 | Alsin (Amyotrophic lateral sclerosis protein 2) (LOC413195), mRNA |
| GB15536 | 24 | 0.03% | 0.00% | 0.00% | 1 | 1 | 1 | 1 | 1 | 1 | CG5397-PA (LOC410187), mRNA |
| GB17320 | 17 | 0.03% | 0.00% | 0.00% | 1 | 1 | 1 | 1 | 5 | 1 | rapamycin-insensitive companion of Tor CG8002-PA (LOC413223), mRNA |
| GB17834 | 3 | 0.03% | 0.85% | 1.00% | 1 | 1 | 1 | 1 | 1 | 1 | conserved in insects |
| GB11822 | 4 | 0.03% | 0.90% | 1.00% | 1 | 1 | 1 | 1 | 1 | 1 | CG12075-PA, isoform A (LOC552005), mRNA |
| GB16815 | 2 | 0.03% | 0.46% | 1.00% | 1 | 3 | 1 | 1 | 2 | 2 | Ribonuclease H1 (RNase H1) (Ribonuclease H type II) (LOC412100), mRNA |
| GB15429 | 3 | 0.03% | 0.09% | 1.00% | 1 | 1 | 1 | 1 | 1 | 1 | CG13165-PA (LOC552195), mRNA |
| GB12713 | 10 | 0.03% | 0.92% | 0.71% | 1 | 1 | 1 | 1 | 1 | 1 | conserved in insects |
| GB12769 | 7 | 0.03% | 0.22% | 0.69% | 1 | 1 | 1 | 1 | 1 | 1 | tipE temperature-induced paralytic E |
| GB12967 | 3 | 0.03% | 1.00% | 1.00% | 1 | 1 | 1 | 1 | 1 | 1 | murashka CG9381-PA, isoform A, transcript variant 1 (LOC408543), mRNA |
| GB18891 | 18 | 0.03% | 0.00% | 0.07% | 1 | 1 | 1 | 1 | 1 | 1 | CG32549-PD, isoform D (LOC551770), partial mRNA |
| GB13300 | 4 | 0.03% | 0.79% | 1.00% | 1 | 1 | 1 | 1 | 1 | 1 | CG6674-PA (LOC726788), mRNA |
| GB17963 | 14 | 0.03% | 0.03% | 0.07% | 1 | 1 | 1 | 1 | 2 | 1 | ring finger protein 146 (LOC550997), mRNA |
| GB16778 | 14 | 0.03% | 0.84% | 0.01% | 1 | 1 | 1 | 1 | 1 | 1 | defective proboscis extension response CG13439-PA (LOC410479), partial mRNA |
| GB11557 | 17 | 0.03% | 0.00% | 0.03% | 1 | 1 | 1 | 1 | 1 | 1 | RuvB-like 2 (p47 protein) (LOC726816), mRNA |
| GB13183 | 3 | 0.03% | 0.56% | 0.90% | 1 | 1 | 1 | 1 | 1 | 1 | CG5147-PA (LOC552353), mRNA |
| GB10966 | 6 | 0.03% | 0.01% | 0.21% | 1 | 1 | 1 | 1 | 2 | 1 | Guanine nucleotide-binding protein G(i) subunit alpha 65A (LOC411704), mRNA |
| GB14881 | 36 | 0.03% | 0.00% | 0.00% | 1 | 1 | 1 | 1 | 1 | 1 | small optic lobes CG1391-PB, isoform B (LOC551614), mRNA |
| GB16033 | 6 | 0.03% | 0.71% | 0.28% | 1 | 1 | 1 | 1 | 1 | 1 | Polypeptide N-acetylgalactosaminyltransferase 35A CG7480-PA (LOC552496), mRNA |
| GB10810 | 51 | 0.03% | 0.00% | 0.00% | 1 | 1 | 1 | 1 | 1 | 1 | kohtalo CG8491-PA (LOC409271), mRNA |
| GB17204 | 2 | 0.03% | 0.72% | 1.00% | 1 | 1 | 1 | 1 | 4 | 1 | PDZ containing |
| GB17589 | 2 | 0.03% | 0.74% | 1.00% | 1 | 1 | 1 | 1 | 1 | 1 | short peptide also foun din Nasonia |
| GB15690 | 4 | 0.03% | 0.01% | 0.63% | 1 | 1 | 1 | 1 | 1 | 1 | CG6196-PA (LOC725904), mRNA |
| GB15758 | 2 | 0.03% | 0.46% | 1.00% | 1 | 1 | 1 | 1 | 1 | 1 | similarto a Nasonia protein (ppor annotation) |
| GB15560 | 53 | 0.03% | 0.00% | 0.00% |  |  |  |  |  |  | annotation problem: chimera? |
| GB30310 | 53 | 0.03% | 0.00% | 0.00% |  |  |  |  |  |  | annotation problem: chimera? |
| GB16985 | 2 | 0.03% | 0.04% | 0.19% | 1 | 1 | 1 | 1 | 1 | 1 | globin inducing factor, fetal (LOC408773), mRNA |
| GB18577 | 3 | 0.03% | 0.67% | 1.00% | 1 | 1 | 1 | 1 | 1 | 1 | enoyl Coenzyme A hydratase domain containing 3 (LOC551537), mRNA |
| GB16857 | 7 | 0.03% | 0.08% | 0.67% | 1 | 1 | 1 | 1 | 1 | 1 | adipokinetic hormone receptor (LOC551388), mRNA |
| GB30199 | 38 | 0.03% | 1.00% | 0.00% | 1 | 1 | 1 | 1 | 1 | 1 | dachsous CG17941-PA (LOC408767), mRNA |
| GB15676 | 3 | 0.03% | 0.94% | 0.01% | 1 | 1 | 1 | 1 | 1 | 1 | CG9990-PA, isoform A, transcript variant 1 (LOC409666), mRNA |
| GB16390 | 2 | 0.03% | 0.72% | 1.00% | 1 | 1 | 2 | 1 | 1 | 1 | 4GalNAcTA CG8536-PA (LOC411365), partial mRNA |
| GB16968 | 14 | 0.03% | 0.00% | 0.00% | 2 | 1 | 1 | 1 | 1 | 1 | Sad1_UNC |
| GB15986 | 6 | 0.03% | 0.00% | 0.04% | 2 | 3 | 1 | 3 | 4 | 2 | Clathrin light chain CG6948-PA (LOC411595), mRNA |
| GB14686 | 31 | 0.03% | 0.00% | 0.00% | 1 | 1 | 1 | 1 | 1 | 1 | Mediator complex subunit 24 CG7999-PA (LOC408458), mRNA |
| GB17770 | 12 | 0.03% | 0.00% | 0.06% | 1 | 1 | 1 | 1 | 2 | 1 | CDC45L CG3658-PA (LOC410073), mRNA |
| GB12637 | 9 | 0.03% | 0.00% | 0.38% | 8 | 1 | 1 | 9 | 5 | 2 | Ribosomal protein S9 CG3395-PA, isoform A (LOC409202), mRNA |
| GB19434 | 4 | 0.03% | 0.00% | 0.46% | 1 | 1 | 1 | 1 | 2 | 1 | Cell division control protein 2 homolog (p34 protein kinase) (LOC409590), mRNA |
| GB10352 | 22 | 0.03% | 0.00% | 0.00% | 1 | 1 | 1 | 1 | 1 | 1 | 6-phosphofructo-2-kinase CG3400-PG, isoform G (LOC409574), mRNA |
| GB12426 | 8 | 0.03% | 0.00% | 0.34% | 1 | 2 | 1 | 1 | 2 | 1 | single-strand selective monofunctional uracil DNA glycosylase (LOC413439), mRNA |
| GB15192 | 3 | 0.03% | 0.66% | 1.00% | 1 | 1 | 1 | 1 | 1 | 1 | CG9317-PA, isoform A (LOC410448), mRNA |
| GB19147 | 14 | 0.03% | 0.00% | 0.00% | 1 | 1 | 1 | 1 | 1 | 1 | WD repeat domain 10 isoform 3 (LOC727298), partial mRNA |
| GB16649 | 5 | 0.03% | 0.00% | 0.05% | 1 | 1 | 1 | 1 | 2 | 1 | Rox8 CG5422-PB, isoform B (LOC411890), mRNA |
| GB17526 | 8 | 0.03% | 0.42% | 0.47% | 1 | 1 | 1 | 1 | 1 | 1 | NADPH dependent diflavin oxidoreductase 1 (LOC413700), mRNA |
| GB12835 | 21 | 0.03% | 0.00% | 0.00% | 1 | 1 | 1 | 1 | 2 | 1 | CG11456-PA (LOC726063), mRNA |
| GB13033 | 15 | 0.03% | 0.00% | 0.29% | 1 | 1 | 1 | 1 | 1 | 1 | CG8258-PA (LOC551436), mRNA |
| GB11364 | 4 | 0.03% | 1.00% | 1.00% | 1 | 1 | 3 | 1 | 3 | 1 | Ecdysone-induced protein 75B CG8127-PD, isoform D (LOC410309), mRNA |
| GB17482 | 3 | 0.03% | 0.41% | 1.00% | 1 | 1 | 1 | 1 | 1 | 1 | beat-IV CG10152-PA (LOC410563), mRNA |
| GB20034 | 50 | 0.03% | 0.00% | 0.00% | 2 | 2 | 1 | 2 | 2 | 1 | CG8108-PB, isoform B (LOC725524), partial mRNA |
| GB15689 | 23 | 0.03% | 0.00% | 0.00% | 1 | 1 | 1 | 1 | 1 | 1 | UPF2 regulator of nonsense transcripts homolog (LOC413146), mRNA |
| GB16175 | 9 | 0.03% | 0.05% | 0.45% | 1 | 1 | 1 | 5 | 3 | 1 | nucleolin (LOC726610), mRNA |
| GB12153 | 4 | 0.03% | 1.00% | 1.00% | 1 | 1 | 1 | 1 | 1 | 1 | protein with WD40 and SMC |
| GB11574 | 40 | 0.03% | 0.00% | 0.01% | 1 | 1 | 1 | 1 | 4 | 1 | splicing factor 3b, subunit 3, transcript variant 1 (LOC550935), mRNA |
| GB19970 | 24 | 0.03% | 0.00% | 0.00% | 1 | 1 | 1 | 1 | 1 | 1 | SMC (structural maintenance of chromosomes) proteins bind DNA and act in organizing and segregating chromosomes for partition |
| GB19759 | 11 | 0.03% | 0.00% | 0.26% | 1 | 1 | 1 | 1 | 4 | 1 | CG10914-PA (LOC413531), partial mRNA |
| GB18491 | 2 | 0.03% | 0.46% | 1.00% | 1 | 1 | 1 | 1 | 1 | 1 | LSM5 homolog, U6 small nuclear RNA associated (LOC725641), mRNA |
| GB10672 | 14 | 0.03% | 0.00% | 0.01% | 1 | 1 | 1 | 1 | 1 | 1 | DNA topoisomerase 3-beta-1 (DNA topoisomerase III beta-1), transcript variant 2 (LOC552787), mRNA |
| GB12347 | 5 | 0.03% | 0.00% | 0.12% | 1 | 1 | 1 | 1 | 1 | 1 | splicing factor 3b, subunit 4 (LOC410434), mRNA |
| GB17850 | 7 | 0.03% | 0.00% | 0.48% | 1 | 1 | 1 | 1 | 1 | 1 | K[+]-channel tetramerization protein |
| GB15428 | 3 | 0.04% | 0.92% | 1.00% | 1 | 1 | 1 | 1 | 2 | 1 | 6-phosphofructo-2-kinase/fructose-2,6-biphosphatase 1 (6PF-2-K/Fru-2,6-P2ASE liver isozyme), transcript variant 1 |
| GB20120 | 26 | 0.04% | 0.00% | 0.00% | 1 | 1 | 1 | 1 | 1 | 1 | ACXD CG5712-PA (LOC551461), mRNA |
| GB17859 | 7 | 0.04% | 0.28% | 0.13% | 1 | 1 | 1 | 1 | 1 | 1 | CG5705-PA (LOC724133), mRNA |
| GB11349 | 3 | 0.04% | 0.78% | 1.00% | 1 | 1 | 1 | 1 | 1 | 1 | unc-104 CG8566-PD, transcript variant 1 (LOC413837), mRNA |
| GB15921 | 2 | 0.04% | 0.62% | 1.00% | 1 | 1 | 1 | 1 | 3 | 1 | slimfast CG11128-PC, isoform C (LOC409646), mRNA |
| GB19423 | 13 | 0.04% | 0.00% | 0.07% | 1 | 1 | 1 | 1 | 1 | 1 | CG7364-PA, transcript variant 1 (LOC552723), mRNA |
| GB19946 | 3 | 0.04% | 1.00% | 1.00% | 2 | 1 | 1 | 1 | 3 | 1 | CG3104-PA, isoform A (LOC726529), mRNA |
| GB15710 | 13 | 0.04% | 0.00% | 0.07% | 1 | 1 | 1 | 1 | 1 | 1 | two pore channel 1 (LOC413609), mRNA |
| GB13156 | 28 | 0.04% | 0.00% | 0.36% | 1 | 1 | 1 | 1 | 1 | 1 | CG6905-PA (LOC552527), mRNA |
| GB12863 | 4 | 0.04% | 0.73% | 1.00% | 1 | 1 | 2 | 1 | 1 | 7 | CG7394-PA (LOC726653), mRNA |
| GB11428 | 4 | 0.04% | 0.90% | 0.65% | 1 | 1 | 1 | 1 | 1 | 1 | Dual specificity phosphatase, catalytic domain. |
| GB17236 | 35 | 0.04% | 0.00% | 0.00% | 1 | 1 | 1 | 1 | 2 | 1 | CG7504-PA (LOC725908), mRNA |
| GB18858 | 8 | 0.04% | 0.61% | 0.07% | 1 | 1 | 1 | 1 | 1 | 1 | hypothetical protein LOC727329 (LOC727329), mRNA |
| GB11084 | 23 | 0.04% | 0.00% | 0.03% | 1 | 1 | 1 | 1 | 1 | 1 | AdoMet_MTase; S-adenosylmethionine-dependent methyltransferase (SAM or AdoMet-Mtase) |
| GB13434 | 5 | 0.04% | 0.38% | 0.74% | 1 | 1 | 1 | 1 | 1 | 1 | transcription factor IIB (LOC412363), mRNA |
| GB30265 | 7 | 0.04% | 0.00% | 0.78% | 1 | 1 | 1 | 1 | 2 | 1 | mutagen-sensitive 301 CG7972-PA (LOC724475), mRNA |
| GB12080 | 7 | 0.04% | 0.84% | 0.18% | 1 | 1 | 1 | 1 | 1 | 1 | CG12870-PA, transcript variant 1 (LOC551623), mRNA |
| GB10485 | 17 | 0.04% | 0.00% | 0.07% | 1 | 1 | 1 | 1 | 1 | 1 | CG3085-PA (LOC727267), mRNA |
| GB15111 | 13 | 0.04% | 0.00% | 0.48% | 1 | 1 | 1 | 1 | 1 | 1 | zyg-11 homolog B (C. elegans)-like (LOC409106), mRNA |
| GB19564 | 18 | 0.04% | 0.00% | 0.04% | 1 | 1 | 1 | 1 | 1 | 1 | CG18507-PA, isoform A (LOC412598), mRNA |
| GB19492 | 12 | 0.04% | 0.00% | 0.49% | 1 | 1 | 1 | 1 | 1 | 1 | GCN5 general control of amino-acid synthesis 5-like 2 (LOC552646), mRNA |
| GB12892 | 5 | 0.04% | 1.00% | 0.23% | 1 | 1 | 1 | 1 | 1 | 1 | Sox box protein at 102F ortholog |
| GB17103 | 30 | 0.04% | 0.00% | 0.18% | 1 | 1 | 1 | 1 | 1 | 1 | CARMIl (cytoskeleton protein) |
| GB12585 | 3 | 0.04% | 1.00% | 1.00% | 1 | 1 | 1 | 1 | 1 | 1 | ephrin receptor (Eph), mRNA |
| GB14588 | 39 | 0.04% | 0.00% | 0.00% | 1 | 1 | 1 | 1 | 8 | 1 | eukaryotic translation initiation factor 4E nuclear import factor 1 (LOC727230), mRNA |
| GB12610 | 3 | 0.04% | 0.01% | 0.19% | 1 | 1 | 1 | 1 | 1 | 1 | Spef is a region of sperm flagellar proteins |
| GB10565 | 3 | 0.04% | 0.90% | 1.00% | 1 | 1 | 1 | 1 | 1 | 1 | tribbles homolog 2 (LOC409288), mRNA |
| GB15516 | 18 | 0.04% | 0.00% | 0.48% | 1 | 1 | 1 | 1 | 1 | 1 | CG4089-PA (LOC724974), mRNA |
| GB13475 | 17 | 0.04% | 0.00% | 0.00% | 1 | 1 | 1 | 1 | 3 | 1 | centaurin beta 1A CG6742-PA, isoform A (LOC409230), mRNA |
| GB19987 | 7 | 0.04% | 0.00% | 0.00% | 1 | 1 | 1 | 1 | 1 | 1 | ninjurin A CG6449-PA, isoform A (LOC725779), mRNA |
| GB15770 | 4 | 0.04% | 0.00% | 0.65% | 1 | 1 | 1 | 1 | 1 | 1 | CG5073-PA (LOC412160), mRNA |
| GB12814 | 35 | 0.04% | 0.00% | 0.00% | 1 | 1 | 1 | 1 | 1 | 1 | Vinculin |
| GB16966 | 9 | 0.04% | 0.40% | 0.21% | 1 | 1 | 1 | 1 | 2 | 1 | RNA polymerase II elongation factor ELL2 (LOC409928), mRNA |
| GB18192 | 15 | 0.04% | 0.00% | 0.13% | 2 | 1 | 1 | 1 | 1 | 1 | CG6453-PA (LOC552747), mRNA |
| GB14753 | 2 | 0.04% | 0.47% | 1.00% | 1 | 1 | 1 | 1 | 1 | 1 | olf186-F CG11430-PB, isoform B (LOC412524), mRNA |
| GB10433 | 37 | 0.04% | 0.00% | 0.00% | 1 | 1 | 1 | 1 | 2 | 1 | RhoGAPp190 CG32555-PA, isoform A (LOC551731), mRNA |
| GB18386 | 31 | 0.04% | 0.00% | 0.42% | 1 | 1 | 1 | 1 | 1 | 2 | CG2063-PA (LOC726957), mRNA |
| GB17337 | 7 | 0.04% | 0.49% | 0.65% | 1 | 1 | 1 | 1 | 1 | 1 | DnaB helicase C terminal domain |
| GB30058 | 3 | 0.04% | 0.97% | 1.00% |  |  |  |  |  |  | headcase |
| GB10792 | 3 | 0.04% | 1.00% | 1.00% | 1 | 1 | 1 | 1 | 1 | 1 | CG5169-PA, transcript variant 1 (LOC410427), mRNA |
| GB17451 | 2 | 0.04% | 0.39% | 1.00% | 1 | 1 | 1 | 1 | 1 | 1 | CDK5 regulatory subunit associated protein 3 (LOC727480), mRNA |
| GB13158 | 49 | 0.04% | 0.00% | 0.00% | 1 | 1 | 1 | 1 | 3 | 1 | CTD-binding SR-like protein rA9 (LOC727274), mRNA |
| GB18867 | 37 | 0.04% | 0.00% | 0.00% | 1 | 1 | 1 | 1 | 1 | 1 | HECT, UBA and WWE domain containing 1 (LOC411961), mRNA |
| GB11884 | 7 | 0.04% | 0.94% | 0.23% | 1 | 1 | 1 | 1 | 1 | 1 | CG14301-PA (LOC411343), mRNA |
| GB16867 | 2 | 0.04% | 0.20% | 1.00% | 1 | 1 | 1 | 1 | 1 | 1 | TBOX |
| GB11252 | 13 | 0.04% | 0.00% | 0.00% |  |  |  |  |  |  | MFS |
| GB17317 | 25 | 0.04% | 0.00% | 0.00% | 1 | 1 | 1 | 1 | 1 | 1 | Chromosome-associated protein CG9802-PA, isoform A (LOC410217), mRNA |
| GB11551 | 2 | 0.04% | 0.86% | 1.00% | 1 | 1 | 1 | 1 | 1 | 1 | some similarity to Centrosomal protein of 135 kDa, putative [Pediculus humanus] |
| GB19367 | 3 | 0.04% | 0.39% | 1.00% | 1 | 1 | 1 | 1 | 1 | 1 | soluble guanylyl cyclase alpha 1 subunit (AmsGC alpha-1), mRNA |
| GB10701 | 3 | 0.04% | 0.38% | 1.00% | 1 | 1 | 1 | 1 | 1 | 1 | hypothetical LOC409327 (LOC409327), mRNA |
| GB12100 | 14 | 0.04% | 0.00% | 0.10% | 1 | 1 | 1 | 1 | 1 | 1 | CG6133-PA (LOC411579), mRNA |
| GB11773 | 16 | 0.04% | 0.00% | 0.17% | 1 | 1 | 2 | 1 | 1 | 1 | Helix-loop-helix domain, found in specific DNA- binding proteins that act as TFs plus Kri domain |
| GB11779 | 3 | 0.04% | 0.92% | 1.00% | 1 | 1 | 1 | 1 | 1 | 1 | CG9650-PB, isoform B (LOC724150), mRNA |
| GB13825 | 2 | 0.04% | 0.99% | 1.00% | 1 | 1 | 1 | 1 | 1 | 1 | GA13134-PA [Nasonia vitripennis] |
| GB16866 | 3 | 0.04% | 0.96% | 1.00% | 1 | 1 | 1 | 1 | 1 | 1 | Homeotic protein ocelliless (Protein orthodenticle) (LOC410684), mRNA |
| GB13405 | 37 | 0.04% | 0.00% | 0.00% | 1 | 1 | 1 | 1 | 1 | 1 | CG11376-PA (LOC411245), mRNA |
| GB12366 | 13 | 0.04% | 0.00% | 0.05% | 1 | 1 | 1 | 1 | 2 | 1 | CG7065-PA (LOC551475), mRNA |
| GB11691 | 15 | 0.04% | 0.00% | 0.00% | 1 | 1 | 1 | 1 | 1 | 1 | CG13624-PC, isoform C, transcript variant 1 (LOC413653), mRNA |
| GB13240 | 24 | 0.04% | 0.00% | 0.00% | 1 | 1 | 1 | 1 | 1 | 1 | CG12360-PA, isoform A (LOC413724), mRNA |
| GB16452 | 20 | 0.04% | 0.00% | 0.15% | 2 | 2 | 1 | 4 | 9 | 1 | CG5525-PA, transcript variant 1 (LOC551275), mRNA |
| GB16956 | 4 | 0.04% | 0.00% | 0.29% | 1 | 1 | 1 | 1 | 1 | 1 | Dihydroorotate dehydrogenase, mitochondrial precursor (Dihydroorotate oxidase) (DHOdehase) |
| GB17279 | 2 | 0.04% | 0.44% | 1.00% | 1 | 1 | 1 | 1 | 1 | 1 | CG7095-PA (LOC725485), mRNA |
| GB16699 | 13 | 0.04% | 0.00% | 0.82% | 4 | 3 | 1 | 2 | 8 | 3 | CG11444-PA (LOC724487), mRNA |
| GB12920 | 20 | 0.04% | 0.00% | 0.01% | 1 | 2 | 1 | 1 | 1 | 1 | Minichromosome maintenance 5 CG4082-PA (LOC551903), mRNA |
| GB17310 | 3 | 0.04% | 0.01% | 1.00% | 1 | 1 | 1 | 1 | 1 | 1 | sushi, von Willebrand factor type A, EGF and pentraxin domain-containing |
| GB18129 | 26 | 0.04% | 0.00% | 0.00% | 1 | 1 | 1 | 1 | 1 | 1 | Arginine methyltransferase 4 CG5358-PA (LOC411458), mRNA |
| GB16217 | 20 | 0.04% | 0.00% | 0.07% | 1 | 1 | 1 | 1 | 1 | 1 | MYPT-75D CG6896-PA (LOC411548), mRNA |
| GB16176 | 30 | 0.04% | 0.00% | 0.00% | 1 | 1 | 1 | 1 | 2 | 1 | nadrin (LOC411535), mRNA |
| GB10253 | 2 | 0.05% | 0.93% | 1.00% | 10 | 8 | 4 | 9 | 6 | 10 | CG17280-PA (LOC726042), mRNA |
| GB18828 | 11 | 0.05% | 0.00% | 0.08% | 2 | 1 | 1 | 1 | 2 | 1 | COP9 complex homolog subunit 7 CG2038-PA (LOC411843), mRNA |
| GB10959 | 28 | 0.05% | 0.00% | 0.00% | 1 | 1 | 1 | 1 | 1 | 1 | 26S proteasome regulatory subunit rpn2 |
| GB13357 | 2 | 0.05% | 0.86% | 1.00% | 1 | 1 | 1 | 1 | 1 | 1 | hypothetical protein LOC727039 (LOC727039), mRNA |
| GB11403 | 2 | 0.05% | 0.29% | 1.00% | 2 | 1 | 1 | 1 | 1 | 1 | CG6414-PA (LOC408395), mRNA |
| GB30491 | 3 | 0.05% | 1.00% | 1.00% |  |  |  |  |  |  | MAX dimerization protein |
| GB18117 | 2 | 0.05% | 0.88% | 1.00% | 1 | 1 | 1 | 1 | 1 | 1 | basic region leucin zipper |
| GB12768 | 45 | 0.05% | 0.00% | 0.00% | 1 | 1 | 1 | 1 | 1 | 1 | kinesin family member 21A, transcript variant 1 (LOC411068), mRNA |
| GB16436 | 14 | 0.05% | 0.00% | 0.09% | 1 | 1 | 1 | 1 | 4 | 1 | CG10283-PA, isoform A (LOC411435), mRNA |
| GB18982 | 9 | 0.05% | 0.00% | 0.44% | 1 | 1 | 1 | 1 | 1 | 1 | anaphase-promoting complex subunit 4 (LOC409810), mRNA |
| GB15779 | 3 | 0.05% | 0.58% | 1.00% | 3 | 1 | 1 | 3 | 5 | 1 | Zwischenferment CG12529-PA, isoform A (LOC725325), mRNA |
| GB14293 | 8 | 0.05% | 0.21% | 0.00% | 1 | 1 | 1 | 1 | 1 | 1 | DEAH (Asp-Glu-Ala-His) box polypeptide 30 isoform 2 (LOC726077), partial mRNA |
| GB30055 | 2 | 0.05% | 0.84% | 0.30% |  |  |  |  |  |  | a similar protein in Nasonia |
| GB11315 | 58 | 0.05% | 0.00% | 0.00% | 1 | 1 | 1 | 1 | 1 | 1 | CG17766-PA, transcript variant 1 (LOC412288), mRNA |
| GB14667 | 4 | 0.05% | 0.81% | 0.55% | 1 | 1 | 1 | 1 | 1 | 1 | solute carrier family 24, member 5 (LOC412316), mRNA |
| GB12207 | 3 | 0.05% | 0.88% | 1.00% | 1 | 1 | 1 | 1 | 1 | 1 | EGFR epidermal growth factor receptor |
| GB18938 | 19 | 0.05% | 0.00% | 0.16% | 1 | 1 | 1 | 1 | 4 | 1 | CG9281-PB, isoform B (LOC413252), mRNA |
| GB13772 | 15 | 0.05% | 0.00% | 0.11% | 1 | 1 | 1 | 1 | 1 | 1 | Puromycin sensitive aminopeptidase CG1009-PC, isoform C, transcript variant 1 (LOC410769), mRNA |
| GB15134 | 33 | 0.05% | 0.00% | 0.00% | 1 | 1 | 1 | 1 | 1 | 2 | CG1109-PA, isoform A (LOC413618), mRNA |
| GB14880 | 6 | 0.05% | 0.03% | 0.58% | 1 | 1 | 1 | 1 | 2 | 1 | CG17683-PA.3 (LOC724370), mRNA |
| GB18292 | 18 | 0.05% | 0.00% | 0.30% | 2 | 1 | 1 | 2 | 3 | 1 | 26S protease regulatory subunit 8, transcript variant 1 (LOC550794), mRNA |
| GB16141 | 29 | 0.05% | 0.00% | 0.11% | 1 | 1 | 1 | 1 | 1 | 1 | Adaptin CG12532-PA (LOC726187), mRNA |
| GB16155 | 11 | 0.05% | 0.02% | 0.25% | 1 | 1 | 1 | 1 | 1 | 1 | CG18028-PA.3 (LOC551708), mRNA |
| GB10205 | 3 | 0.05% | 0.11% | 1.00% | 1 | 1 | 1 | 1 | 1 | 1 | lethal (3) IX-14 CG3953-PA (LOC409923), mRNA |
| GB10655 | 6 | 0.05% | 0.01% | 0.01% | 1 | 1 | 1 | 1 | 1 | 1 | cactus CG5848-PB, isoform B (LOC725124), mRNA |
| GB18282 | 2 | 0.05% | 0.97% | 1.00% | 1 | 1 | 1 | 1 | 1 | 1 | formin 3 CG33556-PA (LOC408821), mRNA |
| GB15344 | 23 | 0.05% | 1.00% | 0.00% | 1 | 1 | 1 | 1 | 1 | 1 | Leukocyte-antigen-related-like CG10443-PA (LOC413569), mRNA |
| GB17965 | 10 | 0.05% | 0.00% | 0.00% | 2 | 1 | 1 | 2 | 2 | 1 | CG17593-PA (LOC412935), mRNA (Coiled-coil domain containing 47 ) |
| GB16863 | 3 | 0.05% | 0.88% | 1.00% | 1 | 1 | 1 | 1 | 1 | 1 | Nuclear Hormone Receptor family member (nhr-91) (LOC410925), mRNA |
| GB11590 | 21 | 0.05% | 0.00% | 0.00% | 1 | 1 | 1 | 1 | 1 | 1 | CG6565-PA (LOC552633), mRNA |
| GB16330 | 40 | 0.05% | 0.00% | 0.00% | 1 | 1 | 1 | 1 | 1 | 1 | trithorax CG8651-PD, isoform D (LOC408716), mRNA |
| GB18947 | 4 | 0.05% | 1.00% | 1.00% | 1 | 1 | 1 | 1 | 1 | 1 | BRCT domain found in BRCA1 |
| GB16457 | 10 | 0.05% | 0.54% | 0.04% | 1 | 1 | 1 | 1 | 1 | 1 | u-shaped, zinc finger protein |
| GB15732 | 19 | 0.05% | 0.00% | 0.00% | 1 | 1 | 1 | 1 | 2 | 1 | CG6686-PB, isoform B, transcript variant 1 (LOC409347), mRNA |
| GB19921 | 9 | 0.05% | 0.00% | 0.05% | 1 | 1 | 1 | 1 | 2 | 1 | retinoblastoma-binding protein 6 isoform 2 (LOC724670), partial mRNA |
| GB16120 | 21 | 0.05% | 0.00% | 0.01% | 1 | 1 | 1 | 1 | 1 | 1 | belphegor CG6815-PA (LOC551329), mRNA |
| GB11144 | 4 | 0.05% | 0.00% | 0.82% | 1 | 1 | 1 | 1 | 1 | 2 | mitochondrial ribosomal protein L51 CG13098-PA (LOC724263), mRNA |
| GB13989 | 23 | 0.05% | 0.00% | 0.02% | 1 | 1 | 1 | 1 | 2 | 1 | CG31694-PA (LOC409368), mRNA |
| GB19928 | 12 | 0.05% | 0.37% | 0.00% | 1 | 1 | 1 | 1 | 1 | 1 | WW, C2 and coiled-coil domain containing 1 (LOC413440), mRNA |
| GB17583 | 24 | 0.05% | 0.00% | 0.00% | 1 | 1 | 1 | 1 | 1 | 1 | Transportin-Serine/Arginine rich CG2848-PA (LOC726159), mRNA |
| GB30511 | 5 | 0.05% | 0.14% | 0.14% | 1 | 1 | 1 | 1 | 2 | 1 | CG33138-PA (LOC727168), partial mRNA |
