# Supplementary material for: The Honey Bee Epigenomes: Differential Methylation of Brain DNA in Queens and Workers
Source: PLoS Biol. 2010 Nov 2;8(11):e1000506. doi: 10.1371/journal.pbio.1000506 (PMC2970541; doi:10.1371/journal.pbio.1000506)
Supplement: Table S3 — Evaluation of the Elango et al. hypothesis. (A) CpG o/e in methylated genes. (B) Differential methylation and differential gene expression. (0.04 MB DOC) [file pbio.1000506.s013.doc]

Table S3A: CpG o/e in methylated genes

|  | **Methylation status** | **Low CpG o/e** | **High CpG o/e** | **P-value (Fisher exact test)** |
| --- | --- | --- | --- | --- |
| **All genes** | Methylated | 5195 | 643 | < 10^-200^ |
|  | Non-Methylated | 1391 | 3489 |  |
| **Caste-specific genes** | Methylated | 356 | 52 | 6.6 x 10^-66^ |
|  | Non-Methylated | 69 | 215 |  |
| **Differentially methylated, compared to all genes** | Methylated | 393 | 156 | 3.0 x 10^-6^ |
|  | Non-Methylated | 6273 | 3877 |  |
| **Differentially methylated compared to methylated genes** | Methylated | 393 | 156 | 5.7 x 10^-28^ |
|  | Non-Methylated | 4823 | 561 |  |

Table S3B

**Differential Methylation and Differential Gene Expression**

We investigated the relationship between the differentially methylated genes identified in this study and the “caste-specific” genes expressed in the brain reported by previous studies (Elango et al 2009, Grozinger et al 2007). We found that differential methylation does not correlate with caste-specific expression (table Y2).

Table S3B: Caste specific gene expression and differential methylation. There is no statistically significant difference between these categories (Fisher exact test, p=0.53).

|  | Differentially methylated | Not differentially methylated |
| --- | --- | --- |
| Caste specific | 50 | 620 |
| Caste generic | 92 | 1011 |
